# Inhibition of osimertinib-resistant epidermal growth factor receptor EGFR-T790M/C797S†
†Electronic supplementary information (ESI) available. CCDC 1876852. For ESI and crystallographic data in CIF or other electronic format see DOI: 10.1039/c9sc03445e


**DOI:** 10.1039/c9sc03445e

**Published:** 2019-10-04

**Authors:** Jonas Lategahn, Marina Keul, Philip Klövekorn, Hannah L. Tumbrink, Janina Niggenaber, Matthias P. Müller, Luke Hodson, Maren Flaßhoff, Julia Hardick, Tobias Grabe, Julian Engel, Carsten Schultz-Fademrecht, Matthias Baumann, Julia Ketzer, Thomas Mühlenberg, Wolf Hiller, Georgia Günther, Anke Unger, Heiko Müller, Alena Heimsoeth, Christopher Golz, Bernhard Blank-Landeshammer, Laxmikanth Kollipara, René P. Zahedi, Carsten Strohmann, Jan G. Hengstler, Willem A. L. van Otterlo, Sebastian Bauer, Daniel Rauh

**Affiliations:** a Faculty of Chemistry and Chemical Biology , TU Dortmund University , Otto-Hahn-Strasse 4a , 44227 Dortmund , Germany . Email: daniel.rauh@tu-dortmund.de ; www.twitter.com/DDHDortmund ; Tel: +49-231-755-7080; b Drug Discovery Hub Dortmund (DDHD), Zentrum für Integrierte Wirkstoffforschung (ZIW) , 44227 Dortmund , Germany; c Department of Chemistry and Polymer Science , Stellenbosch University , Private Bag X1 , Matieland 7602 , South Africa; d Lead Discovery Center GmbH , Otto-Hahn-Strasse 15 , 44227 Dortmund , Germany; e Department of Medical Oncology , Sarcoma Center , West German Cancer Center , University Duisburg-Essen , Medical School , Hufelandstrasse 55 , 45122 Essen , Germany; f German Cancer Consortium (DKTK) , 69120, Heidelberg , Germany; g Leibniz Research Centre for Working Environment and Human Factors (IfADo) , TU Dortmund University , Ardeystrasse 67 , 44139 Dortmund , Germany; h Molecular Pathology , Institute of Pathology , University Hospital of Cologne , Kerpener Strasse 62 , 50937 Cologne , Germany; i Department of Translational Genomics , Center of Integrated Oncology Cologne-Bonn , Medical Faculty , University of Cologne , Weyertal 115b , 50931 Cologne , Germany; j Leibniz-Institut für Analytische Wissenschaften – ISAS – e.V. , Otto-Hahn-Strasse 6b , 44227 Dortmund , Germany; k Segal Cancer Proteomics Centre , Lady Davis Institute , Jewish General Hospital , McGill University , 3755 Côte Ste-Catherine Road , Montreal , Quebec H3T 1E2 , Canada

## Abstract

Precision medicine has revolutionized the treatment of patients in EGFR driven non-small cell lung cancer (NSCLC). Targeted drugs show high response rates in genetically defined subsets of cancer patients and markedly increase their progression-free survival as compared to conventional chemotherapy. However, recurrent acquired drug resistance limits the success of targeted drugs in long-term treatment and requires the constant development of novel efficient inhibitors of drug resistant cancer subtypes. Herein, we present covalent inhibitors of the drug resistant gatekeeper mutant EGFR-L858R/T790M based on the pyrrolopyrimidine scaffold. Biochemical and cellular characterization, as well as kinase selectivity profiling and western blot analysis, substantiate our approach. Moreover, the developed compounds possess high activity against multi drug resistant EGFR-L858R/T790M/C797S in biochemical assays due to their highly reversible binding character, that was revealed by characterization of the binding kinetics. In addition, we present the first X-ray crystal structures of covalent inhibitors in complex with C797S-mutated EGFR which provide detailed insight into their binding mode.

## Abstract 2

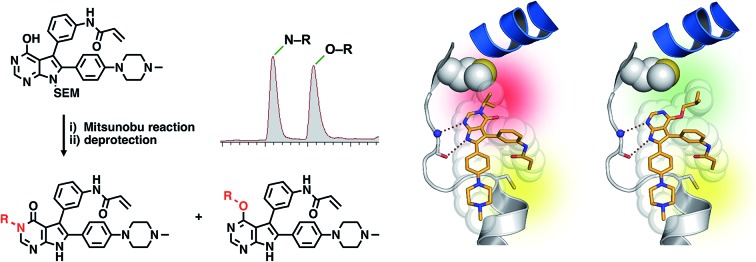
We present inhibitors of drug resistant mutants of EGFR including T790M and C797S. In addition, we present the first X-ray crystal structures of covalent inhibitors in complex with C797S-mutated EGFR to gain insight into their binding mode.

## Introduction

Ligand-induced EGFR receptor tyrosine kinase activation promotes downstream signaling which triggers cell proliferation and survival.^1^,^2^ Sensitizing mutations such as L858R or delE746_A750 in the EGFR kinase domain render the receptor constitutively activated, independent of an extracellular ligand-binding event.^3^–^5^ Sustained hyper-activated downstream signaling pathways therefore drive tumorigenesis and result in the emergence of non-small cell lung cancer.^6^ Targeting EGFR with small-molecule inhibitors, such as gefitinib^7^,^8^ or erlotinib,^9^ has proved to be successful in cancer therapy, improving progression-free survival of patients suffering from EGFR-mutant NSCLC as compared to cytotoxic chemotherapy.^10^–^17^ However, the efficacy of these first generation inhibitors has been limited due to the emergence of drug resistance within the first year of treatment. Acquired resistance to these targeted drugs is caused by a secondary mutation in EGFR (T790M) at the gatekeeper position that occurs in 60% of the patients,^18^ inducing steric hindrance to the first generation inhibitors and thereby preventing inhibitor binding.^19^

Second generation inhibitors like afatinib^20^,^21^ were designed to covalently target a reactive cysteine (Cys797) at the lip of the ATP-binding site. To this end, an acrylamide moiety on the inhibitor in close proximity to Cys797 was available to undergo a Michael addition. Thereby, competition with the co-factor ATP is reduced and the drug–target residence time is prolonged, resulting in increased inhibitory potency.^22^–^24^ These agents inhibited EGFR-T790M *in vitro*,^20^ but unfortunately failed to induce convincing response rates in clinical trials. It was found that simultaneous EGFR-wt inhibition observed with these compounds narrows their therapeutic window, since high dosing results in toxic events and causes severe side effects.^25^,^26^

Both generations of inhibitors incorporate 4-amino quinazolines that were originally developed to target wild type EGFR. In order to circumvent the advent of side effects, the following features were found to be crucial: (i) employing novel scaffolds that allow substitutions which do not sterically interfere with Met790, thereby (ii) being mutant-selective and sparing wild type inhibition, and (iii) incorporation of a reactive substituent to alkylate Cys797 in EGFR to achieve a maximum drug–target residence time.^27^ Accordingly, pyrimidine-based third generation inhibitors rociletinib/CO-1686,^28^ osimertinib/AZD9291,^29^–^31^ and olmutinib/HM61713 32 were introduced and displayed promising results at the clinical stage.^33^–^36^ Rociletinib was discontinued due to a metabolite that interfered with blood glucose levels,^37^ but the latter two drugs, osimertinib and olmutinib, have been approved for the treatment of T790M-positive patients.^38^,^39^ Further compounds from this therapeutic class are currently being tested in clinical settings, among them nazartinib/EGF816.^40^

However, third generation inhibitors suffer from drug resistance that emerges within the first year of treatment from the mutation of the non-catalytic cysteine (C797S), which is the target amino acid modified in a covalent fashion.^41^–^46^ The efficiency of these inhibitors is mainly based on the bond formation with the target protein, but reversible interactions within the binding site are required to efficiently inhibit cysteine mutant EGFR.^47^ Accordingly, compounds under investigation have been described that inhibit EGFR-C797S in biochemical settings,^47^–^53^ but to date no inhibitor that acts as a single agent to affect osimertinib-resistant tumors *in vivo* has been described.^54^–^56^

We therefore set out to establish novel scaffolds for designing selective inhibitors that are effective against multi-drug resistant EGFR. To this end, we employed the pyrrolopyrimidine core that can be equipped with a phenylacrylamide, resulting in this electrophile being in close proximity to Cys797. We found that this scaffold offered fast access to derivatives utilizing the Mitsunobu reaction, resulting in an easily separable mixture of 3-substituted pyrrolopyrimidin-4-ones and 4-substituted pyrrolopyrimidines. Characterization in biochemical assays as well as cellular studies and western blot analysis revealed the potency of the so-obtained inhibitors in EGFR gatekeeper mutant cell lines. Although we recently succeeded in solving a series of complex crystal structures in drug resistant EGFR-T790M (PDB IDs: 5J9Y and ; 5J9Z),^47^ the herein developed compounds did not give crystals suitable to collect high-resolution diffraction data. Therefore, we decided to solve the structures in complex with the T338M/S345C mutant cSrc, a surrogate we have used successfully in the past^19^,^57^ to gain insights into the binding mode of differentially substituted pyrrolopyrimidines. Characterization of the kinetics of covalent bond formation showed the intensely reversible character of the inhibitor–protein interaction and, accordingly, its high potency against the C797S mutant variant of EGFR was observed in biochemical assays. To our delight, we were able to solve two co-crystal structures of potent inhibitors in complex with EGFR-T790M/C797S. This is the first report of X-ray crystal structures with covalent inhibitors reversibly binding to C797S drug resistant EGFR. These studies have provided insight into the binding characteristics and revealed the superiority of 4-substituted pyrrolopyrimidines over 3-substituted pyrrolopyrimidin-4-ones due to less steric hindrance with the methionine gatekeeper side chain. Moreover, the effect of the spatial size of the moiety in 4-position on the conformation of the inhibitor was found to facilitate efficient covalent binding of Cys797.

## Results

### Rational design, synthesis, and biological testing of the first set of pyrrolopyrimidine EGFR inhibitors with optimized solubility and cell permeability

By conducting structural analyses, synthesis, and subsequent biological testing of the designed molecules in an iterative process and with guidance from X-ray crystallography, we developed a series of potent inhibitors of mutant EGFR.

**Fig. 1:**
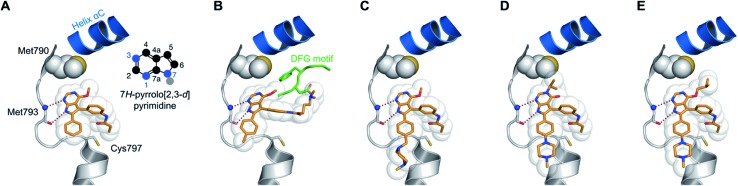
Structural analysis of selected substituted pyrrolopyrimidine-based EGFR inhibitors (based on PDB ID 2JIU aligned to the binding site of apo EGFR-T790M, PDB ID ; 3UG1). (A) Initial compound **1a**, (B) compound **10a** with *N*,*N*-dimethylamino crotonic amide as Michael acceptor. The solubilizing group might interact with the DFG motif (based on PDB ID ; 4JRV), (C) compound **17a** with methyl piperazine solubilizing group, (D) *N*3 iso-propyl substituted pyrrolopyrimidin-4-one **29g**, and (E) *O*4 iso-butyl substituted pyrrolopyrimidine **19h**. Hydrogen-bond interactions of the inhibitors with the hinge region (white) are illustrated by red dotted lines. The helix C is displayed in blue and the DFG motif is shown in green.

We analyzed known co-crystal structures of pyrrolopyrimidine ligands with kinases in the Protein Data Bank (PDB) and generated structures of the anticipated binding modes by alignment to T790M-mutated apo EGFR (Fig. 1). We found that the pyrrolopyrimidine core formed bidentate hydrogen bonds to Met793 of the kinase hinge region and was anchored by a phenyl moiety in the 6-position to orient the scaffold in the binding site (PDB ID: ; 2JIU). In this structural analysis, the 5-position was found to be suitable for the introduction of a phenyl linker that could be equipped with an acrylamide in the *ortho*-position as the reactive group in close proximity to Cys797. A methoxy group in the 4-position was thought to be small enough to avoid negative interference with the side chain of Met790 (Fig. 1A).

The synthetic route giving rise to the designed compounds was optimized during the course of compound development. The first set of above mentioned compounds, such as **1a**, was synthesized starting from commercially available 6-bromo-4-chloro-7*H*-pyrrolo[2,3-*d*]pyrimidine (**2**). Nucleophilic aromatic substitution with sodium methoxide followed by protection of the pyrrole NH resulted in compound **4**. Suzuki–Miyaura coupling introduced the phenyl moiety in the 6-position and the following iodination allowed for another Suzuki–Miyaura coupling with *meta*-nitrobenzeneboronic acid, resulting in compound **7**. The amine **8** was obtained by reduction of the nitro group and was transformed into the acrylamide or the propionamide with the respective acid chloride. Deprotection yielded the desired compound **1a** and the respective reversible counterpart **1b** (Scheme S1†).

To our delight, compound **1a** inhibited the EGFR-wt kinase with an IC_50_ of ∼1 μM when tested in our activity-based biochemical assay and displayed an almost 10-fold selectivity for the L858R (IC_50_ = 133 nM) and L858R/T790M (IC_50_ = 176 nM) mutant variants. Moreover, the reversible counterpart **1b** showed an intense drop in activity, with IC_50_ values of 5 μM against wild type and 4 μM against the gatekeeper mutant EGFR (Table 1), indicating the importance of covalent bond formation for inhibitory activity. We subsequently tested these compounds against cancer cell lines in a CellTiter-Glo assay, which revealed a poor influence on cellular viability with EC_50_ values of more than 10 μM. Here, we employed the wild type-bearing cell line A431, EGFR-delE746_A750 activating mutation-carrying HCC827 cells, and the EGFR-L858R/T790M drug resistant cell line H1975 (Table 1). Additionally, A549 and H358 cell lines were tested; these lines carried mutant KRAS and were used to indicate cytotoxicity and EGFR off-target inhibition (Table S2†).

**Table 1:** Overview of the first set of pyrrolopyrimidine EGFR inhibitors and corresponding IC_50_ and EC_50_ determinations on different EGFR mutant variants and corresponding NSCLC cell lines^*a*^

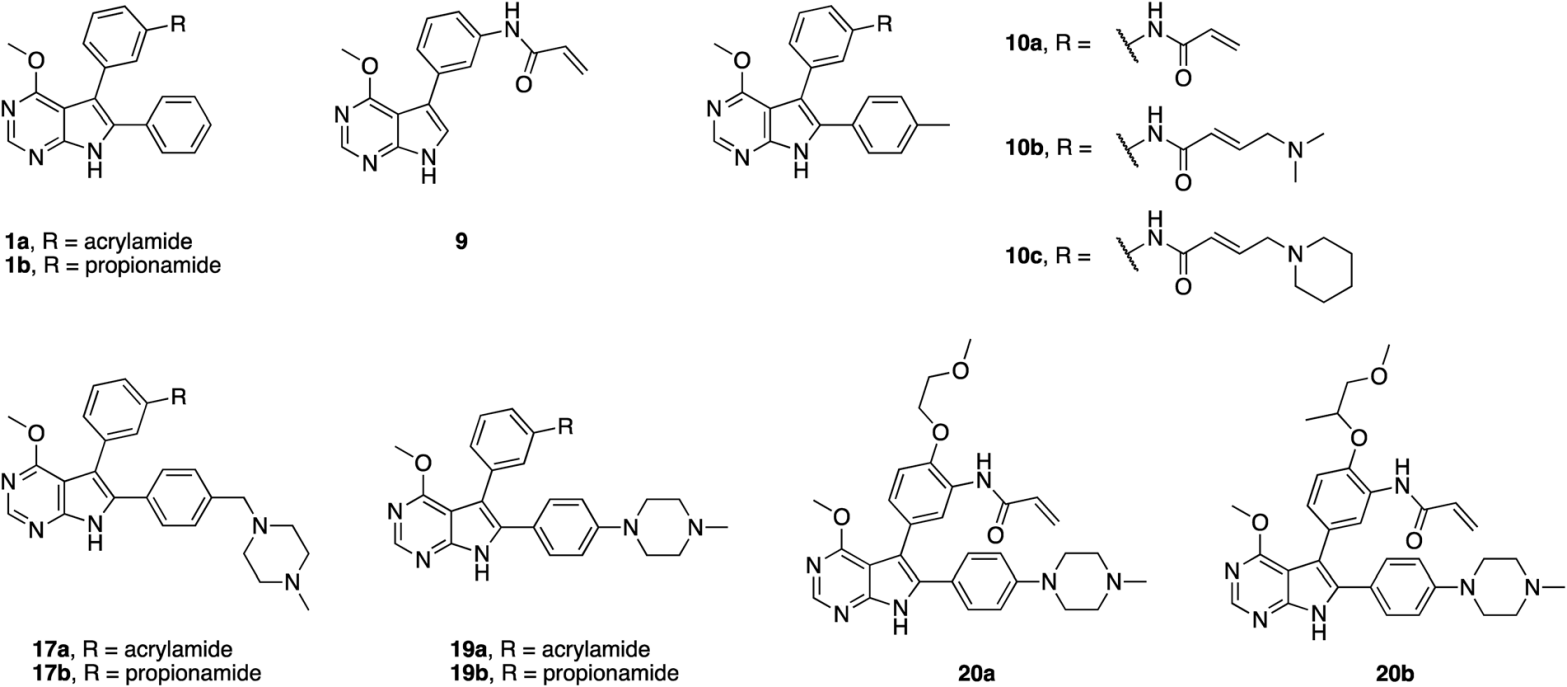						
Cpd	EGFR HTRF IC_50_ [nM]			EGFR CTG EC_50_ [nM]		
	wt	L858R	L858R/T790M	A431	HCC827	H1975
**1a**	1203 ± 87	133 ± 57	176 ± 33	>30 000	827 ± 234	12 169 ± 1157
**1b**	5372 ± 1919	1703 ± 747	3963 ± 614	>30 000	19 113 ± 2505	>30 000
**9**	82 ± 4	14 ± 2	31 ± 4	14 033 ± 4749	2168 ± 968	9912 ± 3389
**10a**	200 ± 148	93 ± 54	175 ± 44	9865 ± 4247	151 ± 10	2043 ± 88
**10b**	60 ± 18	34 ± 11	26 ± 8	2433 ± 991	282 ± 29	980 ± 136
**10c**	220 ± 57	197 ± 11	158 ± 16	2746 ± 1215	253 ± 1	1902 ± 269
**17a**	15 ± 10	2.3 ± 0.4	4.0 ± 1.7	768 ± 271	<14	137 ± 51
**17b**	460 ± 162	116 ± 57	302 ± 84	5666 ± 60	1516 ± 399	9613 ± 1117
**19a**	0.4 ± 0.0	0.1 ± 0.0	0.2 ± 0.0	638 ± 247	<14	60 ± 8
**19b**	996 ± 395	147 ± 97	305 ± 192	9732 ± 4015	1794 ± 727	13 891 ± 1777
**20a**	0.2 ± 0.1	<0.1	<0.1	27 058 ± 5096	<14	298 ± 251
**20b**	0.2 ± 0.2	<0.1	0.1 ± 0.1	1324 ± 777	<14	375 ± 252
Gefitinib	0.2 ± 0.1	<0.1	185 ± 98	1709 ± 792	<14	10 733 ± 2550
AEE788	2.5 ± 4.9	0.2 ± 0.2	296 ± 205	1788 ± 1094	16 ± 3	4683 ± 1033
Afatinib	<0.1	<0.1	0.3 ± 0.1	634 ± 312	<14	653 ± 109
WZ4002	9.6 ± 7.0	0.4 ± 0.3	0.2 ± 0.1	2139 ± 439	20 ± 6	97 ± 37
Rociletinib	10 ± 1	2.0 ± 0.1	0.3 ± 0.1	1911 ± 467	45 ± 11	145 ± 86
Osimertinib	1.0 ± 0.6	0.7 ± 0.6	0.3 ± 0.0	756 ± 340	<14	16 ± 5
EGF816	1.7 ± 0.8	0.6 ± 0.3	0.3 ± 0.1	4381 ± 1390	<14	296 ± 64

^*a*^Values are the mean ± SD of three independent measurements in duplicates.

Initially, we hypothesized that the 6-phenyl moiety served as an anchor to allow the pyrrolopyrimidine core to bind in the assumed fashion. We synthesized compound **9**, with no substitution in the 6-position, as well as compound **10a** possessing a *para*-tolyl substituent. 4-Chloro-7*H*-pyrrolo[2,3-*d*]pyrimidine (**11**) was transformed into compound **14** similarly to the described synthetic route. By installing the bromo halogen in the 6-position and subsequent Suzuki–Miyaura coupling with {4-[(4-methylpiperazin-1-yl)methyl]phenyl}boronic acid, compound **15** was synthesized. Under harsh reductive conditions this compound gave the *para*-tolyl bearing amine **16**. Treatment with acryloyl chloride and subsequent deprotection yielded the final compound **10a** (Scheme S2†). Using the established synthetic procedures, compound **14** was transformed into the desired unfunctionalized compound **9**.

**Fig. 2:**
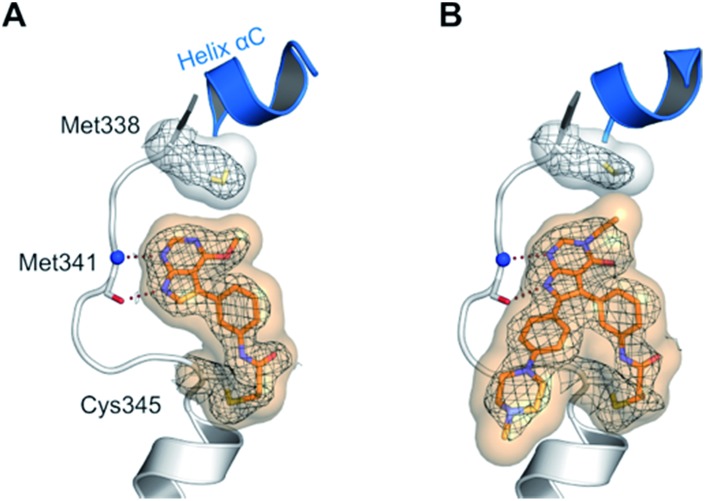
Co-crystal structures of pyrrolopyrimidines in complex with engineered cSrc-T338M/S345C, a reliable model system for the EGFR-T790M mutant. Diagrams of the experimental electron densities of (A) **9**/cSrc-T338M/S345C at 1.9 Å (PDB ID: ; 6HVE), (B) **29b**/cSrc-T338M/S345C at 2.1 Å resolution (PDB ID: ; 6HVF); 2Fo-Fc map contoured at an r.m.s.d. of 1.

Interestingly, both compounds showed an intense gain in potency at the biochemical level with an IC_50_ below 200 nM for **10a**, and even more remarkably, below 100 nM for compound **9** against EGFR-L858R/T790M (Table 1). However, we were able to solve the crystal structure of **9** in complex with cSrc-T338M/S345C, a model system for EGFR-T790M (Fig. 2A and S1A;† PDB ID: ; 6HVE).^19^,^57^ Interestingly, refined structures revealed a binding mode similar to 6-substituted pyrrolopyrimidines with bidentate hydrogen bonds to Met341 and the acrylamide covalently bound to Cys345 (these residues correspond to Met793 and Cys797, respectively, in EGFR). Contrasting with our initial assumption, these data show that the 6-phenyl residue might be crucial as an anchor for reversible pyrrolopyrimidines; however, covalent inhibitor **9** is aligned by the covalent bond formed with the reactive cysteine to bind in a similar orientation.

Next, we focused on improving cellular activity, since none of the aforementioned inhibitors showed activity of less than 2 μM against the EGFR double mutant H1975 cells. Therefore, compounds **10b** and **10c**, bearing tertiary amines linked to the acrylamide, were synthesized according to the established route (Scheme S2†) in order to increase the solubility of these inhibitors. Indeed, **10b** exhibited an IC_50_ value of 26 nM in the biochemical assay and an EC_50_ value of 980 nM in cells against EGFR-L858R/T790M and improved 2-fold when compared to parent compound **10a**. Interestingly, compound **10c** bearing a piperidine moiety as a tertiary amine showed comparable potency as **10a** of about 2 μM in H1975 cells. We speculate that these amines make ionic interactions with Asp855 of the DFG motif (Fig. 1B), as observed with similar compounds described by Peng *et al.* (PDB ID: ; 4JRV).^58^ As a result, the acrylamide moiety would be directed away from Cys797 and this orientation would not allow for efficient covalent bond formation. This effect might be more pronounced for the more basic piperidine amine in compound **10c**.

Based on these observations, we designed compound **17a**, bearing a methyl piperazine moiety attached to the tolyl substituent extending to the solvent exposed lip of the ATP binding site (Fig. 1C). This compound was synthetically accessible from the common intermediate **15**, which under milder conditions could be carefully reduced to the amine **18** and transformed into the final compound **17a** as per the usual synthetic steps (Scheme S2†). Subsequent testing of this compound in the biochemical assay revealed inhibition of mutant EGFR in the single digit nanomolar range (with IC_50_ values of 2.3 and 4.0 nM against L858R and L858R/T790M, respectively) and selectivity over wild type EGFR (IC_50_ = 15 nM). Accordingly, we observed cellular potency of <14 nM and 137 nM against HCC827 and H1975 cells bearing mutated EGFR and 700 nM against the A431 wild type cell line (Table 1). Furthermore, the effect of **17a** on the phospho-levels of EGFR and downstream cascade proteins was analyzed using western blots. In good agreement with the results of the CellTiter-Glo assay, phosphorylation of EGFR, as well as downstream proteins, was reduced at concentrations of about 1 μM in A431 and around 100 nM in H1975 cells (Fig. 4).

**Fig. 3:**
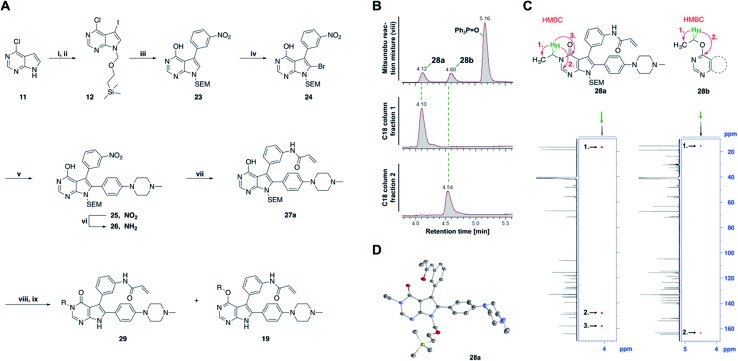
Mitsunobu reaction-based derivatization resulting in *O*-alkylated pyrrolopyrimidine and *N*-alkylated pyrrolopyrimidin-4-one EGFR inhibitors. (A) Synthesis of compounds **19** and **29**,^*a*^ (B) separation of SEM-protected compounds **28a** and **28b**, (C) HMBC NMR spectroscopic analysis reveals the structure of the separated constitutional isomers, (D) small-molecule crystal structure of compound **28a**, revealing the *N*-ethyl-substituted pyrrolopyrimidin-4-one structure (CCDC ID: ; 1876852). ^*a*^Reagents and conditions: (i) *N*-iodosuccinimide, DMF, rt, 97%; (ii) SEM-Cl, NaH, THF, 0 °C, 71%; (iii) *meta*-nitrobenzeneboronic acid, Pd(PPh_3_)_4_, K_2_CO_3_, MeCN : H_2_O (2 : 1), 150 °C, 90 min, μw, 74%; (iv) *N*-bromosuccinimide, MeCN, rt, quant.; (v) 4-(4-methylpiperazin-1-yl)phenylboronic acid pinacol ester, Pd(PPh_3_)_4_, K_2_CO_3_, DME : H_2_O (5 : 1), 150 °C, 90 min, μw, 68%; (vi) iron powder, NH_4_Cl, EtOH : H_2_O (4 : 1), reflux, 94%; (vii) acryloyl chloride, DIPEA, THF, 0 °C, 76%; (viii) ROH, DIAD, PPh_3_, THF, 40 °C, 30 min, sonication; (ix) TFA : CH_2_Cl_2_ (1 : 3), rt, then NaOH : THF (1 : 1), rt, 4–79% over two steps.

**Fig. 4:**
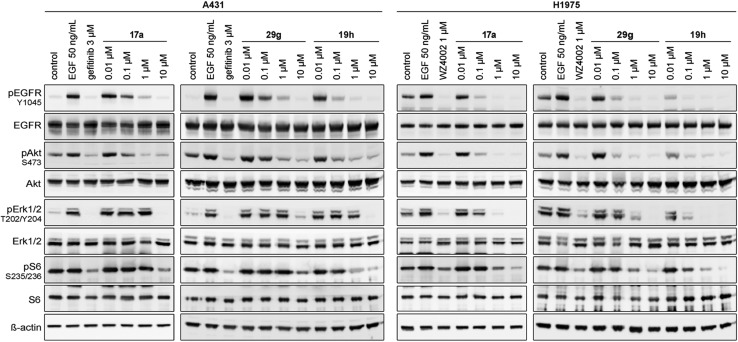
Western blot analysis of EGFR and downstream cascade phosphorylation inhibition by compound **17a**, **29g** and **19h** in A431 and H1975 cells.

Intrigued by these positive observations, compound **17a** was subjected to kinase selectivity profiling with 100 kinases. It was found to possess inhibitory activity of ≥70% at a screening concentration of 1 μM against 21 kinases and 8 mutant variants thereof. Among them were the nine kinases tested that bear a reactive cysteine in the position comparable to Cys797 in EGFR and could probably be covalently modified (Fig. S2 and Table S1†). To further assess the potential of the methyl piperazine moiety as a solubilizing group, compound **19a** was synthesized, in which the piperazine was directly linked to the 6-phenyl group without a methylene linker and was subsequently tested. Compound **19a** inhibited all variants of EGFR kinases tested with sub-nanomolar IC_50_ values and affected the viability of H1975 cells at an EC_50_ of 60 nM, while retaining selectivity over wild type. Again, the drop in activity of the reversible counterparts **17b** and **19b** indicated a covalent mode of action (Table 1).

Based on our structural analysis, we set out to determine whether interactions with the glycine-rich loop might be extended for beneficial reversible binding affinity. Therefore, glycol chains were introduced *ortho* to the acrylamide on the phenyl linker, giving compounds **20a** and **b**. The synthesis was possible by introducing 4-fluoro-3-nitrobenzene in a Suzuki–Miyaura coupling reaction with intermediate **13** and subsequent nucleophilic aromatic substitution with the respective alcohol in the presence of sodium hydride as key steps (Scheme S3†). Gratifyingly, biochemical characterization revealed inhibitory potency below the resolution limit of the HTRF assay (<0.1 nM) for these compounds. We assumed that additional interactions with the glycine-rich loop accounted for increased biochemical potency of **20a** and **b** as compared to **19a**. However, cells did not tolerate the glycol moiety well, as viability of the H1975 cell line was affected with an EC_50_ of about 300 nM for both compounds (Table 1). Interestingly, we noted a gain in activity against KRAS mutant cell lines A549 and H358 for **20a** (EC_50_ = 956 and 1101 nM) as compared to **20b** (EC_50_ = 5854 and 4474 nM), which might indicate off-target inhibition (Table S2†). In this series inhibitors **17a** and **19a** were identified as potent and selective inhibitors and their further development will be discussed in the following sections.

### Development of fast-forwarding Mitsunobu-based derivatization resulting in 3-substituted pyrrolopyrimidin-4-ones and 4-substituted pyrrolopyrimidines

The pyrrolopyrimidine-based inhibitors were optimized with respect to their covalent binding to Cys797 in EGFR, solubility, selectivity, and potency on the cellular level against drug resistant EGFR-L858R/T790M. Next, we set out to investigate substituents on the inhibitor molecules that would be located next to the methionine gatekeeper residue. We found that under harsh conditions utilized during the Suzuki–Miyaura coupling with compound **12** in a microwave reactor, compound **23** was formed bearing a hydroxyl group in the 4-position. According to the previously described synthetic scheme, compound **27** was synthesized and was then functionalized with ethanol as a substrate in a Mitsunobu reaction (Fig. 3A). As shown in Fig. 3B, LC/MS analysis revealed the formation of two products under the reaction conditions with different retention times in the LC, but with the same mass, indicating that two isomers had been formed. Separation of the isomers and structure determination by NMR spectroscopic analysis revealed the *N*3-substituted pyrimidin-4-one **28a** and *O*4-substituted pyrimidine **28b** have been formed, eluting in this order from a C18 column. The HMBC NMR experiment, which shows coupling of hydrogen to carbon atoms over two and three bonds, confirmed these structures (Fig. 3C and S3†). These experiments revealed that the CH_2_-hydrogens of the ethyl chain (highlighted in green) coupled with three carbons in the *N*-alkylated pyrimidin-4-one scaffolds (highlighted in red), in contrast to only two carbon atoms in *O*-alkylated pyrrolopyrimidines (highlighted in red). In addition, compound **28a** was crystallized and its small-molecule structure solved by means of X-ray diffraction analysis, as depicted in Fig. 3D (CCDC ID: ; 1876852).

### Biological testing of the second set of pyrrolopyrimidine EGFR inhibitors with optimized potency and selectivity

According to the established synthetic route, a set of *N*- and *O*-alkylated pyrrolopyrimidines was synthesized. The first subset of *N*-substituted pyrimidin-4-ones included compounds **29a–l**, among which **29a–f** were substituted with linear alkyl chains. Compounds with short chains, such as methyl or ethyl (**29a and b**), showed both potent inhibition of double mutant EGFR in the sub-nanomolar range and selectivity over wild type. In cellular assays, compounds **29a** and **b** affected the viability of H1975 cells at effective concentrations of below 200 nM. Compound **29c**, bearing a prop-1-yne substituent, revealed a 10-fold loss of activity in the biochemical setting, but showed comparable activity against the cell lines tested as compared to **29a** and **b** (Table 2). The EC_50_ values of 1390 and 2039 nM against A549 and H358 cell lines might hint at off-target inhibitory effects of **29c** (Table S2†), resulting in reduced viability of A431, HCC827, or H1975 cells. However, the crystal structure of **29b** was solved in complex with mutant cSrc and revealed the assumed binding mode (Fig. 2B and S1B;† PDB ID: ; 6HVF). The methyl piperazine moiety extended towards the solvent exposed end of the binding site and a covalent bond between the inhibitor and Cys345 was revealed by a clear electron density. In addition, the *N*-ethyl moiety pointed toward and is in close contact with the gatekeeper methionine side chain. This finding also explained the drop in inhibitory potency of compounds **29d–f** with longer alkyl chain substituents that exhibited a loss of selectivity for gatekeeper mutant EGFR and a potency of 1–20 nM in biochemical settings. The longer alkyl chains clashed with the methionine side chain, resulting in an unfavorable binding mode and loss of affinity. A similar trend was observed within compounds **29g** and **h** bearing branched alkyl chains and **29i–l** bearing cyclic substituents. The sterically demanding iso-butyl chain (**29h**), cyclopentyl (**29i**), cyclopropylmethyl (**29k**) or benzyl (**29l**) moieties were not well tolerated, resulting in IC_50_ values of more than 1 nM against L858R/T790M, while EGFR wild type was affected in the sub-nanomolar range. In contrast, the iso-propyl (**29g**) and cyclohexyl (**29j**) substituents seemed to possess reduced steric interference with the methionine (Fig. 1D) and resulted in efficient inhibition of cell viability at EC_50_ values of 65 and 77 nM, respectively (Table 2).

**Table 2:** Overview of the second set of *O*-alkylated pyrrolopyrimidine and *N*-alkylated pyrrolopyrimidin-4-one EGFR inhibitors and corresponding IC_50_ and EC_50_ determinations on different EGFR mutant variants and corresponding NSCLC cell lines^*a*^

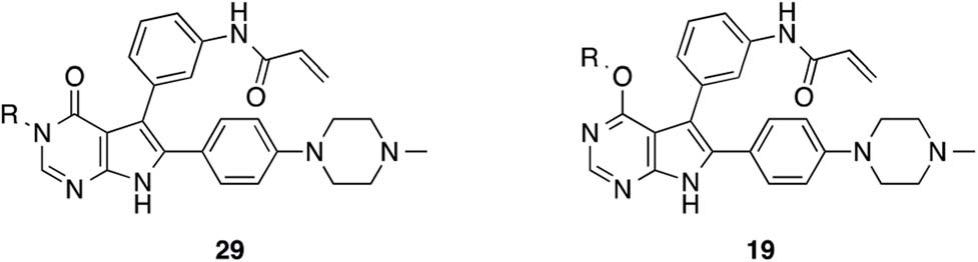							
Cpd	R	EGFR HTRF IC_50_ [nM]			EGFR CTG EC_50_ [nM]		
		wt	L858R	L858R/T790M	A431	HCC827	H1975
**29a**	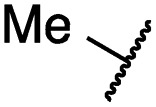	1.2 ± 0.1	0.5 ± 0.0	0.5 ± 0.0	1136 ± 350	82 ± 20	188 ± 64
**29b**	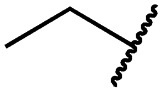	0.4 ± 0.2	0.2 ± 0.1	0.5 ± 0.0	739 ± 287	15 ± 1	144 ± 41
**29c**	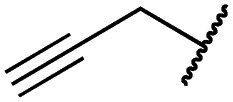	5.1 ± 0.1	4.0 ± 2.7	4.9 ± 1.3	1016 ± 335	22 ± 2	183 ± 48
**29d**	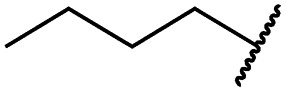	0.2 ± 0.0	0.3 ± 0.3	4.5 ± 0.8	767 ± 151	<14	550 ± 157
**29e**	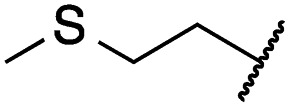	0.4 ± 0.1	0.1 ± 0.1	0.9 ± 0.1	1869 ± 595	<14	723 ± 188
**29f**	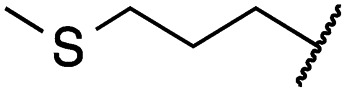	4.6 ± 1.1	1.7 ± 0.5	20 ± 6	15 244 ± 5940	134^*b*^	15 501 ± 3058
**29g**	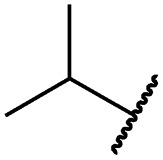	1.0 ± 0.1	0.4 ± 0.0	1.0 ± 0.3	1413 ± 917	67 ± 7	65 ± 11
**29h**	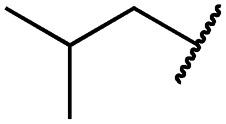	0.8 ± 0.2	0.2 ± 0.1	3.4 ± 1.4	1096 ± 506	45 ± 7	686 ± 199
**29i**	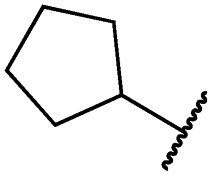	0.1 ± 0.0	<0.1	1.2 ± 0.1	502 ± 166	<14	263 ± 46
**29j**	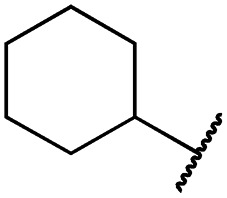	0.4 ± 0.1	0.2 ± 0.1	0.4 ± 0.1	1122 ± 656	<14	77 ± 15
**29k**	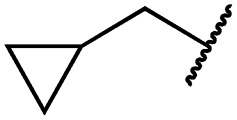	1.1 ± 0.8	0.9 ± 0.6	7.7 ± 3.9	453 ± 218	<14	228 ± 73
**29l**	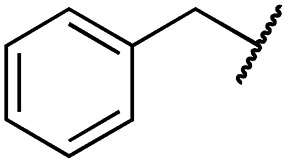	1.0 ± 0.2	1.5 ± 0.8	21 ± 1	1085 ± 169	44 ± 26	1573 ± 260
**19a**	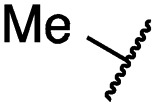	0.4 ± 0.0	0.1 ± 0.0	0.2 ± 0.0	638 ± 247	<14	60 ± 8
**19c**	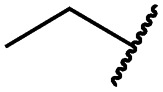	0.2 ± 0.1	<0.1	0.1 ± 0.1	759 ± 394	<14	64 ± 25
**19d**	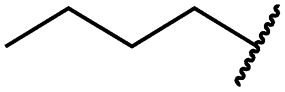	<0.1	<0.1	<0.1	1533 ± 273	<14	64 ± 8
**19e**	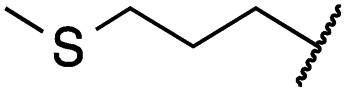	0.2 ± 0.0	<0.1	0.1 ± 0.1	3679 ± 500	<14	281 ± 88
**19f**	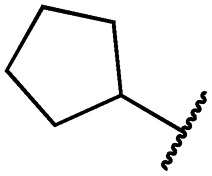	0.2 ± 0.0	<0.1	<0.1	1789 ± 744	n.d.	107 ± 45
**19g**	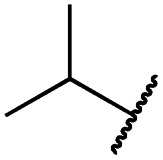	0.1 ± 0.0	<0.1	0.1 ± 0.0	334 ± 50	<14	38 ± 19
**19h**	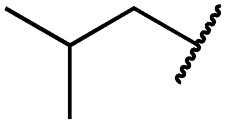	0.2 ± 0.2	<0.1	<0.1	1675 ± 402	<14	51 ± 19
**19i**	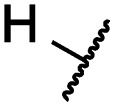	12 ± 4	12 ± 4	24 ± 12	23 781 ± 6389	557 ± 266	28 353 ± 2575

^*a*^Values are the mean ± SD of three independent measurements in duplicates.

^*b*^Single measurement; n.d. = not determined.

The effect of **29g** on the inhibition of EGFR autophosphorylation was also shown by means of western blot analysis to be around 100 nM in H1975 cells (Fig. 4). It is worth noting that testing the *N*-substituted compound series against KRAS mutant cell lines revealed EC_50_ values greater than 2 μM, which highlighted that these compounds showed no cytotoxicity or off-target effects. Only **29c** and **29k** might be two candidates that showed hints towards off-target inhibition (Table S2†).

The second subset of *O*-substituted pyrrolopyrimidines included compounds **19a–h**. In line with the finding that *N*-alkylated compounds (**29**) showed a tendency toward steric interference with the gatekeeper, *O*-alkylated compounds circumvented the steric conflict and revealed inhibition of all variants of EGFR below the assay resolution concentration of 0.1 nM. All compounds showed intense cellular potency with EC_50_ values of <14 nM in the HCC827 cell line harboring an activating EGFR mutation and, with the exception of compound **19e**, bearing a 3-(methylthio)propane substituent, they also showed intense cellular potency with EC_50_ values of below 100 nM in the drug resistant H1975 cell line. Outstanding potency was observed with compounds **19g** and **19h**, possessing the iso-propyl and iso-butyl ethers in the 4-position (Fig. 1E), showing EC_50_ values of 38 and 51 nM, respectively. Since **19h** revealed more pronounced selectivity over wild type as seen in A431, A549, and H358 cells (EC_50_ = 1.7 μM, 3.7 μM, and 3.7 μM, respectively) (Tables 2 and S2†), we further analyzed its biological impact on EGFR phosphorylation and downstream signaling intermediates. Western blot analysis revealed inhibition of pEGFR, pAkt, pErk1/2, and pS6 at concentrations between 10 and 100 nM in H1975 cells, while in A431 wild type bearing cells, the effect was observed only at higher concentrations between 100 nM and 1 μM (Fig. 4).

### Pharmacokinetic characterization of pyrrolopyrimidine EGFR inhibitors

To evaluate the pharmacokinetic characteristics, we set out to investigate the stability of a representative set of inhibitors in mouse and human liver microsomes (Tables S3, S4, and Chart S1†). It is interesting to note that *N*-alkylated compounds were slightly more stable as compared to *O*-alkylated pyrrolopyrimidines. They showed stability in mouse liver microsomes with an intrinsic clearance (CL_int_) between 4 and 84 μL min^–1^ mg^–1^ as compared to 46–128 μL min^–1^ mg^–1^. Carbocyclic moieties were not well-tolerated, independent of the *N*- or *O*-substitution site, and seemed to induce fast metabolic degradation of the inhibitor (Table S3 and Chart S1†). However, compounds **19g** and **h**, which possessed superior potency in cellular assays, revealed remarkable stability in mouse microsomes, both with clearance rates of 46 μL min^–1^ mg^–1^, and **19h** with a clearance of 6.7 μL min^–1^ mg^–1^ as determined in human liver microsomes. In addition, **19h** showed 100% stability in human and in mouse plasma, wherein it was bound to plasma proteins with 99.9 and 99.7%, respectively. Next, we investigated the cellular absorption in an artificial membrane permeability assay (PAMPA) and the Caco-2 cell assay, in which the ratio of the migration of test substances from the apical (A) to the basolateral (B) side and *vice versa* of a Caco-2 cell monolayer is determined to get an estimate of the oral bioavailability of test substances (influx: migration A → B > B → A; efflux: migration A → B < B → A). Compound **19h** displayed high penetration of the artificial membrane at pH 7.4 with 88% flux and only slight efflux in Caco-2 cells (ratio migration B → A : A → B = 3.1), which is in the range of 3^rd^ generation EGFR tyrosine kinase inhibitors (TKIs) (Table S4†).

**Fig. 5:**
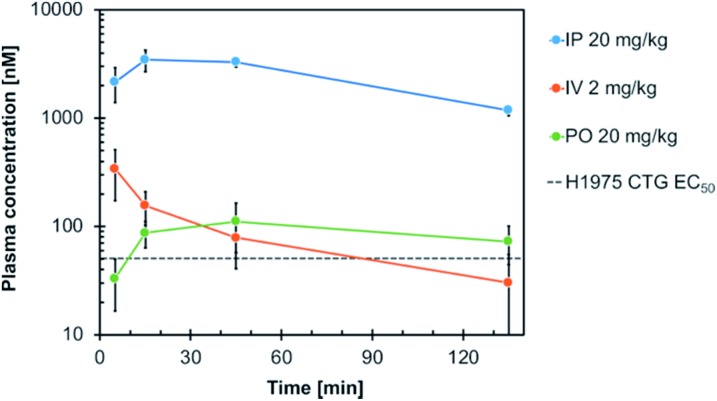
Plasma concentration–time profile of compound **19h** following intraperitoneal (IP), intravenous (IV) and oral (PO) administration in mice. Blood samples were taken at different time points up to 135 min post-dose and analyzed for the total plasma concentration by LC-MS/MS analysis. The *in vitro* EC_50_ of **19h** in H1975 cells (51 nM) is indicated.

Consequently, compound **19h** was evaluated with respect to its *in vivo* pharmacokinetic properties in mice. These studies revealed that the compound concentrations in the blood plasma were maintained above the *in vitro* EC_50_ concentration in H1975 cells for more than two hours after both, intraperitoneal injection (IP) and oral gavage (PO) (Fig. 5). The intraperitoneal route (dosed with 20 mg kg^–1^) resulted in relatively high blood concentrations with an AUC of 2867 h ng mL^–1^ (AUC_free_ = 8.6 h ng mL^–1^), a *t*_1/2_ of 1.2 h and a high *C*_max_ of 3.5 μM (*C*_max, free_ = 0.012 μM) (Table S5†). However, also an oral dose of 20 mg kg^–1^ resulted in blood concentrations above the EC_50_ concentration *in vitro*. These observations highlight the potential of the developed compounds for further *in vivo* studies in the future.

### Kinetic characterization and MS-based analysis of covalent bond formation

Next, we analyzed the covalent bond formation in detail which occurs in a two-step mechanism that requires initial reversible binding of the inhibitor to the binding site. Efficient covalent alkylation of the reactive cysteine can be achieved only after optimal spatial alignment of the reactive acrylamide in close proximity to the cysteine side chain.^59^ Since the analysis of X-ray co-crystal structures and biological evaluation suggested a more pronounced steric repulsion with the *N*-substituted compounds (**29**) as compared to *O*-alkylated pyrrolopyrimidines (**19**) (Fig. 1D, E and 2B), we investigated their binding characteristics in detail in a head-to-head comparison. Therefore, the *K*_i_ and *k*_inact_ parameters were determined, reflecting reversible contributions and the rate of covalent bond formation (Table 3). According to our previous findings, the *N*-alkylated compound **29g** showed poor affinity toward wild type and the L858R mutant EGFR, with *K*_i_ values of 10 nM and a drop in affinity when tested against gatekeeper mutant EGFR (*K*_i_ = 15 nM). In contrast, **19h** possessed affinities in the sub-nanomolar range with no loss against EGFR-L858R/T790M (*K*_i_ = 0.4 nM) and is, in this respect, superior over the 3^rd^ generation inhibitors osimertinib and rociletinib. We speculate that the alkyl ether in the 4-position can increase the electron density and thereby the hydrogen bond acceptor properties of the pyrrolopyrimidine *N*1 nitrogen, contributing to an elevated affinity.

**Table 3:** Overview of kinetic parameters *K*_i_, *k*_inact_, and *k*_inact_/*K*_i_ determined for **29g** and **19h** on different EGFR mutant variants^*a*^

Cpd	EGFR	*K*_i_ [nM]	*k*_inact_ [min^–1^]	*k*_inact_/*K*_i_ [μM^–1^ s^–1^]
**29g**	wt	10 ± 1	0.10 ± 0.03	0.16 ± 0.06
	L858R	10 ± 1	0.19 ± 0.08	0.31 ± 0.11
	L858R/T790M	15 ± 3	0.26 ± 0.09	0.30 ± 0.09
**19h**	wt	0.4 ± 0.1	0.05 ± 0.01	1.98 ± 0.22
	L858R	0.5 ± 0.1	0.28 ± 0.08	8.78 ± 0.65
	L858R/T790M	0.4 ± 0.1	0.17 ± 0.09	8.08 ± 0.77
Rociletinib	wt	74 ± 7	0.18 ± 0.01	0.04 ± 0.01
	L858R	1.8 ± 0.2	0.18 ± 0.05	1.67 ± 0.32
	L858R/T790M	1.7 ± 0.1	0.29 ± 0.05	2.95 ± 0.66
Osimertinib	wt	14 ± 2	0.43 ± 0.11	0.52 ± 0.05
	L858R	1.6 ± 0.3	0.30 ± 0.01	3.24 ± 0.46
	L858R/T790M	1.5 ± 0.1	0.33 ± 0.06	3.75 ± 0.39
EGF816	wt	25 ± 7.8	0.31 ± 0.06	0.23 ± 0.13
	L858R	10 ± 2.7	0.22 ± 0.02	0.38 ± 0.08
	L858R/T790M	7.7 ± 2.3	0.15 ± 0.04	0.38 ± 0.10

^*a*^Values are the mean ± SD of three independent measurements in duplicates.

In addition, ESI-MS based analysis of EGFR-T790M treated with several pyrrolopyrimidine inhibitors revealed a mass increase corresponding to the single-labeled receptor, as compared to a DMSO-treated control sample (Fig. S4A†). Moreover, tandem mass spectrometry indicated the specific single alkylation of Cys797 with compound **19h** (Fig. S4B†). These studies further confirmed the formation of a covalent adduct between these inhibitors and the gatekeeper mutant EGFR kinase domain.

### Potency of pyrrolopyrimidines and crystallographic studies on their binding mode in osimertinib resistant EGFR-T790M/C797S

As shown previously, the herein developed compounds bound covalently to Cys797 in EGFR, which is mutated to a serine in osimertinib refractory patients, thereby preventing covalent bond formation. However, we found that **19h** showed a more pronounced reversible binding character as compared to 3^rd^ generation TKIs and will therefore probably be less liable to demonstrate a loss in activity towards EGFR-L858R/T790M/C797S.^47^ Consequently, we tested the pyrrolopyrimidine compounds against the drug resistant triple mutant EGFR kinase (Table 4). As expected, the *O*-alkylated pyrrolopyrimidines possessed IC_50_ values of less than 50 nM, while *N*-alkylated inhibitors showed IC_50_ values of more than 110 nM. The only exception is *N*6-cyclohexyl bearing compound **29j** with an IC_50_ of 22 nM. *O*-Substituted inhibitors **19d**, **g** and **h** were the most potent among this series of compounds and showed high activities of around 9 nM in the biochemical assay. The herein developed compounds indeed showed superior biochemical potency as compared to 3^rd^ generation and quinazoline-based 1^st^ and 2^nd^ generation EGFR inhibitors.

**Table 4:** IC_50_ determination of pyrrolopyrimidine EGFR inhibitors against osimertinib resistant EGFR-L858R/T790M/C797S^*a*^

Compound	EGFR HTRF IC_50_ [nM]
	L858R/T790M/C797S
**29a**	354 ± 110
**29b**	406 ± 139
**29c**	110 ± 36
**29d**	803 ± 434
**29e**	768 ± 10
**29f**	>20 000
**29g**	555 ± 139
**29h**	356 ± 79
**29i**	249 ± 67
**29j**	22 ± 17
**29k**	243 ± 132
**29l**	2343 ± 952
**19a**	50 ± 19
**19b**	113 ± 47
**19c**	21 ± 7
**19d**	9.4 ± 1.5
**19e**	49 ± 35
**19f**	19 ± 11
**19g**	8.6 ± 3.2
**19h**	8.5 ± 3.7
**19i**	2061 ± 359
Gefitinib	250 ± 23
Afatinib	25 ± 17
WZ4002	452 ± 189
Rociletinib	541 ± 119
Osimertinib	116 ± 15
EGF816	398 ± 105
Staurosporine	1.5 ± 0.0

^*a*^Values are the mean ± SD of three independent measurements in duplicates.

**Fig. 6:**
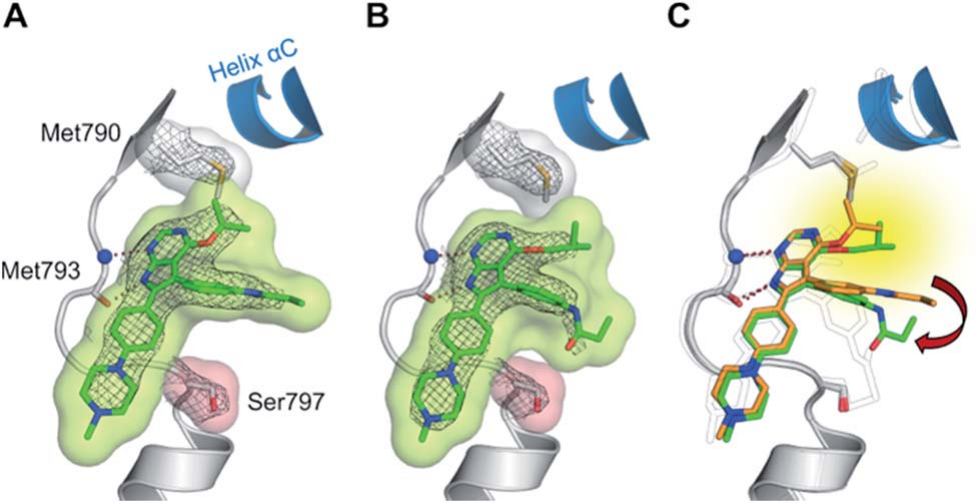
(A and B) Co-crystal structures of covalent pyrrolopyrimidines in complex with EGFR-T790M/C797S mutant. Diagrams of the experimental electron densities of (A) **19g**/EGFR-T790M/C797S at 2.7 Å (PDB ID: ; 6S89), (B) **19h**/EGFR-T790M/C797S at 2.6 Å resolution (PDB ID: ; 6S8A); 2Fo-Fc map contoured at an r.m.s.d. of 1. (C) Alignment of **19g** (orange) and **19h** (green) in complex with EGFR-T790M/C797S and **29b** in complex with cSrc-T338M/S345C (black outlined).

The high inhibitory potency of the developed compounds prompted us to perform crystallographic studies to gain insight into their binding mode. The two most potent compounds, **19g** and **19h**, could be successfully crystallized in complex with double mutant EGFR-T790M/C797S and the respective structures were solved and refined to resolutions of 2.7 Å (PDB ID: ; 6S89) and 2.6 Å (PDB ID: ; 6S8A), respectively. Therein, the pyrrolopyrimidine core resembles the binding mode previously observed in the cSrc model system (Fig. 6 and S1C and D†). The iso-propoxy- and iso-butoxy moieties form large interaction surfaces with the gatekeeper methionines (Fig. 6A and B), rather than steric interfering with the side chains as observed with pyrrolopyrimidin-4-ones (Fig. 2B and 6C). As previously anticipated, this might contribute to the high potency and reversible binding efficiency observed with these compounds. However, due to the C797S mutation, the acrylamide warhead adopts a position distant from the side chain and does not form any protein contact which might account for its high flexibility and only partial resolution covering this moiety. Interestingly, we observed an influence of the spatial size of the residue in 4-position on the orientation of the warhead and its linker (Fig. 6C). The sterically more demanding iso-butyl residue pushes the linker towards the mutated Ser797 side chain, which indicates that increasing the size of this substituent facilitates a ligand conformation that might more effectively target Cys797 in a non-C797S-mutant EGFR kinase.

To the best of our knowledge this is the first report of covalent EGFR inhibitors reversibly binding to the C797S mutant kinase domain. The structures provided insights into the contributions to the ligand's high reversible binding affinity and furthermore allowed us to derive features for an optimized covalent inhibitor. Both findings might stimulate further MedChem approaches in the future.

## Conclusions

The inevitable occurrence of acquired resistance to EGFR TKI treatment underlines the importance of the development of novel chemical entities that can be used to target drug resistant EGFR. Mutation of the gatekeeper residue T790M leads to resistance against the 1^st^ and 2^nd^ generation inhibitors and led to the development of 3^rd^ generation inhibitors that revealed their potency in the treatment of refractory NSCLC. Novel scaffolds, selectivity over wild type, and covalent alkylation of a reactive cysteine have been identified as crucial features that account for their clinical success.^27^ In order to employ novel scaffolds as mutant selective covalent inhibitors, we examined the pyrrolopyrimidine scaffold and developed a route for their synthesis. Optimization of solubility and cellular potency was performed in a series of 12 compounds. Further derivatization with the Mitsunobu reaction was found to yield a mixture of 3-substituted pyrrolopyrimidin-4-ones and 4-substituted pyrrolopyrimidines that could be easily separated, and which provided fast access to a second series of 19 EGFR inhibitors. These compounds were subjected to biochemical and cellular characterizations as well as western blot analysis and kinase selectivity profiling. Remarkable potency was observed with *N*3 iso-propyl substituted compound **29g** as well as compounds **19g** and **19h** possessing an iso-propyl and iso-butyl ether in the 4-position with EC_50_ values against drug resistant H1975 cells of 65, 38 and 51 nM, respectively. Compound **19h** revealed excellent selectivity as seen in A431, A549 and H358 wild type EGFR bearing cells and the pharmacological profile showed metabolic stability in liver microsomes and high plasma concentrations after administration to mice. By structure determination based on X-ray crystallography we gained insight into the difference in potency of *N*- and *O*-alkylated compounds, which indicated a more pronounced steric interference with the gatekeeper sidechain, observed with *N*-substituted inhibitors.

Despite the success of 3^rd^ generation EGFR inhibitors, the emergence of the C797S resistance affects their covalent binding to the kinase and results in loss of potency. In order to overcome this resistance mutation, an inhibitor with highly reversible binding affinity towards the binding pocket is needed as previously described by Engel and Becker *et al.*^47^ Luckily, we found that our covalent pyrrolopyrimidine inhibitors revealed a high degree of affinity and low dependence on covalent bond formation to achieve potency. Accordingly, a high potency against EGFR-L858R/T790M/C797S was found especially for the *O*4-substituted compounds, which were found to take advantage of favorable interactions with the methionine gatekeeper based on analysis of two obtained X-ray co-crystal structures in complex with C797S-mutated EGFR. Moreover, enhanced electron density of the scaffold ring system due to the ether substitution could probably strengthen the hydrogen bond formation properties with the hinge region.

Taken together, the observations in this study gave insight into the molecular binding characteristics of pyrrolopyrimidine-based EGFR inhibitors and these features must be considered in the development of efficient EGFR inhibitors. These findings highlight the requirements for future rational drug design projects, and we believe that they will stimulate additional research efforts in the field of medicinal chemistry.

## Author contributions

Conceptualization, J. L., M. P. M., J. E., C. S.-F., M. B., T. M., W. H., R. P. Z., J. G. H., C. S., W. A. L. v. O., S. B., D. R.; methodology, J. L., M. K., P. K., J. N., L. H., M. F., T. G., A. H.; validation, J. L., M. K., H. L. T., J. N., M. P. M., J. H., J. K., W. H., A. H., C. G., B. B.-L., L. K.; formal analysis, J. L., M. K., H. L. T., J. N., M. P. M., J. H., M. B., J. K., T. M., W. H., A. U., H. M., A. H., C. G., B. B.-L., L. K., R. P. Z.; investigation, J. L., M. K., P. K., H. L. T., J. N., L. H., M. F., J. H., T. G., M. B., J. K., W. H., G. G., A. U., H. M., A. H., C. G., L. K.; resources, C. S.-F.; data curation, J. L., M. K., H. L. T., M. P. M., M. B.; writing – original draft, J. L.; writing – review & editing, J. L., M. K., P. K., L. H., M. F., J. N., M. P. M., C. S.-F., M. B., T. M., W. H., J. G. H., W. A. L. v. O., S. B., D. R.; visualization, J. L., J. H., J. K., W. H., C. G., B. B.-L., L. K., R. P. Z.; supervision, J. L., M. P. M., J. E., C. S.-F., T. M., R. P. Z., J. G. H., C. S., S. B., D. R.; project administration, J. L., C. S.-F., W. H., R. P. Z., J. G. H., C. S., S. B., D. R.; funding acquisition, W. H., R. P. Z., J. G. H., C. S., W. A. L. v. O., S. B., D. R.

## Conflicts of interest

J. L. and C. S.-F. are shareholder and full-time employees of PearlRiver Bio GmbH. D. R. is shareholder and consultant of PearlRiver Bio GmbH. The Lead Discovery Center GmbH is shareholder of PearlRiver Bio GmbH. J. E., M. B. and A. U. are full-time employees of the Lead Discovery Center GmbH.

## Supplementary Material

Supplementary information

Crystal structure data
