# Supplementary material for: Inhibition of osimertinib-resistant epidermal growth factor receptor EGFR-T790M/C797S
Source: Chem Sci. 2019 Oct 4;10(46):10789–801. doi: 10.1039/c9sc03445e (PMC6886544; doi:10.1039/c9sc03445e)
Supplement: Supplementary file 1 [file SC-010-C9SC03445E-s001.pdf]

This journal is © The Royal Society of Chemistry 2019

SUPPORTING INFORMATION

Inhibition of Osimertinib-Resistant Epidermal Growth Factor Receptor EGFR-T790M/C797S

Jonas Lategahn,[a,b] Marina Keul,[a,b] Philip Klövekorn,[a] Hannah L. Tumbrink,[a] Janina Niggenaber,[a,b] Matthias P. Müller,[a,b] Luke Hodson,[a,c] Maren Flaßhoff,[a] Julia Hardick,[a,b] Tobias Grabe,[a,b] Julian Engel,[a] Carsten Schultz-Fademrecht,[d] Matthias Baumann,[d] Julia Ketzer,[e,f] Thomas Mühlenberg,[e,f] Wolf Hiller,[a] Georgia Günther,[g] Anke Unger,[d] Heiko Müller,[d] Alena Heimsoeth,[h,i] Christopher Golz,[a] Bernhard Blank-Landeshammer,[j] Laxmikanth Kollipara,[j] René P. Zahedi,[j] Carsten Strohmann,[a] Jan G. Hengstler,[g] Willem A.L. van Otterlo,[c] Sebastian Bauer,[e,f] and Daniel Rauh[a,b]*

[a] Faculty of Chemistry and Chemical Biology, TU Dortmund University, Otto-Hahn-Strasse 4a, 44227 Dortmund (Germany).

[b] Drug Discovery Hub Dortmund (DDHD) am Zentrum für Integrierte Wirkstoffforschung (ZIW), 44227 Dortmund (Germany).

[c] Department of Chemistry and Polymer Science, Stellenbosch University, Private Bag X1, Matieland 7602 (South Africa).

[d] Lead Discovery Center GmbH, Otto-Hahn-Strasse 15, 44227 Dortmund (Germany).

[e] Department of Medical Oncology, Sarcoma Center, West German Cancer Center, University Duisburg-Essen, Medical School, Hufelandstrasse 55, 45122 Essen (Germany).

[f] German Cancer Consortium (DKTK), 69120, Heidelberg (Germany).

[g] Leibniz Research Centre for Working Environment and Human Factors (IfADo), TU Dortmund University, Ardeystrasse 67, 44139 Dortmund (Germany).

[h] Molecular Pathology, Institute of Pathology, University Hospital of Cologne, Kerpener Strasse 62, 50937 Cologne (Germany).

[i] Department of Translational Genomics, Center of Integrated Oncology Cologne–Bonn, Medical Faculty, University of Cologne, Weyertal 115b, 50931 Cologne (Germany).

[j] Leibniz-Institut für Analytische Wissenschaften − ISAS − e.V., Otto-Hahn-Strasse 6b, 44227 Dortmund (Germany).

* Correspondence: Prof. Dr. Daniel Rauh, Phone: +49 (0)231/755-7080, eMail: daniel.rauh@tu-dortmund.de, Twitter: @DDHDortmund, Web: DDHDortmund.de.

TABLE OF CONTENTS

Scheme S1. Synthetic route for generation of compound **1** ______ S3

Scheme S2. Optimized synthetic route, yielding compound **10** and **17** ______ S4

Scheme S3. Synthetic key step for synthesis of compound **20** ______ S5

Figure S1. 2Fo-Fc maps and Fo-Fc stimulated annealing omit maps of complex crystal structures ______ S6

Figure S2 and **Table S1.** Kinase selectivity profiling of compound **17a** ______ S7

Figure S3. HMBC NMR analysis reveals the structure of the constitution isomers **28a** and **28b** ______ S8

Figure S4. Mass spectrometry-based analysis of covalent bond formation ______ S9

Table S2. Cellular evaluation of pyrrolopyrimidine EGFR inhibitors on KRAS-mutant cell lines ______ S10

Table S3 and **Chart S1.** Metabolic stability for selected pyrrolopyrimidine inhibitors in mouse liver microsomes ______ S11

Table S4. Pharmacokinetic parameters of **19h**, WZ4002 and osimertinib ______ S12

Table S5. Data collection and refinement statistics of complex crystal structures ______ S13

Table S6. Crystal data and structure refinement for **28a** ______ S14

Experimental Section ______ S15

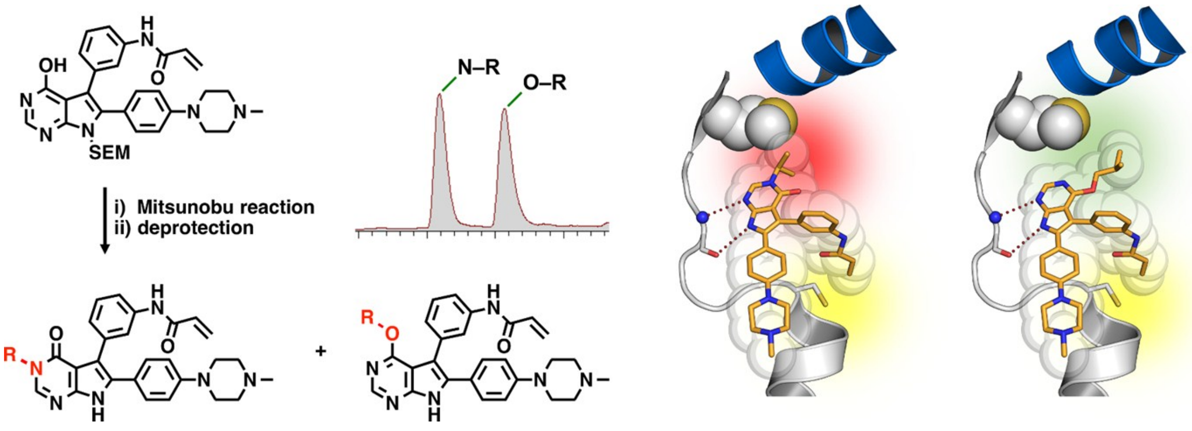

Scheme S1. Synthesis of pyrrolopyrimidine EGFR inhibitors. Initial synthetic route for generation of compound **1**.[a]

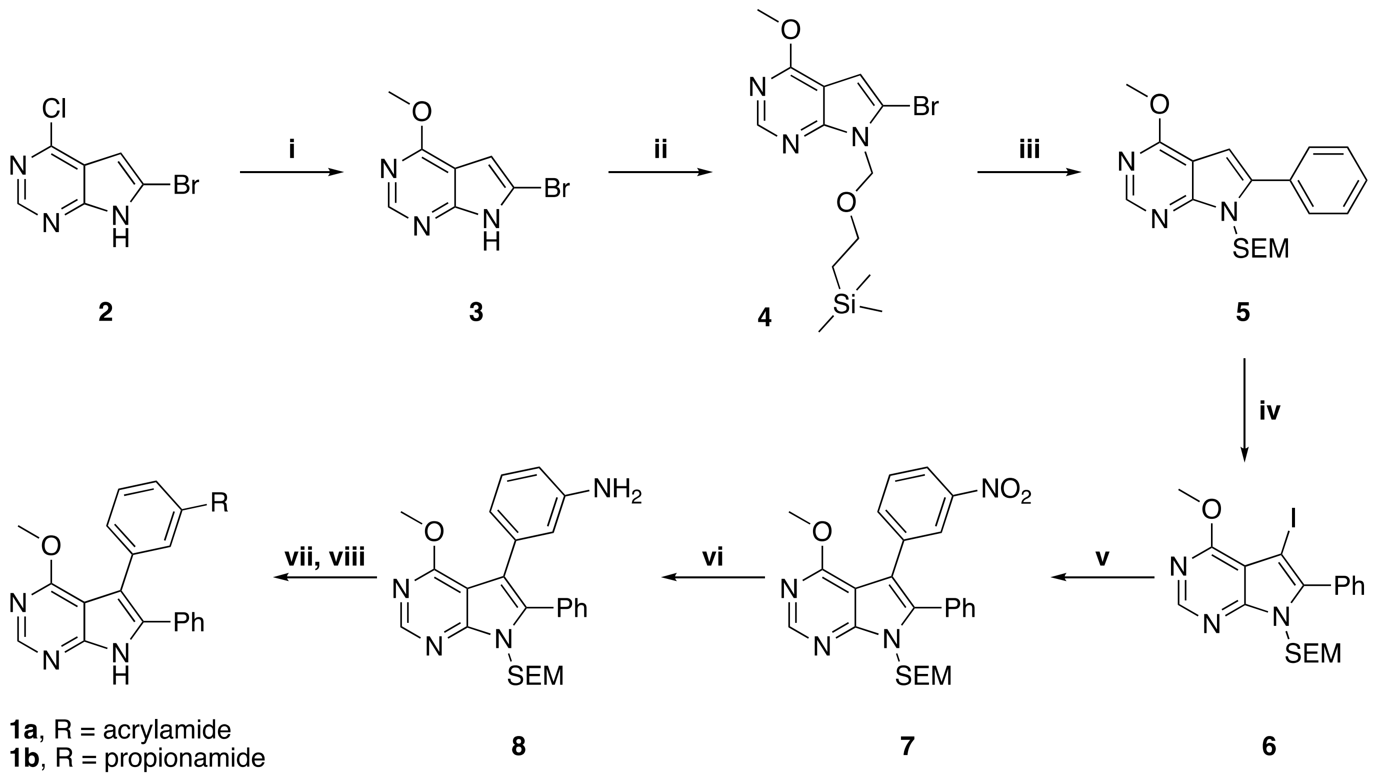

[a]Reagents and conditions: (i) NaOMe:MeOH (1:1.5), 70 °C, 87%; (ii) SEM-Cl, NaH, DMF, 0 °C, 67%; (iii) phenylboronic acid, $Pd(PPh_3)_4$, Na_2CO_3, DMF:H_2O (5:1), 80 °C, 84%; (iv) *N*-iodosuccinimide, MeCN, rt, 97%; (v) *meta*-nitrobenzeneboronic acid, $Pd(PPh_3)_4$, Na_2CO_3, DMF:H_2O (5:1), 80 °C, 89%; (vi) Pd/C, NH_4HCO_2, EtOH, reflux, quant.; (vii) acryloyl chloride or propionyl chloride, DIPEA, THF, 0 °C; (viii) TFA:CH_2Cl_2 (1:3), rt, 45-54% over two steps.

Scheme S2. Synthesis of pyrrolopyrimidine EGFR inhibitors. Optimized synthetic route, yielding compound **10** and **17**.[a]

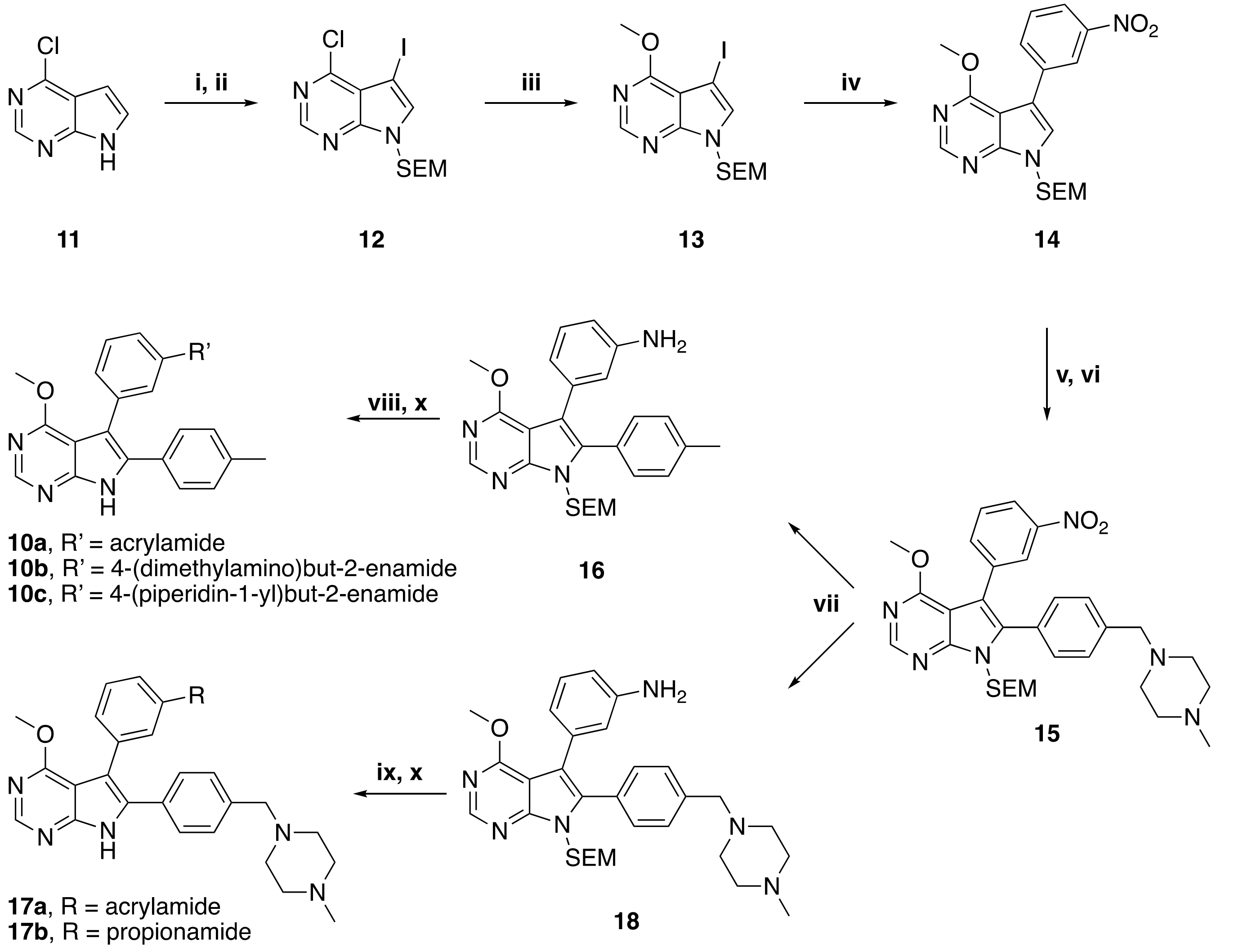

[a]Reagents and conditions: (i) *N*-iodosuccinimide, DMF, rt, 97%; (ii) SEM-Cl, NaH, THF, 0 °C, 79%; (iii) NaOMe:MeOH (1:1.5), 70 °C, 99%; (iv) *meta*-nitrobenzeneboronic acid, $Pd(PPh_3)_4$, K_2CO_3, $MeCN:H_2O$ (2:1), 70 °C, 55%; (v) *N*-bromosuccinimide, MeCN, rt, 85%; (vi) 4-[(4-methylpiperazin-1-yl)methyl]phenylboronic acid pinacol ester, $Pd(PPh_3)_4$, Na_2CO_3, dioxane:H_2O (5:1), 80 °C, 50%; (vii) Pd/C, NH_4HCO_2, EtOH, reflux, 74%; (viii) acryloyl chloride, DIPEA, THF, 0 °C or 4-(dimethylamino)crotonic acid or 4-(piperidin-1-yl)but-2-enoic acid hydrochloride, oxalyl chloride, DMF (cat.), THF:NMP (2:1), 0 °C; (ix) acryloyl chloride or propionyl chloride, DIPEA, THF, 0 °C; (x) TFA:CH_2Cl_2 (1:3), rt, 19-99% over two steps.

Scheme S3. Synthesis of pyrrolopyrimidine EGFR inhibitors. Synthetic key step for synthesis of compounds **20**.[a]

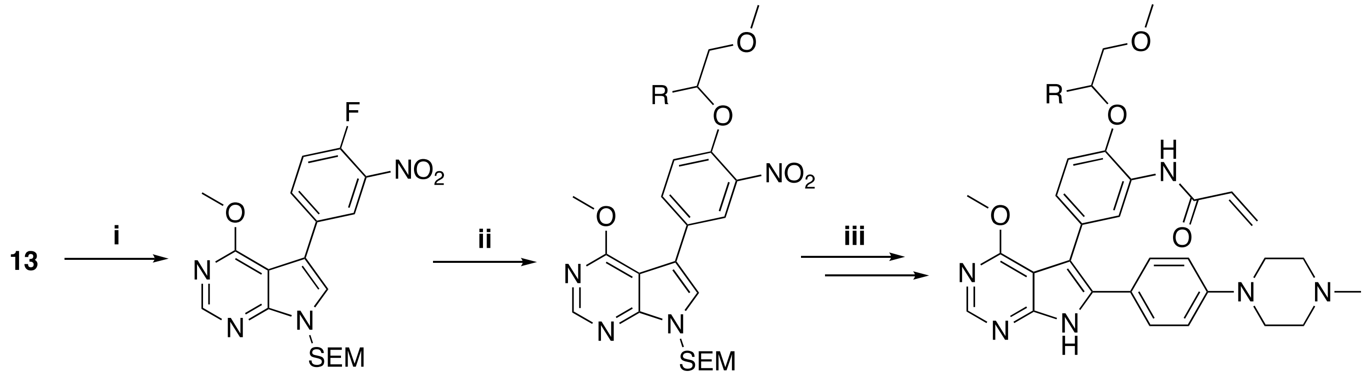

21 **22** **20a**, R = H **20b**, R = Me

[a]Reagents and conditions: (i) (4-fluoro-3-nitrophenyl)boronic acid, $Pd(PPh_3)_4$, Na_2CO_3, dioxane:H_2O (3:1), 80 °C, 86%; (ii) 2-methoxyethan-1-ol or 1-methoxypropan-2-ol, NaH, THF, 0 °C → rt, 71-73%; (iii) for steps see Scheme S2.

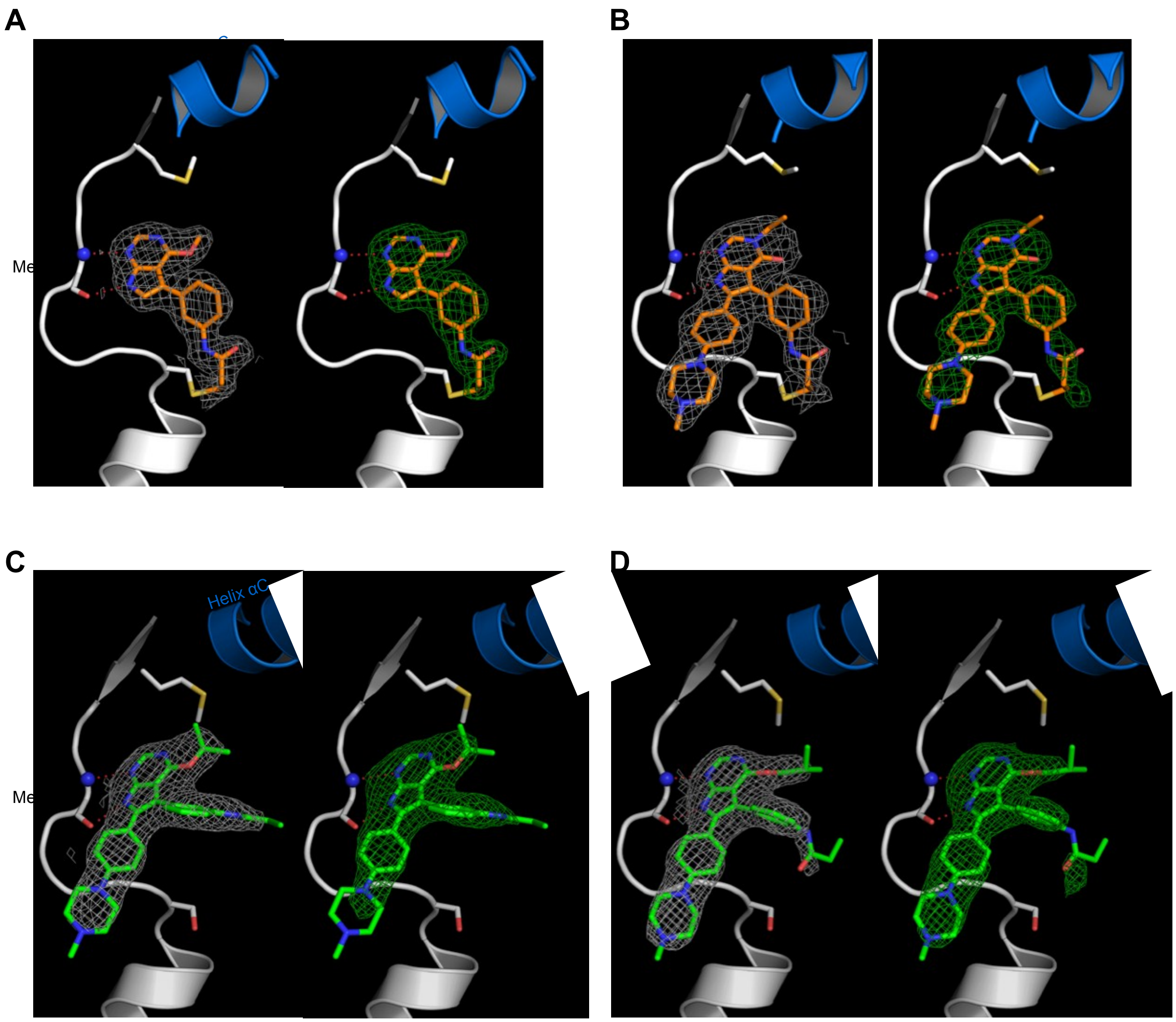

Figure S1. Electron density maps (2Fo-Fc) shown in grey (contoured at an r.m.s.d. of 1) and Fo-Fc stimulated annealing omit maps shown in green (contoured at an r.m.s.d. of 2.8), (A–B) of compounds **9** and **29b** in complex with cSrc-T338M/S345C and (C–D) of compounds **19g** and **19h** in complex with EGFR-T790M/C797S.

Kinase profiling of compound **17a** at a concentration of 1 µM generated with the SelectScreen® Profiling Service from Life Technologies.

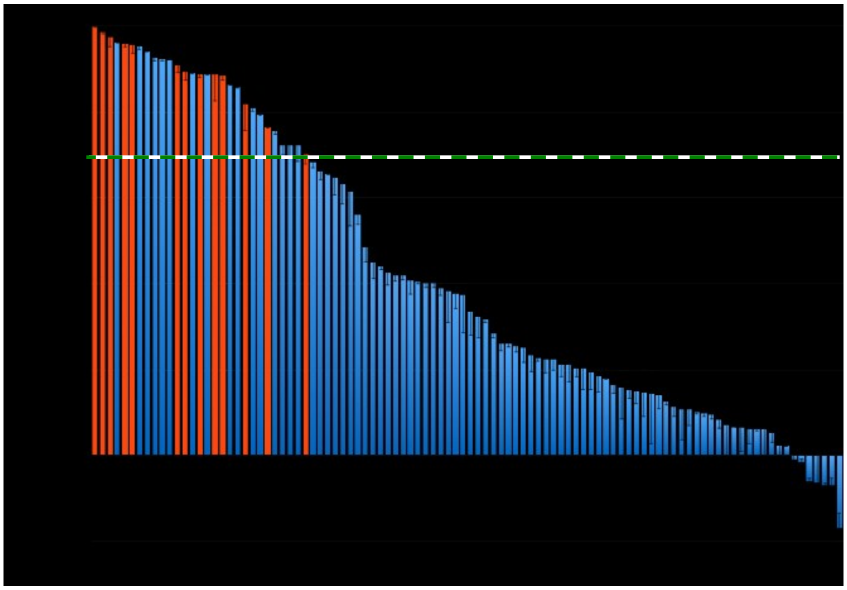

Figure S2. Kinase profiling of compound **17a**, Illustration as waterfall plot. 9 Kinases harboring a Cysteine isostructural to Cys797 in EGFR are highlighted in red (as indicated in Table S1).

Table S1. 21 kinases and 8 mutant variants show inhibition of ≥70% in a kinase profiling.[a]

Kinase	% inhibition @1 µM 17a	Kinase	% inhibition @1 µM 17a
ErbB4[b]	100 ± 1	ABL1-wt	89 ± 0
BLK[b]	99 ± 1	EGFR-L858R[b]	89 ± 6
EGFR-wt[b]	97 ± 2	EGFR-T790M/L858R[b]	88 ± 1
ABL1-G250E	96 ± 0	ABL1-E255K	86 ± 0
BTK[b]	96 ± 1	ABL2	86 ± 0
EGFR-L861Q[b]	96 ± 2	Her2[b]	82 ± 6
SRC-N1	95 ± 1	SRC	81 ± 1
ABL1-Y253F	94 ± 0	LCK	80 ± 0
YES1	93 ± 1	TXK[b]	77 ± 0
FGR	92 ± 1	FGFR2	76 ± 1
ROS1	92 ± 0	EPHA1	72 ± 2
JAK3[b]	91 ± 2	EPHB2	72 ± 3
EGFR-T790M[b]	89 ± 2	FGFR1	72 ± 4
ACVR1B	89 ± 0	ITK[b]	70 ± 2
BMX[b]	89 ± 1		

[a]Values are the mean ± SD of one measurement in duplicates; [b]Kinases harboring a Cysteine isostructural to Cys797 in EGFR.

A

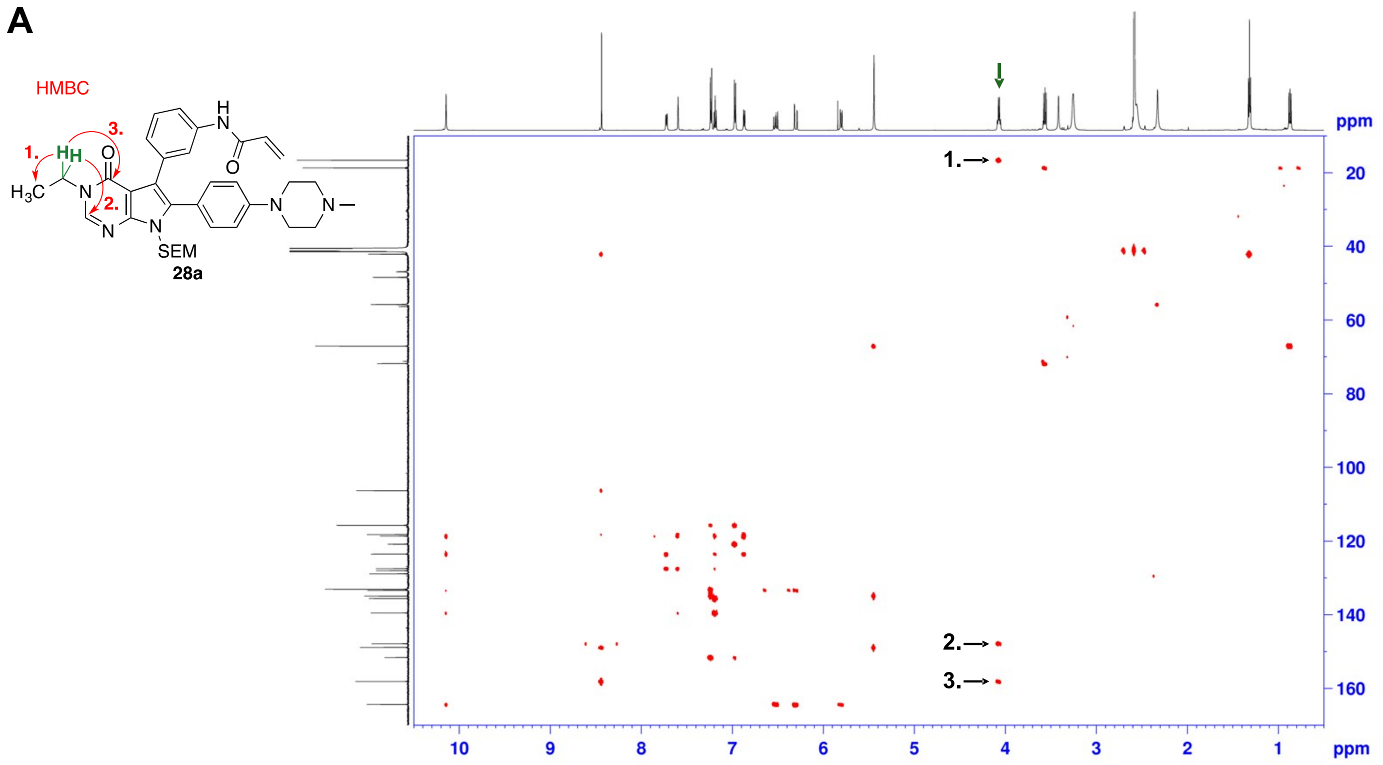

B

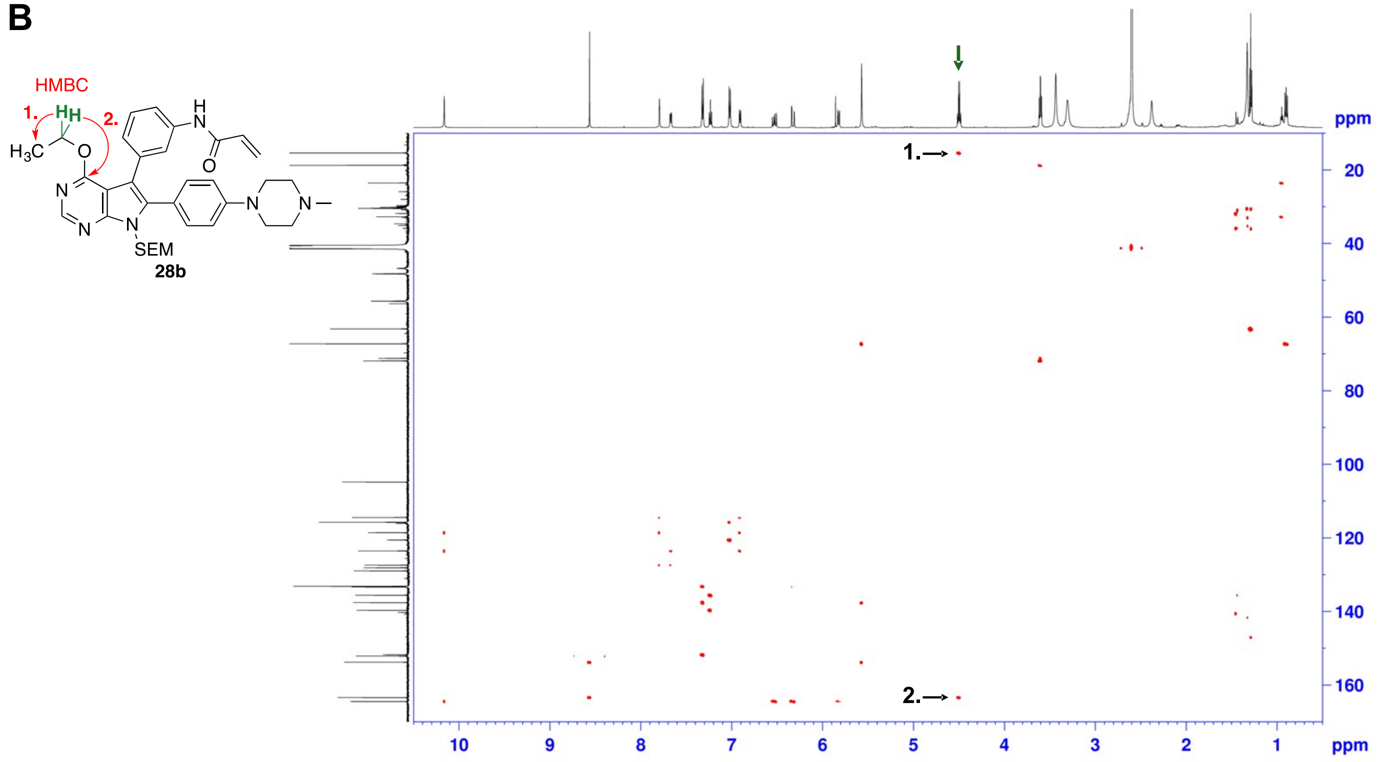

Figure S3. Mitsunobu reaction-based derivatization resulting in pyrrolopyrimidines and pyrrolopyrimidin-4-ones. HMBC NMR spectroscopic analysis revealed the structures of the separated constitutional isomers **28a** and **28b**.

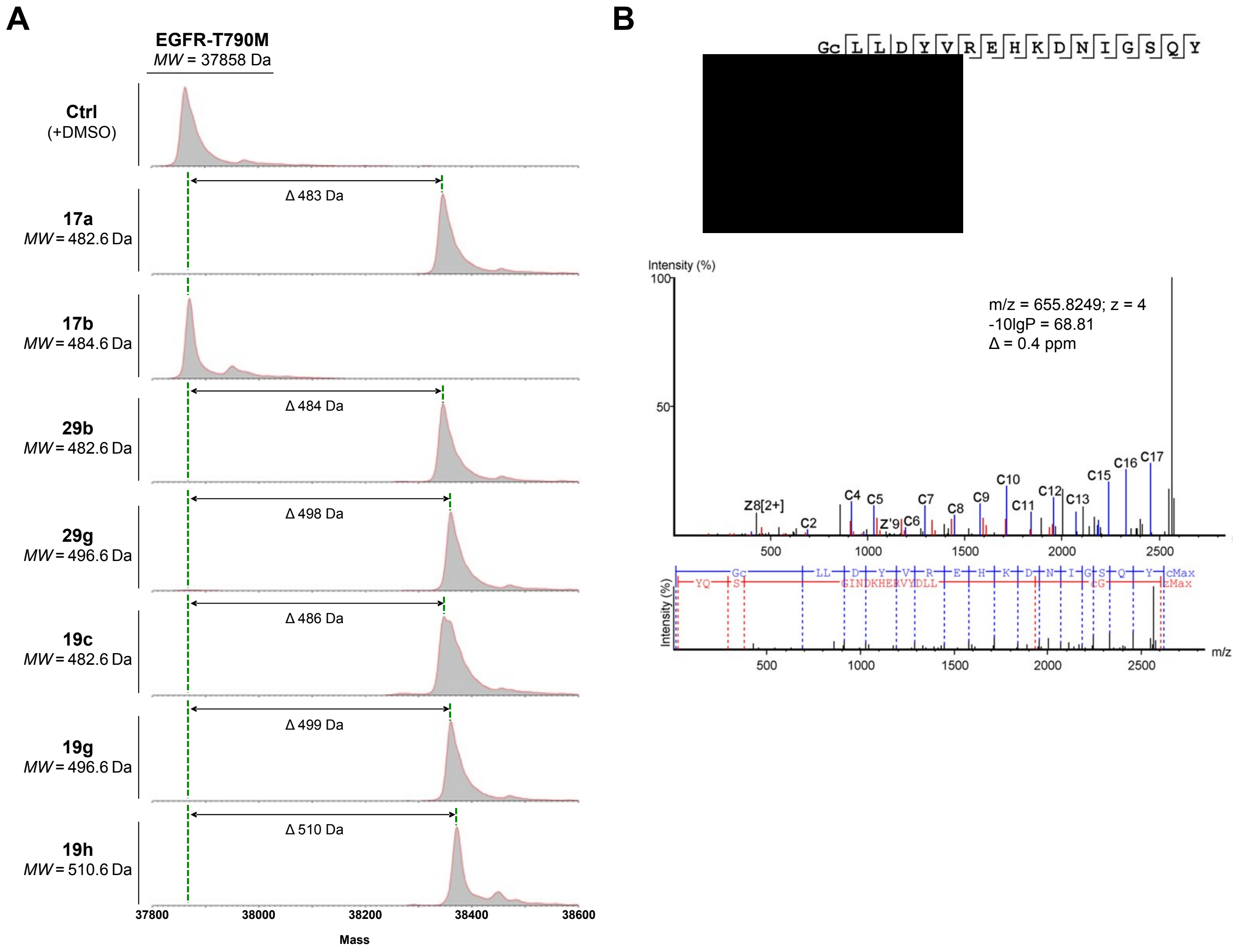

Figure S4. (A) Mass spectrometry-based analysis of covalent bond formation of EGFR-T790M with selected pyrimidine-based inhibitors, (B) Nano-LC-MS/MS analysis revealed Cys797 (within the peptide G**C**LLDYVREHKDNIGSQY) to be the site of covalent alkylation determined for compound **19h**.

Table S2. EC_{50} determinations on different KRAS mutant cell lines.[a]

Cpd	KRAS CTG EC_{50} [nM]		Cpd	KRAS CTG EC_{50} [nM]	
	A549	H358		A549	H358
**1a**	>30000	>30000	**29a**	8818 ± 697	9510 ± 403
**1b**	>30000	>30000	**29b**	4297 ± 1248	3466 ± 2009
**9**	13572 ± 1405	24458 ± 4866	**29c**	1390 ± 622	2039 ± 390
**10a**	10193 ± 504	14199 ± 678	**29d**	2970 ± 1066	3274 ± 693
**10b**	7055 ± 3227	10717 ± 3522	**29e**	22428 ± 11176	>30000
**10c**	4485 ± 896	4250 ± 336	**29f**	24658 ± 7554	11895[b]
**17a**	2495 ± 1629	2912 ± 1469	**29g**	5721 ± 240	9387 ± 3380
**17b**	5727 ± 1426	10333 ± 2166	**29h**	6138 ± 2116	6839 ± 866
**19a**	840 ± 289	1673 ± 721	**29i**	2129 ± 990	1938 ± 45
**19b**	6265 ± 1089	10243 ± 2239	**29j**	6052 ± 4596	6274 ± 1266
**20a**	956 ± 75	1101 ± 231	**29k**	1382 ± 985	1794 ± 520
**20b**	5854 ± 3657	4474 ± 3103	**29l**	25239 ± 8246	>30000
**gefi-tinib**	18721 ± 11924	10781 ± 4933	**19c**	1024 ± 46	1662 ± 759
**AEE 788**	3974 ± 720	3781 ± 383	**19d**	1132 ± 176	1643 ± 813
**afa-tinib**	1489 ± 430	1334 ± 515	**19e**	3010 ± 916	2573 ± 338
**WZ 4002**	3995 ± 545	4388 ± 512	**19f**	n.d.	n.d.
**osimer-tinib**	1827 ± 259	2835 ± 362	**19g**	1799 ± 131	2697 ± 758
			**19h**	3651 ± 1750	3692 ± 1460
			**19i**	>30000	>30000

[a]Values are the mean ± SD of three independent measurements in duplicates; [b]single measurement; n.d. = not determined.

Metabolic stability in mouse liver microsomes for selected pyrrolopyrimidine-based inhibitors.

Table S3. The *in vitro* intrinsic clearance CL_{int}, calculated based on the compound half-life $t_{1/2}$ (see Chart S1).

Cpd	Microsomal stability (phase I) CL_{int} mouse [µL min^{-1} mg^{-1}]
**1a**	156
**10a**	118
**10b**	26[b]
**17a**	26[b]
**20b**	8[a]
**29b**	4[a]
**29g**	8[a]
**29i**	84
**29j**	30[b]
**19d**	50[b]
**19f**	128
**19g**	46[b]
**19h**	46[b]
**afatinib**	6[a]
**WZ4002**	66
**osimertinib**	60

[a]low clearance; [b]medium clearance.

Chart S1. Relative compound degradation, grouped by (A) low, (B) medium, and (C) high clearance (as indicated in Table S3).

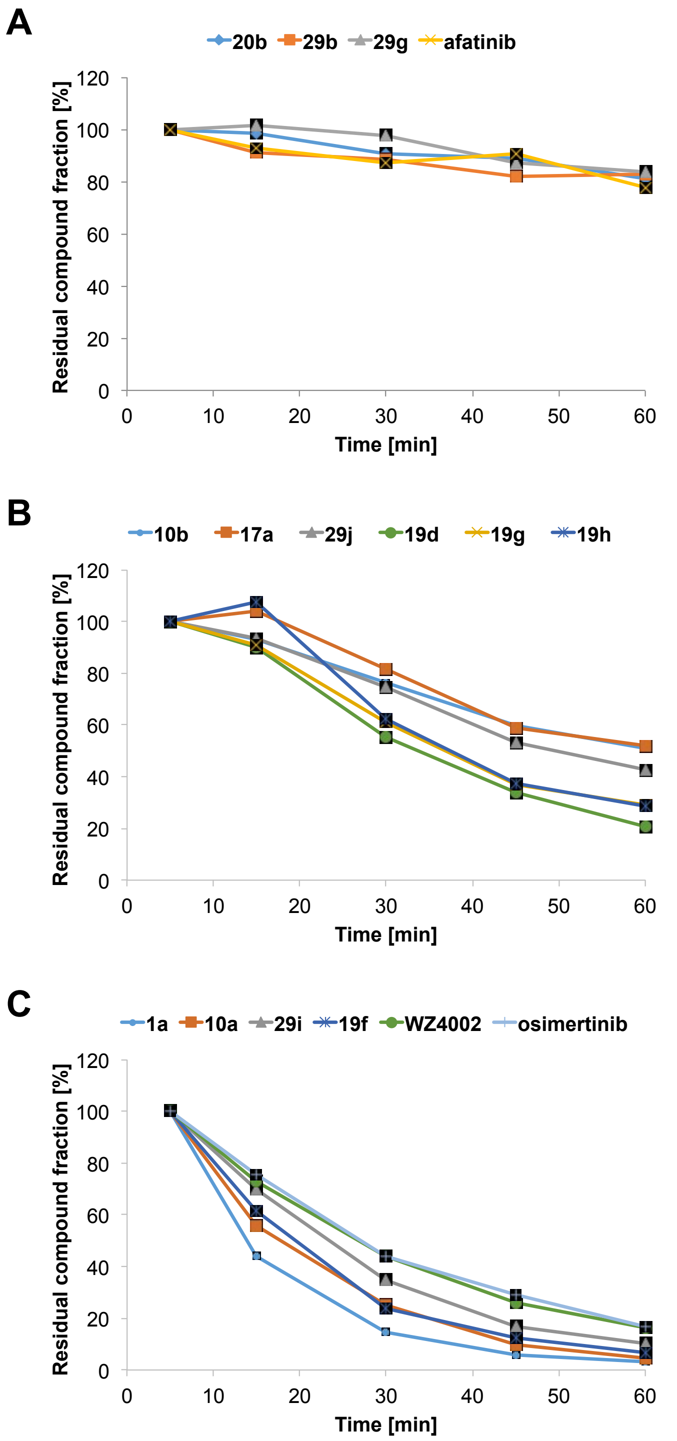

Table S4. Pharmacokinetic parameters of **19h**, WZ4002 and osimertinib.

**Pharmacokinetic parameter** (human / mouse)	**19h**	**WZ4002**	**osimertinib**
**Plasma stability** [%remain]	99 / 100	86 / 78	78 / 75
**Plasma protein binding** [%bound]	99.9 / 99.7	99.2 / 98.1	97.1 / 97.6
**Microsomal stability (phase I) CL_{int}** [$\mu L\ min^{-1}\ mg^{-1}$]	6.7 / 46	11 / 66	4.1 / 60
**PAMPA flux** [%]	88	n.d.	88
**Caco-2: P_{app} A→B** [10^{-6} cm s^{-1}]	1.2	4.9	5.0
**Caco-2: P_{app} B→A** [10^{-6} cm s^{-1}]	3.6	11.7	6.8
**Caco-2: ratio (B→A) : (A→B)**	3.1	2.4	1.4

n.d. = not determined.

Table S5. *In vivo* pharmacokinetic parameters of compound **19h** following intraperitoneal (IP), intravenous (IV) and oral (PO) administration in mice.

	**IV (2 mg kg^{-1})**		**IP (20 mg kg^{-1})**	**PO (20 mg kg^{-1})**
$t_{1/2}$ [h]	0.89	$t_{1/2}$ [h]	1.21	n.calc.
C_0 [ng mL^{-1}]	258	C_{max} [ng mL^{-1}]	1770	56
**AUC$_{0\text{-}t}$** [h ng mL^{-1}]	111	**AUC$_{0\text{-}t}$** [h ng mL^{-1}]	2867	101
**CL** [L h^{-1} kg^{-1}]	15	$F_{0\text{-}t}$ [%]	258	9
V_{ss} [L kg^{-1}]	16			

n.calc. = not calculated.

Table S6. Data collection and refinement statistics of complex crystal structures.[a]

	cSrc-T338M/S345C with **9** PDB ID: 6HVE	cSrc-T338M/S345C with **29b** PDB ID: 6HVF	EGFR-T790M/C797S with **19g** PDB ID: 6S89	EGFR-T790M/C797S with **19h** PDB ID: 6S8A
**Data collection**				
Space group	P1	P1	I 2 3	I 2 3
Cell dimensions				
a, b, c [Å]	42.1, 63.6, 74.5	42.1, 63.0, 74.5	143.8, 143.8, 143.8	142.0 142.0 142.0
α, β, γ [°]	101.4, 90.8, 91.1	100.2, 91.6, 90.0	90.0, 90.0, 90.0	90.0 90.0 90.0
Resolution [Å]	43.31–1.90 (2.00–1.90)	43.66–2.10 (2.20–2.10)	41.52-2.70 (2.80-2.70)	44.90-2.60 (2.70-2.60)
R_{meas}[%]	7 (62.6)	10.8 (69.3)	11.9 (208.9)	7.9 (180.2)
$I / \sigma I$	12.82 (2.72)	8.37 (2.1)	14.64 (1.17)	25.86 (2.04)
Completeness [%]	96.4 (95.2)	97.5 (97)	100.0 (99.9)	100.0 (100.0)
$CC_{1/2}$	99.8 (84.3)	99.6 (81.2)	99.9 (47.5)	100.0 (73.8)
Redundancy	3.5 (3.5)	3.5 (3.6)	10.01 (9.59)	19.95 (21.13)
Refinement				
Resolution [Å]	43.31–1.90	43.66–2.10	41.52-2.70	44.90-2.60
No. reflections	57563	42926	13756	14819
R_{work} / R_{free}	17.78/19.92 (25.32/29.44)	20.85/24.31 (29.37/34.14)	18.82/23.37 (35.06/41.38)	18.55/20.74 (31.47/40.90)
No. atoms				
Protein	4127	3902	2395	2425
Ligand/ion	44	84	49	50
Water	321	173	0	2
B-factors				
Protein	37.51	50.21	79.32	83.45
Ligand/ion	30.18	54.38	97.64	115.64
Water	39.39	46.56	0	81.73
R.m.s. deviations				
Bond lengths [Å]	0.011	0.006	0.007	0.004
Bond angles [°]	1.075	0.817	0.989	0.745

[a]Diffraction data from a single crystal was used to determine the complex structure. Values in parenthesis are referring to the highest resolution shell.

Table S7. Crystal data and structure refinement for **28a** (CCDC ID: 1876852).

Empirical formula	$C_{34}H_{44}N_6O_3Si$
Formula weight	612.84
Temperature [K]	100.03
Crystal system	triclinic
Space group	P-1
a, b, c [Å]	10.380(2), 12.351(3), 13.352(3)
α, β, γ [°]	98.84(3), 91.06(3), 106.73(3)
Volume [Å^3]	1616.3(6)
Z	2
ρ_{calc} [g/cm^3]	1.259
μ [mm^{-1}]	0.117
F(000)	656.0
Crystal size [mm^3]	0.219 × 0.176 × 0.050
Radiation	MoKα (λ = 0.71073)
2Θ range for data collection [°]	5.036 to 57.668
Index ranges	-14 ≤ h ≤ 14, -16 ≤ k ≤ 16, -18 ≤ l ≤ 18
Reflections collected	71228
Independent reflections	8384 [R_{int} = 0.0863, R_{sigma} = 0.0587]
Data/restraints/parameters	8384/0/460
Goodness-of-fit on F^2	1.028
Final R indexes (I≥2σ (I))	R_1 = 0.0636, wR_2 = 0.1444
Final R indexes (all data)	R_1 = 0.1070, wR_2 = 0.1678
Largest diff. peak/hole / e [Å^{-3}]	0.95/-0.55

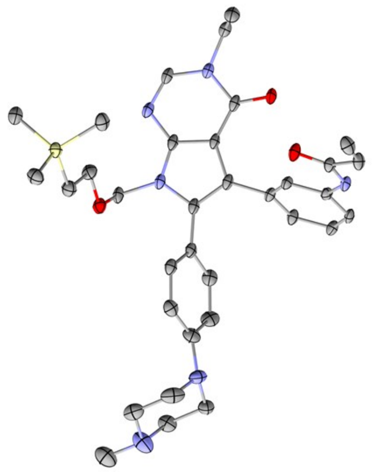

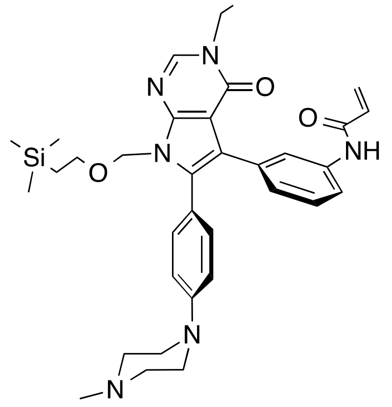

28a

EXPERIMENTAL SECTION

Structural analysis

Structures of the anticipated binding modes of the inhibitors were generated by alignment of a co-crystal structure of EGFR-T790M complexed with the pyrrolopyrimidine-based EGFR inhibitor AEE788 (Figures 1A,C-E; PDB ID: 2JIU) or EGFR-wt complexed with a similarly binding furo[2,3-d]pyrimidine inhibitor (Figure 1B; PDB ID: 4JRV) to T790M-mutated apo EGFR (PDB ID: 3UG1) using PyMOL, (The PyMOL Molecular Graphics System, Version 2.2.0 Schrödinger, LLC). Following, the chemical structure of the respective ligand was adjusted, and energy minimized while maintaining the hinge-binding scaffold using Accelrys Discovery Studio Client (Dassault Systèmes BIOVIA, Discovery Studio Client, Version 3.1, San Diego: Dassault Systèmes). PyMOL was used for generating the figures.

Construct design and protein expression and purification of of EGFR-mutants.

For the EGFR-L858R/T790M/C797S assay construct the DNA encoding residues compromising the juxtamembrane segment, the kinase domain and the C-terminal tail of human EGFR (UniProt entry P00533, residues 695-1210) were synthesized by *GeneArt* (life technologies). The construct was cloned into pIEX/Bac5 expression vector (MerckMillipore) including an GST-tag, using KpnI and Bsu36I restriction sites. Point mutations L858R, T790M and C797S were introduced by site-directed mutagensis (QuikChange, Stratagene/Agilent Technologies). Transfection, virus generation, and amplification were carried out in *Spodoptera frugiperda* cell line *Sf9* following the BacMagic protocol. EGFR-T790M and EGFR-L858R/T790M/C797S were expressed in *Sf9* cells using the *BacMagic* system and purified as follows. After three days of expression (27 °C, 110 rpm) the cells were harvested (300 g, 20 min), resuspended in buffer A (50 mM Tris, 500 mM NaCl, 5% glycerol, 1 mM DTT, pH 7.5) and homogenized by french press. The lysate was cleared

by centrifugation at 40.000 g for 1 h at 8 °C and loaded on a pre-packed GSTrap 4B Column (GE Healthcare). The elution was done with buffer B (50 mM Tris, 500 mM NaCl, 5% glycerol, 1 mM DTT, pH 7.5, 10 mM glutathione). Afterwards the fractions containing the assay construct EGFR-L858R/T790M/C797S were concentrated and applied to a HiLoad 16/600 superdex 75 pg column (GE Healthcare) in buffer C (25 mM TRIS, 250 mM NaCl, 10% glycerol, pH 8). The purified protein was concentrated to 5 mg/mL and stored at -80 °C until further use. Protein identity was confirmed by ESI-MS analysis.

For crystallographic studies, codon optimized DNA encoding residues 696-1022 of the human EGFR with the mutations T790M, C797S, E865A, E866A and K867A with a N-terminal His_6-tag and thrombin cleavage site was cloned into a pIEX/Bac-3 vector and expressed in *Sf9* insect cells. Frozen cells of the expression were resuspended in the lysis buffer containing of 600 mM NaCl, 50 mM Tris-HCl pH 7.5, 15% glycerol and 1 mM TCEP supplemented with a protease-inhibitor cocktail (Complete EDTA-free), homogenized, lysed and centrifuged at 20500 rpm for 1 h at 4 °C. The filtered supernatant was loaded onto a nickel-affinity column. The protein was eluted with 500 mM NaCl, 25 mM Tris-HCl pH 8, 250 mM imidazole, 10% gylcerol and 1 mM TCEP followed by a cleavage with thrombin to remove the His_6-tag and a second nickel-affinity chromatography capturing the flow through. Finally, the protein was purified by size-exclusion chromatography (100 mM NaCl, 25 mM Tris-HCl pH 8, 10% glycerol and 1 mM TCEP and concentrated to 6.5 mg/mL. Protein identity was confirmed by ESI-MS analysis.

Activity-based assay for IC_{50} determination and kinetic characterization.

IC_{50} determinations for EGFR and its mutants (Carna Biosciences, lot13CBS-0005K for EGFR-wt; Carna, lot13CBS-0537B for EGFR-L858R and Carna, lot12CBS-0765B for EGFR-L858R/T790M) were performed with the HTRF KinEASE-TK assay from Cisbio according to the manufacturer's

instructions. Briefly, the amount of EGFR in each reaction well was set to 0.60 ng EGFR-wt (0.67 nM), 0.10 ng EGFR-L858R (0.11 nM), 0.07 ng EGFR-T790M/L858R (0.08 nM) or 0.80 ng EGFR-L858R/T790M/C797S (0.88 ng), respectively. An artificial substrate peptide (TK-substrate from Cisbio) was phosphorylated by EGFR. After completion of the reaction (reaction times: 25 min for wt, 15 min for L858R, 20 min for T790M/L858R and 10 min for L858R/T790M/C797S), the reaction was stopped by addition of buffer containing EDTA as well as, an anti-phosphotyrosine antibody labeled with europium cryptate and streptavidin labeled with the fluorophore XL665. FRET between europium cryptate and XL665 was measured after an additional hour of incubation to quantify the phosphorylation of the substrate peptide. ATP concentrations were set at their respective K_m-values (9.5 μM for wt, 9 μM for L858R, 4 μM for L858R/T790M and 11 µM for L858R/T790M/C797S) while a substrate concentration of 1 μM, 225 nM, 200 nM and 325 nM, respectively, was used. Kinase and inhibitor were preincubated for 30 min before the reaction was started by addition of ATP and substrate peptide. An EnVision multimode plate reader (Perkin Elmer) was used to measure the fluorescence of the samples at 620 nm (Eu^{3+}-labeled antibody) and 665 nm (XL665 labeled streptavidin) 50 μs after excitation at 320 nm. The quotient of both intensities for reactions made with eight different inhibitor concentrations was then analyzed using the Quattro Software Suite for IC_{50}-determination. Each reaction was performed in duplicate, and at least three independent determinations of each IC_{50} were made.

For kinetic characterization (k_{inact}/K_i) the inhibitors were incubated with EGFR-wt, -L858R or -L858R/T790M, respectively, over different periods of time (2-90 min) whereas durations of enzymatic and stop reactions were kept constant as stated above. A six-fold dilution series (eight data points per IC_{50} curve) starting at 20 μM final compound concentrations was applied. Compound dilutions were generated using the acoustic dispensing system “ECHO 520 Liquid

Handler" from Labcyte (Sunnyvale, California, USA) and the according dose-response software "Echo Dose-Response v1.5.4". Calculated IC_{50} values were plotted versus incubation time and data was fit as described in the literature to determine k_{inact} and K_i (B.F. Krippendorff, R. Neuhaus, P. Lienau, A. Reichel, W. Huisinga, *J. Biomol. Screen* **2009**, *14*, 913-923).

Crystallization of cSrc-T338M/S345C with 9 and 29b.

The inhibitors **9** and **29b** were co-crystallized with cSrc-T338M/S345C by incubating 5 mg/mL protein with a threefold molar excess of inhibitor (10 mM DMSO stock) for 1 h at 4 °C to allow enzyme–inhibitor complex formation prior to crystallization. Crystals were grown using the hanging drop method at 20 °C after mixing 1 µL protein-inhibitor solution with 1 µL reservoir solution (10% ethylene glycol, 1 mM sodium chloride, pH 7.0). The data sets were collected at the PXII X10SA beamline of the Swiss Light Source (PSI, Villingen, Switzerland). All data sets were processed with XDS and scaled using XSCALE (W. Kabsch, *J Appl Crystallogr* **1993**, *26*, 795-800).

Crystallization of EGFR-T790M/C797S with 19g and 19h.

The concentrated protein EGFR-T790M/C797S (5.5 or 5.3 mg/mL) was incubated with a three-fold excess of inhibitors **19g** and **19h** (10 mM DMSO stock) for 1 h on ice. Crystals were grown at 20 °C by hanging drop vapor diffusion method. The protein–compound-complex was mixed in a 1:1 ratio (1 µL Protein : 1 µL reservoir solution containing 1.35 M K-Na-tartrate, 100 mM Na-MES pH 6.5, 2% 1,3-Propanediol or 1.15 M K-Na-tartrate, 100 mM Na-MES pH 7.0), respectively. Crystals were grown after one day and were flash-frozen in liquid nitrogen. Diffraction data were recorded using the PXII X10SA beamline at the Swiss Light Source (PSI, Villingen, Switzerland). The data were processed and merged using XDS and scaled using XSCALE.

Structure determination and refinement.

The complex crystal structures of cSrc-T338M/S345C in complex with compounds **9** and **29b** and EGFR-T790M/C797S in complex with **19g** and **19h** were solved by molecular replacement with PHASER (R.J. Read, *Acta Crystallogr D Biol Crystallogr* **2001**, *57*, 1373-1382) using structure PDB ID: 3G5D and 5J9Z as templates. The molecules in the asymmetric unit were manually adjusted using the program COOT (P. Emsley, K. Cowtan, *Acta Crystallogr D Biol Crystallogr* **2004**, *60*, 2126-2132). The refinement was performed with Phenix 1.10 (P.D. Adams, P.V. Afonine, G. Bunkoczi, V.B. Chen, I.W. Davis, N. Echols, J.J. Headd, L.W. Hung, G.J. Kapral, R.W. Grosse-Kunstleve, A.J. McCoy, N.W. Moriarty, R. Oeffner, R.J. Read, D.C. Richardson, J.S. Richardson, T.C. Terwilliger, P.H. Zwart, *Acta Cryst* **2010**, *D66*, 213-221). Inhibitor topology files were generated using the Dundee PRODRG2 server (R.A. Laskowski, M.W. Macarthur, D.S. Moss, J.M. Thornton, *J Appl Crystallogr* **1993**, *26*, 283-291). Refined structures were validated with PROCHECK (R.A. Laskowski, M.W. Macarthur, D.S. Moss, J.M. Thornton, *J Appl Crystallogr* **1993**, *26*, 283-291) and the PDB validation server. Data collection, structure refinement statistics, PDB ID codes, further details for data collection are provided in Table S7. PyMOL (W.L. DeLano, *The PyMOL Molecular Graphics System*) was used for generating the figures.

Cell culture.

A431, HCC827, H1975, A549 and H358 cells were obtained from the American Type Culture Collection (ATCC). A431 cells were cultured in DMEM high glucose media (Life Technologies, Germany), and HCC827, H1975, A549 as well as H358 cells were cultured in RPMI media (Life Technologies, Germany), both containing L-Glutamine and were supplemented with 10% FBS (GE Healthcare, Austria) and 1% PenStrep (Life Technologies, Germany) in a humidified incubator at 37 °C and 5% CO_2.

Cell line authentication has been performed last in August 2017 by STR profiling of 16 alleles.

Viability assay.

Cells were seeded at cell numbers that assure linearity and optimal signal intensity (150-300 cells/well, 25 µL) and cultured for 24 hours in serum- and antibiotics-containing media in humidified chambers at 37 °C / 5% CO_2. The cells were then treated with EGFR inhibitors in serial dilutions (14 nM to 30 µM) with DMSO and Staurosporine as control and incubated for 96 hours. Afterwards viability studies were carried out using CellTiter-Glo Assay (Promega, USA) that is a homogeneous method of determining the number of viable cells in culture. It is based on quantification of ATP, indicating the presence of metabolically active cells. For these studies, CellTiter-Glo reagent was prepared according to the instructions of the kit and diluted 1:1 with the complete growth medium suitable for the corresponding cell line. Thereon, reagent and assay plates were equilibrated at room temperature for 20 min. Equal volumes of the reagent were added to the volume of culture medium present in each well (25 µL). The plates were mixed for 2 minutes on an orbital shaker to induce cell lysis. The microplates were then incubated at room temperature for 20 minutes for stabilization of the luminescent signal. Following incubation the luminescence was recorded on an EnVision microplate reader (Perkin Elmer) using 500 ms integration time. The data was then analyzed using the Quattro Research Software Suite for EC_{50}-determination. As quality control the Z'-factor was calculated from 16 positive and negative control values. Only assay results showing a Z'-factor ≥0.5 were used for further analysis. All data points were measured in duplicates for each plate and were replicated in at least two plates.

Western blotting.

Cells were plated in six-well plates on day 1 at 400.000 (H1975) and 500.000 (A431) cells per well and incubated at 37 °C and 5% CO_2. On day 2 cells were starved over night with media containing 0.5% FBS to be incubated on day 3 for 1 h with EGFR inhibitors following an additional 30 min of EGF stimulation at 50 ng/mL. The media was discarded and cells were washed once with DPBS 1x. Using 100 µL per well of lysis buffer, cells were then scraped off and lysed while rotating (Stuart Scientific, Staffordshire, UK) for 1 h at 4 °C. Lysis buffer containing 1% (v/v) NP-40, 50 mM (v/v) Tris-HCl pH 8.0, 100 mM (m/v) sodium fluoride, 30 mM (m/v) sodium pyrophosphate, 2 mM (m/v) sodium molybdate, 5 mM (v/v) EDTA and 2 mM (m/v) sodium vanadate; adding 1% 10 mg/mL aprotinin and leupeptin as well as 10% 100 mM PMSF and 200 mM sodium vanadate shortly before use. Cells were centrifuged at 4 °C for 30 min at 14.000 rpm. Supernatant was transferred into new tube and protein concentrations were determined with the Bio-Rad Protein Assay (Bio-Rad Laboratories, Hercules, CA). Loading buffer containing 0.5 M (v/v) Tris-HCl pH 6.7, 10% (v/v) SDS, (w/v) DTT, (v/v) Glycerol, and 0.05% (w/v) bromophenol blue was added and lysates were incubated for 5 min at 95 °C. Proteins were separated by SDS-PAGE and blotted onto NC-membranes (GE Healthcare/Amersham-Biosciences, Freiburg, Germany) using X Cell II Blot module according to manufacturer's manual (Thermo Fisher Scientific, Waltham, MA). After blocking with 1x Net-G buffer (10x buffer containing 1.5 M (w/v) NaCl, 50 mM (w/v) EDTA, 500 mM (w/v) Tris 0.5% (v/v) Tween 20 and 0.4% (w/v) gelatine) membranes were incubated at 4 °C over night with the respective primary antibody (1:5.000 for β-actin, 1:1000 for all other antibodies). After washing, the membranes were incubated for 2 h at room temperature with the secondary antibody (anti-IgG-HRP 1:2000, CST, Frankfurt, Germany) and washed again. Changes in protein expression and phosphorylation as visualized by chemiluminescence using Amersham ECL Prime (GE Healthcare/Amersham-Biosciences, Freiburg, Germany); were

captured and quantified using a FUJI LAS3000 system with Science Lab 2001 ImageGauge 4.0 software (Fujifilm Medial Systems, Stamford CT, USA).

Antibodies.

Rabbit antibodies specific for phospho-EGFR (Tyr1045), EGFR, Akt, phospho-Akt (Ser473), p42/44 mitogen-activated protein kinase (MAPK), phospho-p44/42 MAPK (Thr202/Tyr204), S6 ribosomal protein, phospho-S6 ribosomal protein (Ser235/236), mouse anti-β-actin antibody as well as HRP-conjugated anti-rabbit and anti-mouse secondary antibodies were purchased from Cell Signaling (Frankfurt, Germany; Beverly, MA).

Mass spectrometry experiments.

We used the drug-resistant mutant EGFR-T790M variant for MS experiments. We incubated a mixture of 26.4 µM of protein with 79.2 µM of inhibitor in buffer (25 mM TRIS, 250 mM NaCl, 10% glycerol, 1 mM TCEP, pH 8) on ice for 1 h. We analyzed the aliquots by ESI-MS using an Thermo Fisher Scientific Dionex UltiMate 3000 HPLC system connected to a Thermo Fisher Scientific Velos Pro (2d ion trap). Therefore, 5 *µ*L of sample was injected and separated using a Vydac 214TP C4 5 *µ*m column (150 mm x 2.1 mm) starting at 20% of solvent B for 5 minutes followed by a gradient up to 90% of solvent B over 14 min with a flow rate of 210 *µ*L/min with 0.1% TFA in water as solvent A and 0.1% TFA in MeCN as solvent B. A mass range of 700 to 2000 m/z was scanned and raw data was deconvoluted and analyzed with MagTran software (Z.Q. Zhang, A.G. Marshall, *J. Am. Soc. Mass. Spectr.* **1998**, *9*, 225-233).

For nano-LC-MS/MS measurements the samples were incubated with 10 mM iodoacetamide for 30 min at RT in the dark, in order to carbamidomethylate all free cysteine residues. Next, 1 µg was digested using the broad specificity protease subtilisin (Sigma Aldrich) in a 1:10 ratio

(protease:protein) for 20 min at 56 °C. Digestion was quenched by addition of 1% TFA and 2 pmol were analyzed by nano-LC-MS/MS using a Orbitrap Fusion Lumos mass spectrometer online-coupled to an Ultimate 3000 nano RSLC system (both Thermo Scientific).

Peptides were preconcentrated on a 75 µm x 2 cm C18 trapping column for 5 min using 0.1% TFA (v/v) at a flow rate of 20 µL/min, followed by separation on a 75 µm x 50 cm C18 main column (both Pepmap, Thermo Scientific) with a 90 min LC gradient ranging from 5-70% B (84% acetonitrile in 0.1% formic acid) at a flow rate of 250 nL/min. MS survey scans were acquired from 300 to 1500 m/z at a resolution of 120,000 using the polysiloxane ion at 445.12002 m/z as lock mass. In top speed mode (3 s) the most intense ions with charge states >1 were isolated with a 1.2 Da window for fragmentation with both electron transfer dissociation (ETD) and Electron-Transfer/Higher-Energy Collision Dissociation (EThcD) using calibrated charge-dependent ETD parameters and a supplemental HCD energy of 30. MS/MS spectra were acquired in the Orbitrap with a resolution of 60,000. AGC target values were set to 200,000 and 100,000 for MS and MS/MS, respectively. Maximum injection times were 50 ms and 200 ms, respectively.

Raw files were searched with PEAKS 7.5 (*de novo* and DB node) (J. Zhang, L. Xin, B. Shan, W. Chen, M. Xie, D. Yuen, W. Zhang, Z. Zhang, G.A. Lajoie, Ma, B. *Molecular & cellular proteomics : MCP 11* **2012**, M111.010587) with automatic correction of precursor masses. The protein sequence of EGFR-T790M was fused to a *P. furiosus* database (2,045 target sequences, downloaded from UniProt in January 2016) (M. Vaudel, J.M. Burkhart, D.Breiter, R.P. Zahedi, A. Sickmann, L. Martens, *J. Proteome Res.* **2012**, *11*, 10, 5065-5071) and searches were conducted in a target/decoy mode to calculate a false discovery rate (FDR). Enzyme specificity was set to “none” and cysteine modifications by either carbamidomethylation (+57.02146 Da) or **19h** (+510.27432) were set as variable, with a maximum of 3 per peptide. Fragmentation mode was set to ETD to consider c and z ions for scoring. Mass tolerances were set to 10 ppm for precursor

masses and 0.02 Da for fragment ions. FDR was adjusted to 1% on peptide spectrum match (PSM) level.

Microsomal stability (phase I) assay.

Metabolic stability under oxidative conditions was measured in liver microsomes from different species by LC/MS-based measuring of depletion of compound at a concentration of 3 µM over time up to 50 minutes (HLM) or 60 minutes (MLM) at 37 °C. Based on the compound half-life $t_{1/2}$, the *in vitro* intrinsic clearance CL_{int} was calculated.

Plasma stability.

Plasma stability was measured by LC/MS-based determination of the percentage of remaining compounds at a concentration of 5 µM after incubation in plasma obtained from different species for 1 h at 37 °C.

Plasma protein binding.

Assessment of plasma protein binding was measured by equilibrium dialysis by incubating plasma with the compound of interest at a concentration of 5 µM for 6 h at 37 °C followed by LC/MS-based determination of final compound concentrations.

PAMPA (Parallel artificial membrane permeability assay).

The compound was diluted from a 10 mM stock in DMSO to a final concentration of 500 µM in 50 mM Hepes buffer pH 7.4 and transferred onto a transwell membrane covered with a membrane-forming solution of 10% 1,2-Dioleyl-*sn*-glycero-3-phosphocholine (Sigma Aldrich) and 0.5% (w/v) cholesterol (Sigma Aldrich) in dodecane. Following an incubation of 16 hours at room

temperature in a wet chamber, the optical density of the solution in the receiver well was measured between 250 and 500 nm in intervals of 10 nm. The percent flux was calculated from the AUC between 250 and 500 nm and normalized to the absorption of the compound following a 16 hour incubation in a parallel transwell containing a membrane covered with 50% MeOH in 50 mM Hepes buffer pH 7.4 (M. Kansy, F. Senner, K. Gubernator, *J Med Chem* **1998**, *41*, 1007-1010).

Caco-2 assay.

For Caco-2 cell assay, a 10 mM DMSO stock of the compound was diluted to a final concentration of 5 µM in HBSS buffer pH 7.4 and incubated for 2 hours at 37 °C and 5% CO_2 on a monolayer of Caco-2 cells (ATCC) that had been grown on a transwell membrane (Millipore, Schwalbach, Germany) for 21 days. The compound concentration was measured in the receiver as well as the donor well. Apparent permeability (P_{app}) from either the apical to basolateral direction or vice versa was calculated by the equation: $P_{app} = 1/AC_0\ (dQ/dt)$, where A is the membrane surface area, C_0 is the donor drug concentration at t = 0, and dQ/dt is the amount of drug transported within the given time period of 2 hours.

***In vivo* pharmacokinetics.**

To determine the pharmacokinetics of **19h**, totally 36 male RjOrl:SWISS (CD1) mice, age 8−10 weeks, body weight (bw) between 29 and 31 g, were purchased from Janvier, Saint-Berthevin Cedex, France. Mice were fed *ad libitum* with Allein-Futter für Ratten-/Mäusehaltung from Sniff Special Diets GmbH, Germany. They had free access to water and were kept in a 12 h day/night rhythm. All experiments were approved by the local authorities. Pharmacokinetics were studied for the oral (PO), intraperitoneal (IP), and intravenous (IV) route of administration. For the oral and

intraperitoneal routes, a stock solution of 2 mg/mL of **19h** in a solution consisting of 10% of DMSO and to 90% of an aqueous solution of 30% 2-hydroxypropyl-β-cyclodextrin was prepared. To reach doses of 20 mg/kg bw, 300 µL were administered per 30 g of mouse bw. For the intravenous route of administration, a 2 mg/mL DMSO solution was prepared and 30 µL were applied to a 30 g mouse to reach a concentration of 2 mg/kg bw. Mice were sacrificed 5, 15, 45, and 135 min after administration of the test compound, and approximately 100 µL of blood was taken from the left heart chamber. Three mice were analyzed per time point and route of administration (PO, IP and IV), respectively. Immediately after sampling, blood was centrifuged at 16200g for 10 min at 4 °C and plasma was stored at -80 °C until LC/MS analysis of **19h**. Prior to LC-MS/MS analysis, plasma proteins were precipitated with acetonitrile containing an internal standard, and samples were filtered. A calibration curve was obtained from spiked blank plasma samples. **19h** was measured using a Shimadzu UPLC system connected to a QTrap 4000 hybrid triple quadrupole/linear ion trap mass spectrometer (AB Sciex). The regression equation of the calibration curve was used to calculate plasma concentrations. Pharmacokinetic parameters were calculated using the PKSolver software2.

Synthetic procedures.

Unless otherwise noted, all commercially available compounds, reagents, solvents and anhydrous solvents were used as provided without further purification. ^{1}H and ^{13}C NMR spectra were recorded on a Bruker Avance DRX 300, DRX 400 or DRX 500 spectrometer or 400 MHz AVANCE-III HD and 600 MHz AVANCE-III HD (Bruker BioSpin GmbH) equipped with a 5 mm helium cooled broadband BBFO cryo probe from Bruker BioSpin GmbH 500 MHz DD2-500 (Agilent Technologies). Chemical shifts (δ) are reported in parts per million (ppm) and coupling constants (*J*) are expressed in Hertz (Hz). ^{1}H and ^{13}C spectra are referenced to the residual solvent signal DMSO-d_6 (2.50 or 39.52 ppm), $CDCl_3$ (7.26 or 77.16 ppm) or CD_3OD (1.94 or 49.00 ppm, respectively). High-resolution electrospray ionization mass spectra (ESI-FTMS) were recorded on a Thermo LTQ Orbitrap (high-resolution mass spectrometer from Thermo Electron) coupled to an “Accela” HPLC system supplied with a “Hypersil GOLD” column (Thermo Electron). Microwave reactions were performed using the Monowave 300 reactor by Anton Paar. Analytical TLC was carried out on Merck 60 F254 aluminum-backed silica gel plates and monitored by UV at λ = 254 and 366 nm. Compounds were purified by column chromatography using Baker silica gel (40-70 µm particle size) or Flash Chromatography on a Biotage Isolera One using Biotage SNAP, SNAP Ultra, ZIP Sphere or ZIP KP-Sil columns (25, 10, 5 or 120 g, respectively) or Grace Reverleris C18 40 µm columns (4 or 12 g) and monitored by UV at λ = 254 and 280 nm. Preparative HPLC was conducted on an Agilent HPLC system (1200 series) with a VP 125/21 Nucleodur C18 column from Macherey-Nagel and monitored by UV at λ = 210, 254 and 280 nm. All final compounds were purified to ≥95% purity confirmed by NMR analysis as well as LC/MS analysis on LCQ Advantage MAX (1200 series, Agilent) with Eclipse XDB-C18-column (5 µM 150 x 1.6 mm, Phenomenex).

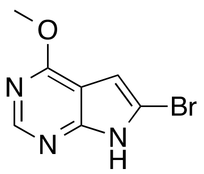

6-Bromo-4-methoxy-7*H*-pyrrolo[2,3-*d*]pyrimidine (3)

A solution of 6-Bromo-4-chloro-7*H*-pyrrolo[2,3-*d*]pyrimidine (**2**) (200.0 mg, 0.9 mmol) and sodium methoxide (4 mL, 25% w/w in MeOH) in MeOH (6 mL) was stirred at 70 °C until full conversion (3 d). The reaction mixture was poured into water, NaCl added and extracted with EtOAc. The combined organic layers were dried over Na_2SO_4 and the solvent removed *in vacuo*. Purification by column chromatography yielded the desired product as a white solid.

Yield: 87% (172.0 mg, 0.8 mmol). **^{1}H NMR** (500 MHz, DMSO-d_6) δ 12.83 (s, 1H), 8.36 (s, 1H), 6.60 (s, 1H), 4.02 ppm (s, 3H). **^{13}C NMR** (126 MHz, DMSO-d_6) δ 160.77, 152.83, 150.64, 108.51, 105.58, 99.99, 53.37 ppm. **HRMS:** *m/z* calc. for $[C_7H_6ON_3Br+H]^+$ 227.97670, found 227.97738, calc. for $[C_7H_6ON_3{}^{81}Br+H]^+$ 229.97465, found 229.97460.

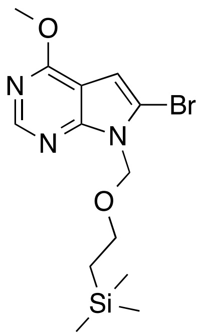

6-Bromo-4-methoxy-7-{[2-(trimethylsilyl)ethoxy]methyl}-7*H*-pyrrolo[2,3-*d*]pyrimidine (4)

To a solution of sodium hydride, 60% in paraffin oil (38.9 mg, 1.0 mmol, 1.30 eq.) in DMF (20 mL) at 0 °C was added compound **3** (170.6 mg, 0.8 mmol, 1.00 eq.) and stirred for 30 min. 2-(Trimethylsilyl)ethoxymethylchloride (135.1 µL, 0.8 mmol, 1.02 eq.) was added and stirred at rt overnight. The reaction mixture was poured into aqueous solution of NH_4Cl, NaCl added and extracted with EtOAc. The combined organic layers were dried over Na_2SO_4 and the solvent

removed *in vacuo*. Purification by column chromatography yielded the desired product as a clear oil.

Yield: 67% (181.6 mg, 0.5 mmol). **^{1}H NMR** (400 MHz, DMSO-d_6) δ 8.47 (s, 1H), 6.84 (s, 1H), 5.60 (s, 2H), 4.05 (s, 3H), 3.53 (t, J = 7.9 Hz, 2H), 0.82 (t, J = 7.9 Hz, 2H), -0.11 ppm (s, 9H). **^{13}C NMR** (101 MHz, DMSO-d_6) δ 161.11, 152.52, 151.20, 112.36, 105.05, 101.61, 71.33, 65.79, 53.75, 17.03, -1.42 ppm. **HRMS:** *m/z* calc. for $[C_{13}H_{20}O_2N_3BrSi+H]^+$ 358.05809, found 358.05953, calc. for $[C_{13}H_{20}O_2N_3{}^{81}BrSi+H]^+$ 360.05605, found 360.05596.

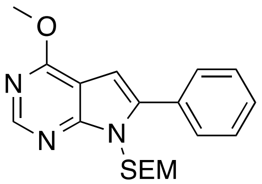

4-Methoxy-6-phenyl-7-{[2-(trimethylsilyl)ethoxy]methyl}-7*H*-pyrrolo[2,3-*d*]pyrimidine (5)

A solution of compound **4** (247.5 mg, 0.7 mmol, 1.00 eq.), phenylboronic acid (101.1 mg, 0.8 mmol, 1.20 eq.) and Na_2CO_3 (111.9 mg, 1.0 mmol, 1.50 eq.) in DMF:H_2O (10:2 mL) was degassed with Ar for 30 min at rt. Then $Pd(PPh_3)_4$ (119.7 mg, 103.6 µmol, 0.15 eq.) was added and the reaction stirred at 80 °C overnight. The reaction mixture was poured into aqueous solution of NH_4Cl, NaCl added and extracted with EtOAc. The combined organic layers were dried over Na_2SO_4 and the solvent removed *in vacuo*. Purification by column chromatography yielded the desired product as a clear oil.

Yield: 84% (207.8 mg, 0.6 mmol). **^{1}H NMR** (400 MHz, DMSO-d_6) δ 8.50 (s, 1H), 7.77 (s, 1H), 7.75 (s, 1H), 7.57–7.42 (m, 3H), 6.74 (s, 1H), 5.59 (s, 2H), 4.07 (s, 3H), 3.59 (t, J = 8.0 Hz, 2H), 0.83 (t, J = 8.0 Hz, 2H), -0,11 ppm (s, 9H). **^{13}C NMR** (101 MHz, DMSO-d_6) δ 162.10, 153.73, 150.94, 139.66, 130.94, 128.86, 128.82, 128.68, 104.59, 98.37, 70.69, 65.91, 53.61, 17.26, -1.46 ppm. **HRMS:** *m/z* calc. for $[C_{19}H_{25}O_2N_3Si+H]^+$ 356.17888, found 356.17975.

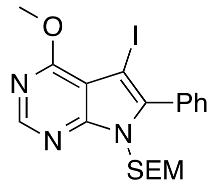

5-Iodo-4-methoxy-6-phenyl-7-{[2-(trimethylsilyl)ethoxy]methyl}-7*H*-pyrrolo[2,3-*d*]pyrimidine (6)

A solution of compound **5** (189.2 mg, 0.5 mmol, 1.0 eq.) and *N*-iodosuccinimide (179.6 mg, 0.8 mmol, 1.5 eq.) in MeCN (6 mL) was stirred at rt until full conversion (3 h). The reaction mixture was poured into water, NaCl added and extracted with EtOAc. The combined organic layers were dried over Na_2SO_4 and the solvent removed *in vacuo*. Purification by column chromatography yielded the desired product as a clear oil.

Yield: 97% (249.8 mg, 0.52 mmol). **^{1}H NMR** (400 MHz, DMSO-d_6) δ 8.51 (s, 1H), 7.54 (s, 5H), 5.44 (s, 2H), 4.08 (s, 3H), 3.40 (t, J = 8.3 Hz, 2H), 0.73 (t, J = 8.2 Hz, 2H), -0.14 ppm (s, 9H). **^{13}C NMR** (101 MHz, DMSO-d_6) δ 162.09, 152.77, 151.16, 140.47, 130.94, 130.24, 129.30, 128.44, 106.58, 71.23, 65.86, 54.94, 53.85, 17.15, -1.48 ppm. **HRMS:** *m/z* calc. for $[C_{19}H_{24}O_2N_3ISi+H]^+$ 482.07552, found 482.07424.

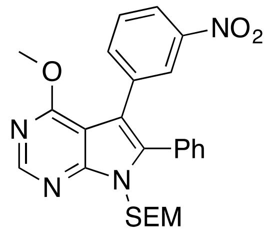

4-Methoxy-5-(3-nitrophenyl)-6-phenyl-7-{[2-(trimethylsilyl)ethoxy]methyl}-7*H*-pyrrolo[2,3-*d*]pyrimidine (7)

A solution of compound **6** (226.7 mg, 0.5 mmol, 1.00 eq.), *m*-Nitrobenzeneboronic acid (94.3 mg, 0.6 mmol, 1.20 eq.) and Na_2CO_3 (76.3 mg, 0.7 mmol, 1.50 eq.) in DMF:H_2O (2.5:0.5 mL) was

degassed with Ar for 30 min at rt. Then $Pd(PPh_3)_4$ (81.6 mg, 70.6 µmol) was added and the reaction stirred at 80 °C overnight. The reaction mixture was poured into aqueous solution of NH_4Cl, NaCl added and extracted with EtOAc. The combined organic layers were dried over Na_2SO_4 and the solvent removed *in vacuo*. Purification by column chromatography yielded the desired product as a yellow oil.

Yield: 89% (201.9 mg, 0.4 mmol). **^{1}H NMR** (500 MHz, DMSO-*d*$_6$) δ 8.57 (s, 1H), 8.09–8.07 (m, 1H), 8.07–8.03 (m, 1H), 7.63–7.57 (m, 1H), 7.51 (t, *J* = 7.9 Hz, 1H), 7.45–7.38 (m, 5H), 5.51 (s, 2H), 3.97 (s, 3H), 3.49 (t, *J* = 8.2 Hz, 2H), 0.78 (t, *J* = 8.1 Hz, 2H), -0.11 ppm (s, 9H). **^{13}C NMR** (126 MHz, DMSO-*d*$_6$) δ 162.45, 152.49, 151.30, 147.12, 136.99, 136.92, 134.98, 131.07, 129.06, 128.89, 128.55, 125.11, 121.11, 111.64, 103.00, 70.62, 65.88, 59.66, 53.66, 17.19, -1.55 ppm. **HRMS:** *m/z* calc. for $[C_{25}H_{28}O_4N_4Si+H]^+$ 477.19526, found 477.19402.

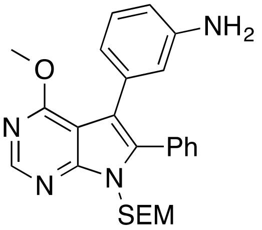

3-(4-Methoxy-6-phenyl-7-{[2-(trimethylsilyl)ethoxy]methyl}-7*H*-pyrrolo[2,3-*d*]pyrimidin-5-yl)anilin (8)

A solution of compound **7** (168.8 mg, 0.3 mmol, 1.0 eq.), ammonium formate (178.7 mg, 2.83 mmol, 8.0 eq.) and palladium, 5% on charcoal (75.4 mg, 35.4 mmol, 0.1 eq.) in EtOH (10 mL) was stirred at 90 °C until full conversion (1 h). The reaction mixture was filtered through Celite, poured into an aqueous solution of $NaHCO_3$, NaCl added and extracted with EtOAc. The combined organic layers were dried over Na_2SO_4 and the solvent removed *in vacuo*. Purification by column chromatography yielded the desired product as a yellow oil.

Yield: quant. (160.2 mg, 0.4 mmol). **^{1}H NMR** (500 MHz, DMSO-d_6) δ 8.50 (s, 1H), 7.37 (s, 5H), 6.84 (t, J = 7.8 Hz, 1H), 6.51 (t, J = 1.8 Hz, 1H), 6.43–6.39 (m, 1H), 6.33–6.29 (m, 1H), 5.47 (s, 2H), 4.87 (s, 2H), 3.93 (s, 3H), 3.48 (t, J = 8.1 Hz, 2H), 0.78 (t, J = 8.0 Hz, 2H), -0.11 ppm (s, 9H). **^{13}C NMR** (126 MHz, DMSO-d_6) δ 162.59, 152.30, 150.73, 147.69, 135.36, 133.80, 130.91, 130.13, 128.31, 128.08, 127.69, 118.70, 116.71, 114.80, 112.35, 103.51, 70.54, 65.73, 53.46, 17.21, -1.53 ppm. **HRMS:** *m/z* calc. for $[C_{25}H_{30}O_2N_4Si+H]^+$ 447.22108, found 447.22057.

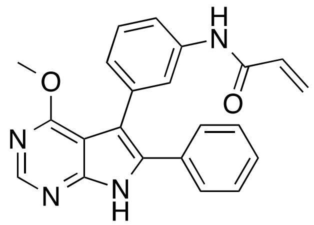

***N*-[3-(4-Methoxy-6-phenyl-7*H*-pyrrolo[2,3-*d*]pyrimidin-5-yl)phenyl]acrylamide (1a)**

To a solution of compound **8** (50.0 mg, 0.1 mmol, 1.0 eq.) and DIPEA (195.0 µL, 1.1 mmol, 10.0 eq.) in THF (1.5 mL) was added at 0 °C drop-wise a solution of acryloyl chloride (10.0 µL, 0.1 mmol, 1.1 eq.) in THF (1 mL) and stirred until full conversion (2.5 h). The reaction mixture was poured into aqueous solution of $NaHCO_3$, NaCl added and extracted with EtOAc. The combined organic layers were dried over Na_2SO_4 and the solvent removed *in vacuo*. Purification by column chromatography yielded the intermediate as clear oil. This was directly taken up in CH_2Cl_2 (1.5 mL) at 0 °C, after which TFA was added (0.5 mL) and the reaction stirred at rt until full conversion (1 h). The reaction mixture was added to aqueous solution of $NaHCO_3$, NaCl added and extracted with CH_2Cl_2. The combined organic layers were dried over Na_2SO_4 and the solvent removed *in vacuo*. Purification by preparative HPLC yielded the desired product as a white solid.

Yield: 54% (20.8 mg, 56.2 µmol) over two steps. **^{1}H NMR** (600 MHz, DMSO-d_6) δ 10.57 (s, 1H), 8.41 (s, 1H), 7.77 (d, J = 7.7 Hz, 1H), 7.69 (s, 1H), 7.39 (d, J = 7.3 Hz, 2H), 7.38–7.32 (m, 2H), 7.30–7.23 (m, 3H), 6.96 (d, J = 7.7 Hz, 1H), 6.59 (dd, J = 17.0, 10.1 Hz, 1H), 6.21 (dd, J = 17.0,

1.8 Hz, 1H), 5.70 (dd, *J* = 10.1, 1.8 Hz, 1H), 5.32 (t, *J* = 5.0 Hz, 1H), 3.88 ppm (s, 3H). **HRMS:** *m/z* calc. for $[C_{22}H_{18}O_2N_4+H]^+$ 371.15025, found 371.15003.

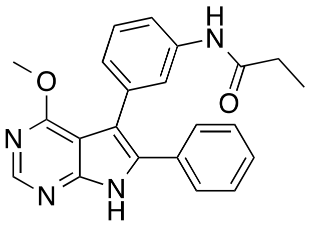

***N*-[3-(4-Methoxy-6-phenyl-7*H*-pyrrolo[2,3-*d*]pyrimidin-5-yl)phenyl]propionamide (1b)**

To a solution of compound **8** (50.0 mg, 0.1 mmol, 1.0 eq.) and DIPEA (195.0 µL, 1.1 mmol, 10.0 eq.) in THF (1.5 mL) was added at 0 °C drop-wise a solution of propionyl chloride (10.1 µL, 0.1 mmol, 1.1 eq.) in THF (1 mL) and the reaction was stirred at rt until full conversion (2.5 h). The reaction mixture was poured into aqueous solution of $NaHCO_3$, NaCl added and extracted with EtOAc. The combined organic layers were dried over Na_2SO_4 and the solvent removed *in vacuo*. Purification by column chromatography yielded the intermediate as clear oil. This was directly taken up in CH_2Cl_2 (1.5 mL) at 0 °C after which TFA (0.5 mL) was added and the reaction stirred at rt until full conversion (1 h). The reaction mixture was added to aqueous solution of $NaHCO_3$, NaCl added and extracted with CH_2Cl_2. The combined organic layers were dried over Na_2SO_4 and the solvent removed *in vacuo*. Purification by preparative HPLC yielded the desired product as a white solid.

Yield: 45% (20.2 mg, 54.2 µmol) over two steps. **^{1}H NMR** (500 MHz, DMSO-d_6) δ 10.10 (s, 1H), 8.40 (s, 1H), 7.66 (d, *J* = 8.6 Hz, 1H), 7.58 (s, 1H), 7.38 (d, *J* = 6.9 Hz, 2H), 7.32–7.19 (m, 5H), 6.90 (d, *J* = 7.9 Hz, 1H), 3.86 (s, 3H), 2.33–2.25 (m, 2H), 1.06–1.02 ppm (m, 3H). **HRMS:** *m/z* calc. for $[C_{22}H_{20}O_2N_4+H]^+$ 373.16590, found 373.16541.

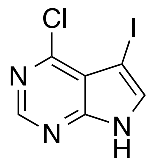

4-Chloro-5-iodo-7*H*-pyrrolo[2,3-*d*]pyrimidine

N-iodosuccinimide (12.3 g, 54.7 mmol, 1.05 eq.) in DMF (20 mL) was added over a period of 15 min to a solution of 4-chloro-7*H*-pyrrolo[2,3-*d*]pyrimidine (**11**) (8.00 g, 52.1 mmol, 1.00 eq.) in DMF (80 mL). The reaction was stirred at rt in the dark until full conversion (16 h). A saturated aqueous solution of $Na_2S_2O_3$ was subsequently added and the reaction mixture diluted with EtOAc. Then water was added and the mixture was extracted with EtOAc. The combined organic layers were washed with water and saturated solution of NaCl, dried over Na_2SO_4 and the solvent removed *in vacuo*. The desired product was isolated without further purification as a white solid.

Yield: 97% (14.1 g, 50.5 mmol). **^{1}H NMR** (500 MHz, DMSO-d_6) δ 12.95 (s, 1H), 8.59 (s, 1H), 7.93 ppm (d, *J* = 2.2 Hz, 1H). **^{13}C NMR** (500 MHz, DMSO-d_6) δ 151.5, 150.8, 150.5, 133.9, 115.8, 51.7 ppm. **LC/MS:** *m/z* calc. for $[C_6H_3N_3ICl+H]^+$ 280.47, found 280.18.

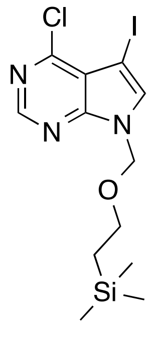

4-Chloro-5-iodo-7-{[2-(trimethylsilyl)ethoxy]methyl}-7*H*-pyrrolo[2,3-*d*]-pyrimidine (12)

Sodium hydride, 60% in paraffin oil (465.5 mg, 14.0 mmol, 1.3 eq.) was taken up in THF (25 mL) after which 4-chloro-5-iodo-7*H*-pyrrolo[2,3-*d*]pyrimidine (3.5 g, 9.0 mmol, 1.0 eq.) was added and the reaction stirred at 0 °C for 30 min. SEM-Cl (1.74 mL, 11.8 mmol, 1.1 eq.) was then added and the reaction mixture stirred at rt until full conversion. The reaction mixture was poured into water,

NaCl added and extracted with EtOAc. The combined organic layers were dried over Na_2SO_4 and the solvent removed *in vacuo*. Purification by column chromatography yielded the desired product as a white solid.

Yield: 79% (2.9 g, 7.1 mmol). **^{1}H NMR** (400 MHz, DMSO-d_6) δ 8.69 (s, 1H), 8.13 (s, 1H), 5.60 (s, 2H), 3.51 (t, J = 8.0 Hz, 2H), 0.81 (t, J = 8.0 Hz, 2H), -0.10 ppm (s, 9H). **^{13}C NMR** (101 MHz, DMSO-d_6) δ 151.19, 151.09, 150.91, 136.64, 116.26, 72.95, 66.09, 52.93, 17.07, -1.39 ppm. **HRMS:** *m/z* calc. for $[C_{12}H_{17}ON_3ClISi+H]^+$ 409.99469, found 409.99444, calc. for $[C_{12}H_{17}ON_3{}^{37}ClISi+H]^+$ 411.99174, found 411.99101.

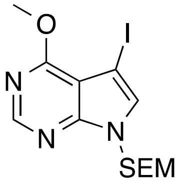

5-Iodo-4-methoxy-7-{[2-(trimethylsilyl)ethoxy]methyl}-7*H*-pyrrolo[2,3-*d*]-pyrimidine (13)

A solution of compound **12** (2.8 g, 6.9 mmol) and sodium methoxide (12 mL, 25% w/w in MeOH) in MeOH (18 mL) was stirred at 70 °C until full conversion (overnight). The reaction mixture was poured into water, NaCl added and extracted with EtOAc. The combined organic layers were dried over Na_2SO_4 and the solvent removed *in vacuo*. The desired product was isolated without further purification as a white solid.

Yield: 99% (2.8 g, 6.9 mmol). **^{1}H NMR** (300 MHz, DMSO-d_6) δ 8.46 (s, 1H), 7.76 (s, 1H), 5.54 (s, 2H), 4.05 (s, 3H), 3.49 (t, J = 8.1 Hz, 2H), 0.81 (t, J = 7.9 Hz, 2H), -0.10 ppm (d, J = 3.2 Hz, 9H). **^{13}C NMR** (75 MHz, DMSO-d_6) δ 162.33, 151.89, 151.29, 132.30, 106.34, 72.55, 65.75, 53.76, 50.90, 17.07, -1.40 ppm. **HRMS:** *m/z* calc. for $[C_{13}H_{20}O_2N_3ISi+H]^+$ 406.04422, found 406.04320.

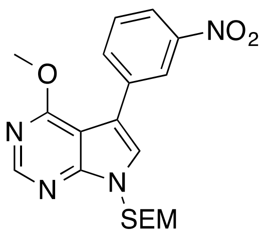

4-Methoxy-5-(3-nitrophenyl)-7-{[2-(trimethylsilyl)ethoxy]methyl}-7*H*-pyrrolo-[2,3-*d*]pyrimidine (14)

A solution of compound **13** (2.2 g, 5.5 mmol, 1.0 eq.), *m*-nitrobenzeneboronic acid (1.1 g, 6.6 mmol, 1.2 eq.), Na_2CO_3 (887.4 mg, 8.2 mmol, 1.5 eq.) and $Pd(PPh_3)_4$ (949.4 mg, 0.8 mmol, 0.15 eq.) in MeCN:H_2O (14:7 mL) was stirred at 70 °C for 3 h. The reaction mixture was poured into aqueous solution of $NaHCO_3$, NaCl added and extracted with EtOAc. The combined organic layers were dried over Na_2SO_4 and the solvent removed *in vacuo*. Purification by column chromatography yielded the desired product as a yellow oil.

Yield: 55% (1.2 g, 3.0 mmol). **^{1}H NMR** (400 MHz, DMSO-d_6) δ 8.61 (s, 1H), 8.54 (s, 1H), 8.15 (s, 1H), 8.13 (d, J = 2.3 Hz, 1H), 8.10 (s, 1H), 7.71 (t, J = 8.0 Hz, 1H), 5.64 (s, 2H), 4.06 (s, 3H), 3.57 (t, J = 8.0 Hz, 2H), 0.85 (t, J = 8.0 Hz, 2H), -0.09 ppm (s, 9H). **^{13}C NMR** (101 MHz, DMSO-d_6) δ 162.58, 152.82, 151.38, 147.92, 135.26, 134.40, 129.76, 126.93, 122.68, 121.04, 113.80, 102.19, 72.84, 65.88, 53.86, 17.11, -1.38 ppm. **HRMS:** *m/z* calc. for $[C_{19}H_{24}O_4N_4Si+H]^+$ 401.16396, found 401.16270.

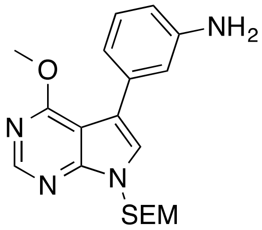

3-(4-methoxy-7-{[2-(trimethylsilyl)ethoxy]methyl}-7*H*-pyrrolo[2,3-*d*]pyrimidin-5-yl)aniline

A suspension of compound **14** (1.6 g, 4.0 mmol, 1.0 eq.), iron (1.1 g, 20.0 mmol, 5.0 eq.) and NH_4Cl (2.1 g, 40.0 mmol, 10 eq.) in a mixture of EtOH:H_2O (1:1) was sonicated at 60 °C for 3 h,

during which time the reaction mixture became a chocolate brown color. The reaction mixture was cooled to rt, filtered through Celite and washed with MeOH. After removal of the solvent *in vacuo*, the mixture was diluted with CH_2Cl_2 and water and the organic layer was separated. The aqueous layer was extracted with aliquots of CH_2Cl_2 and the combined organic layers were then washed with a saturated solution of NaCl, dried over $MgSO_4$ and filtered. After removal of the solvent *in vacuo*, the crude product was adsorbed onto silica and purified by flash chromatography, which afforded the pure compound as a light-brown semi-solid.

Yield: 87% (1.3 g, 3.5 mmol) **^{1}H NMR** (400 MHz, $CDCl_3$) δ 8.49 (s, 1H), 7.24 (s, 1H), 7.19 (dd [app. t], *J* = 7.8, 7.8 Hz, 1H), 7.06 (ddd, *J* = 8.0, 1.2, 0.8 Hz, 1H), 7.00 (s, 1H), 5.62 (s, 2H), 4.07 (s, 3H), 3.87 (br s, 2H), 3.56 (t, *J* = 8.0 Hz, 2H), 0.92 (t, *J* = 8.0 Hz, 2H), -0.06 ppm (s, 9H). **^{13}C NMR** (101 MHz, $CDCl_3$) δ 163.48, 153.01, 151.26, 146.01, 134.94, 129.12, 123.65, 119.53, 118.02, 115.80, 113.83, 103.37, 73.06, 66.49, 53.72, 17.76, -1.39 ppm. **LC/MS:** *m/z* calc. for $[C_{19}H_{26}N_4O_2Si+H]^+$ 371.19, found 371.24.

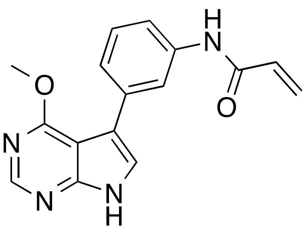

***N*-[3-(4-Methoxy-7*H*-pyrrolo[2,3-*d*]pyrimidin-5-yl)phenyl]acrylamide (9)**

To a solution of 3-(4-methoxy-7-{[2-(trimethylsilyl)ethoxy]methyl}-7*H*-pyrrolo[2,3-*d*]pyrimidin-5-yl)aniline (83.0 mg, 0.2 mmol, 1.0 eq.) and DIPEA (390 µL, 2.2 mmol, 10.0 eq.) in THF (2.5 mL) was added at 0 °C drop-wise a solution of acryloyl chloride (28.0 µL, 0.33 mmol, 1.5 eq.) in THF (1.0 mL). The reaction was stirred for 3 h, quenched with an aqueous solution of $NaHCO_3$ and extracted with CH_2Cl_2. The combined organic layers were dried over Na_2SO_4 and the solvent removed *in vacuo*. The crude intermediate was taken up in CH_2Cl_2 (15 mL), at rt added TFA

(5 mL) and stirred until full conversion. The solvent was removed *in vacuo* and the residual crude intermediate taken up in THF (10 mL). An aqueous solution of KOH (1 M, 10 mL) was added at rt and stirred for 1 h. The reaction mixture was neutralized with HCl (1 M) and extracted with CH_2Cl_2. The combined organic layers were dried over Na_2SO_4 and the solvent removed *in vacuo*. Purification by column chromatography yielded the desired product as white solid.

Yield: 60% (39 mg, 0.1 mmol). **^{1}H NMR** (500 MHz, DMSO-d_6) δ 12.30 (s, 1H), 10.18 (s, 1H), 8.42 (s, 1H), 8.03 (s, 1H), 7.59 (d, *J* = 7.9 Hz, 1H), 7.56 (d, *J* = 2.4 Hz, 1H), 7.31–7.39 (m, 2H), 6.49 (dd, *J* = 16.9, 10.2 Hz, 1H), 6.28 (dd, *J* = 16.9, 2.0 Hz, 1H), 5.74–5.79 (m, 1H), 4.02 ppm (s, 3H). **^{13}C NMR** (126 MHz, DMSO-d_6) δ 163.60, 163.08, 153.58, 151.09, 139.36, 135.37, 132.48, 128.90, 127.26, 124.22, 122.83, 120.08, 117.67, 116.14, 102.41, 53.84 ppm. **HRMS:** *m/z* calc. for $[C_{16}H_{14}N_4O_2+H]^+$ 295.11950, found 295.11866.

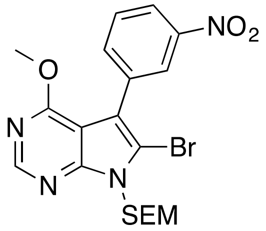

6-Bromo-4-methoxy-5-(3-nitrophenyl)-7-{[2-(trimethylsilyl)ethoxy]methyl}-7*H*-pyrrolo[2,3-*d*]pyrimidine

Compound **14** (1.1 g, 2.7 mmol, 1.0 eq.) was dissolved in MeCN (8 mL) after which *N*-bromosuccinimide (581.5 mg, 3.3 mmol, 1.2 eq.) added and the reaction stirred at rt for 1 h. The reaction mixture was poured into water, NaCl added and extracted with EtOAc. The combined organic layers were dried over Na_2SO_4 and the solvent removed *in vacuo*. Purification by column chromatography yielded the desired product as a yellow solid.

Yield: 85% (1.1 g, 2.3 mmol). **^{1}H NMR** (500 MHz, DMSO-d_6) δ 8.55 (d, *J* = 1.6 Hz, 1H), 8.36 (s, 1H), 8.26 (d, *J* = 8.0 Hz, 1H), 7.99 (d, *J* = 7.2 Hz, 1H), 7.78 (t, *J* = 8.0 Hz, 1H), 5.72 (s, 2H), 3.94

(d, J = 1.5 Hz, 3H), 3.61 (dd, J = 11.1, 4.5 Hz, 2H), 0.87 (t, J = 7.8 Hz, 2H), 0.08 ppm (s, 9H).**^{13}C NMR** (126 MHz, DMSO-d_6) δ 162.42, 153.30, 152.51, 148.27, 137.72, 134.62, 130.30, 125.69, 123.04, 114.38, 113.67, 104.24, 72.74, 66.88, 54.74, 17.96, -0.54 ppm. **HRMS:** *m/z* calc. for $[C_{19}H_{23}O_4N_4BrSi+H]^+$ 479.07447, found 479.07487, calc. for $[C_{19}H_{23}O_4N_4{}^{81}BrSi+H]^+$ 481.07242, found 481.07180.

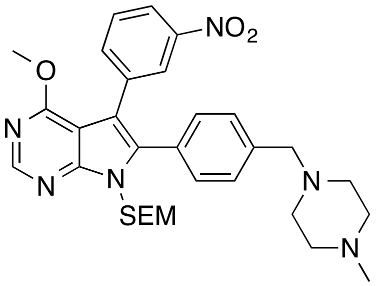

4-Methoxy-6-{4-[(4-methylpiperazin-1-yl)methyl]phenyl}-5-(3-nitrophenyl)-7-{[2-(trimethylsilyl)ethoxy]methyl}-*7H*-pyrrolo[2,3-*d*]pyrimidine (15)

A solution of 6-bromo-4-methoxy-5-(3-nitrophenyl)-7-{[2-(trimethylsilyl)ethoxy]methyl}-7*H*-pyrrolo[2,3-*d*]pyrimidine (342.0 mg, 0.7 mmol, 1.00 eq.), Na_2CO_3 (116.0 mg, 1.1 mmol, 1.50 eq.) and 4-[(4-methylpiperazin-1-yl)methyl]phenylboronic acid pinacol ester (271.8 mg, 0.9 mmol, 1.20 eq.) in dioxane:H_2O (5:1) was degased with Ar for 30 min. $Pd(PPh_3)_4$ (124.1 mg, 0.1 mmol, 0.15 eq.) was subsequently added and the reaction stirred for 2 d at 80 °C. The reaction mixture was poured into aqueous solution of $NaHCO_3$, NaCl added and extracted with 10% MeOH in CH_2Cl_2. The combined organic layers were dried over Na_2SO_4 and the solvent removed *in vacuo*. Purification by column chromatography yielded the desired product as a dark oil.

Yield: 50% (213.2 mg, 0.4 mmol). **^{1}H NMR** (500 MHz, DMSO-d_6) δ 8.57 (s, 1H), 8.05 (d, J = 8.2 Hz, 1H), 7.98 (s, 1H), 7.66 (d, J = 7.7 Hz, 1H), 7.53 (t, J = 8.0 Hz, 1H), 7.34 (s, 4H), 5.52 (s, 2H), 3.97 (s, 3H), 3.47 (d, J = 3.4 Hz, 2H), 2.34 (s, 8H), 2.16 (s, 3H), 0.78 (t, J = 8.1 Hz, 2H), -0.12 ppm (s, 9H). **^{13}C NMR** (126 MHz, DMSO-d_6) δ 162.42, 152.49, 151.27, 147.09, 139.47, 137.12, 136.96,

135.00, 130.91, 130.05, 128.97, 127.60, 124.97, 121.08, 114.94, 111.59, 102.98, 73.48, 70.66, 65.90, 61.56, 54.62, 53.69, 52.41, 48.55, 45.60, 24.90, 17.19, -1.52 ppm. **HRMS:** *m/z* calc. for $[C_{31}H_{40}O_4N_6Si+H]^+$ 589.29531, found 589.29592.

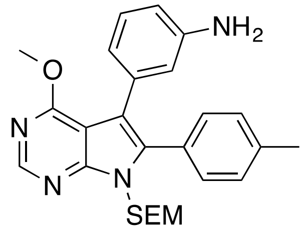

3-(4-Methoxy-6-(*p*-tolyl)-7-{[2-(trimethylsilyl)ethoxy]methyl}-7*H*-pyrrolo[2,3-*d*]pyrimidin-5-yl)aniline (16)

Compound **15** (543.0 mg, 0.9 mmol, 1.0 eq.) and palladium, 5% on charcoal (196.3 mg, 92.2 µmol, 0.1 eq.) were dissolved in EtOH (30 mL), heated to 90 °C, after which ammonium formate (465.3 mg, 7.4 mmol, 8.0 eq.) was added and the reaction stirred for 2 h. The reaction mixture was filtered through Celite, poured into water, NaCl added and extracted with 5% MeOH in CH_2Cl_2. The combined organic layers were dried over Na_2SO_4 and the solvent removed *in vacuo*. Purification by column chromatography yielded the desired product as a white solid.

Yield: 89% (380.0 mg, 0.8 mmol). **^{1}H NMR** (500 MHz, DMSO-d_6) δ 8.49 (s, 1H), 7.26 (d, *J* = 8.1 Hz, 2H), 7.18 (d, *J* = 8.1 Hz, 2H), 6.85 (t, *J* = 7.8 Hz, 1H), 6.51–6.48 (m, 1H), 6.42–6.38 (m, 1H), 6.32–6.29 (m, 1H), 5.46 (s, 2H), 4.89 (s, 2H), 3.92 (s, 3H), 3.52–3.43 (m, 2H), 2.32 (s, 3H), 0.91–0.65 (m, 2H), -0.11 ppm (s, 9H). **^{13}C NMR** (126 MHz, DMSO-d_6) δ 162.50, 152.26, 150.63, 147.68, 137.72, 135.45, 133.95, 130.79, 128.76, 127.74, 127.15, 118.72, 116.70, 114.56, 112.31, 103.54, 70.50, 65.74, 53.47, 20.81, 17.22, -1.51 ppm. **LC/MS:** *m/z* calc. for $[C_{26}H_{32}O_2N_4Si+H]^+$ 461.24, found 461.09.

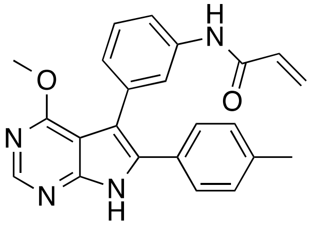

N-{3-[4-Methoxy-6-(p-tolyl)-7H-pyrrolo[2,3-d]pyrimidin-5-yl]phenyl}acrylamide (10a)

To a solution of compound **16** (70.0 mg, 0.2 mmol, 1.0 eq.) and DIPEA (260.1 μL, 1.5 mmol, 10.0 eq.) in THF (1 mL) was added at 0 °C drop-wise a solution of acryloyl chloride (13.6 μL, 0.2 mmol, 1.1 eq.) in THF (1 mL). The reaction was stirred until full conversion (1 h), quenched with an aqueous solution of $NaHCO_3$ and extracted with 5% MeOH in CH_2Cl_2. The combined organic layers were dried over Na_2SO_4 and the solvent removed *in vacuo*. The crude intermediate was taken up in CH_2Cl_2 (2 mL), after which TFA (1 mL) was added and the reaction stirred at rt for 2 h. The solvent was removed *in vacuo* and the residual crude intermediate taken up in THF (2 mL). An aqueous solution of NaOH (2 M, 1 mL) was added at rt and the reaction stirred until full conversion. The reaction mixture was diluted with CH_2Cl_2, the layers separated and the aqueous layer extracted with 5% MeOH in CH_2Cl_2. The combined organic layers were dried over Na_2SO_4 and the solvent removed *in vacuo*. Purification by column chromatography yielded the desired product as a yellow solid.

Yield: 29% (17.2 mg, 44.7 μmol) over two steps. **^{1}H NMR** (500 MHz, DMSO-d_6) δ 12.45 (s, 1H), 10.09 (s, 1H), 8.40 (s, 1H), 7.73 (d, J = 8.5 Hz, 1H), 7.56 (s, 1H), 7.26 (d, J = 8.0 Hz, 3H), 7.13 (d, J = 8.0 Hz, 2H), 6.98 (d, J = 7.7 Hz, 1H), 6.42 (dd, J = 17.0, 10.2 Hz, 1H), 6.23 (dd, J = 17.0, 2.0 Hz, 1H), 5.72 (dd, J = 10.2, 2.0 Hz, 1H), 3.87 (s, 3H), 2.28 ppm (s, 3H). **HRMS:** m/z calc. for $[C_{23}H_{20}O_2N_4+H]^+$ 385.16590, found 385.16649.

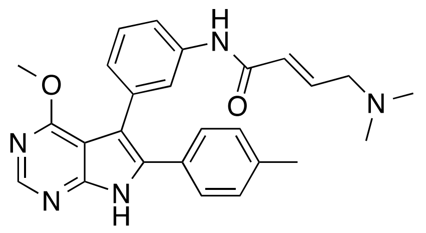

4-(Dimethylamino)-*N*-{3-[4-methoxy-6-(*p*-tolyl)-7*H*-pyrrolo[2,3-*d*]pyrimidin-5-yl]phenyl}but-2-enamide (10b)

4-(Dimethylamino)crotonic acid (50.3 mg, 0.3 mmol, 2 eq.) was dissolved in THF (1.5 mL) and oxalyl chloride (38.6 mg, 0.3 mmol, 2 eq.) was added at 0 °C. To this was added three drops of a solution of 5 drops DMF in 1 mL THF and the reaction further stirred at 0 °C. After 2 h the reaction mixture was added at 0 °C to a solution of compound **16** (70.0 mg, 0.2 mmol, 1 eq.) in THF (1.5 mL) and *N*-methylpyrrolidinone (0.75 mL). After completion of the reaction, aqueous solution of NaOH was added, the layers separated and the aqueous layer extracted with 10% MeOH in CH_2Cl_2. The combined organic layers were dried over Na_2SO_4 and the solvent removed *in vacuo*. The crude intermediate was taken up in CH_2Cl_2 (2 mL), and TFA (1 mL) was added at rt and the reaction stirred for 2 h. The solvent was removed *in vacuo* and the residual crude intermediate taken up in THF (2 mL). An aqueous solution of NaOH (2 M, 1 mL) was added at rt and stirred until full conversion. The reaction mixture was diluted with CH_2Cl_2, the layers separated and the aqueous layer extracted with 10% MeOH in CH_2Cl_2. The combined organic layers were dried over Na_2SO_4 and the solvent removed *in vacuo*. Purification by column chromatography yielded the desired product as a white solid.

Yield: 75% (50.8 mg, 0.1 mmol) over two steps. **^{1}H NMR** (500 MHz, DMSO-d_6) δ 12.45 (s, 1H), 10.03 (s, 1H), 8.40 (s, 1H), 7.72 (d, J = 8.1 Hz, 1H), 7.56 (s, 1H), 7.26 (t, J = 8.0 Hz, 3H), 7.12 (d, J = 8.0 Hz, 2H), 6.96 (d, J = 7.7 Hz, 1H), 6.70 (dt, J = 15.4, 5.9 Hz, 1H), 6.27 (d, J = 15.4 Hz, 1H), 3.87 (s, 3H), 3.06 (d, J = 5.6 Hz, 2H), 2.27 (s, 3H), 2.18 ppm (s, 6H). **^{13}C NMR** (126 MHz, DMSO-d_6) δ 163.08, 162.43, 152.19, 150.49, 138.81, 137.28, 135.00, 132.89, 128.98, 128.27, 128.17,

128.07, 126.71, 126.16, 125.92, 121.52, 117.56, 111.30, 104.63, 59.58, 53.22, 45.00, 20.71 ppm. **HRMS:** *m/z* calc. for $[C_{26}H_{27}O_2N_5+H]^+$ 442.22375, found 442.22377, calc. for $[C_{26}H_{27}O_2N_5+Na]^+$ 464.20570, found 464.20553.

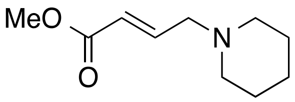

Methyl 4-(piperidin-1-yl)but-2-enoate

A solution of methyl 4-bromobut-2-enoate (2.0 g, 11.2 mmol, 1.0 eq.), piperidine (1.1 g, 12.3 mmol, 1.1 eq.) and DIPEA (1.63 mL, 12.3 mmol, 1.1 eq.) in THF (15 mL) was stirred at 50 °C for 16 h. The reaction mixture was then concentrated *in vacuo*, the residue taken in CH_2Cl_2 and washed with water. The organic layer was dried over Na_2SO_4 and the solvent removed *in vacuo*. Purification by column chromatography yielded the desired product as a yellow oil.

Yield: 75% (1.3 g, 7.2 mmol). **^{1}H NMR** (500 MHz, $CDCl_3$) δ 6.98 (dt, *J* = 15.7, 6.3 Hz, 1H), 5.97 (dt, *J* = 15.7, 1.6 Hz, 1H), 5.29 (s, 1H), 3.73 (s, 3H), 3.10 (dd, *J* = 6.3, 1.6 Hz, 2H), 2.39 (s, 4H), 1.63–1.54 (m, 4H), 1.47–1.39 ppm (m, 2H). **^{13}C NMR** (126 MHz, $CDCl_3$) δ 166.83, 146.08, 122.84, 77.16, 60.24, 54.84, 51.65, 26.08, 24.25 ppm. **LC/MS:** *m/z* calc. for $[C_{10}H_{17}O_2N+H]^+$ 184.13, found 185.98.

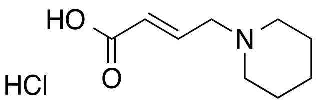

4-(Piperidin-1-yl)but-2-enoic acid hydrochloride

Methyl 4-(piperidin-1-yl)but-2-enoate (1.3 g, 6.8 mmol, 1.0 eq.) was dissolved in an aqueous solution of HCl (1 M, 15 mL) and heated to reflux until full conversion. To this was added toluene, the layers separated and the organic layer concentrated to dryness *in vacuo*. The desired product was isolated without further purification as a white solid.

Yield: 75% (1.1 g, 5.2 mmol). **^{1}H NMR** (500 MHz, DMSO-d_6) δ 12.73 (s, 1H), 11.28–10.72 (m, 1H), 6.95–6.82 (m, 1H), 6.17 (d, J = 15.6 Hz, 1H), 3.85 (s, 2H), 2.83 (s, 2H), 1.78 (s, 4H), 1.69 (d, J = 13.2 Hz, 1H), 1.37 ppm (d, J = 5.4 Hz, 1H). **^{13}C NMR** (126 MHz, DMSO-d_6) δ 165.86, 135.63, 129.26, 55.55, 51.78, 22.21, 21.16 ppm. **LC/MS:** *m/z* calc. for $[C_9H_{15}O_2N+H]^+$ 170.12, found 170.03.

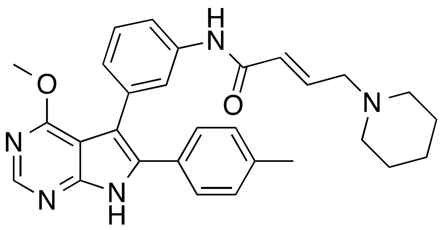

***N*-{3-[4-Methoxy-6-(*p*-tolyl)-7*H*-pyrrolo[2,3-*d*]pyrimidin-5-yl]phenyl}-4-(piperidin-1-yl)but-2-enamide (10c)**

4-(Piperidin-1-yl)but-2-enoic acid hydrochloride (44.6 mg, 0.2 mmol, 2 eq.) was dissolved in THF (1.5 mL) and oxalyl chloride (27.6 mg, 0.2 mmol, 2 eq.) was added at 0 °C. To this was added three drops of a solution of 5 drops DMF in 1 mL THF and the reaction was further stirred at 0 °C. After 2 h, this mixture was added at 0 °C to a solution of compound **16** (50.0 mg, 0.1 mmol, 1 eq.) in THF (1.0 mL) and *N*-methylpyrrolidinone (0.5 mL). After completion of the reaction, aqueous solution of NaOH was added, the layers separated and the aqueous layer extracted with 10% MeOH in CH_2Cl_2. The combined organic layers were dried over Na_2SO_4 and the solvent removed *in vacuo*. The crude intermediate was taken up in CH_2Cl_2 (2 mL), at rt added TFA (1 mL) and stirred for 2 h. The solvent was removed *in vacuo* and the residual crude intermediate taken up in THF (2 mL). An aqueous solution of NaOH (2 M, 1 mL) was added at rt and stirred until full conversion. The reaction mixture was diluted with CH_2Cl_2, the layers separated and the aqueous layer extracted with 10% MeOH in CH_2Cl_2. The combined organic layers were dried over Na_2SO_4

and the solvent removed *in vacuo*. Purification by column chromatography yielded the desired product as a white solid.

Yield: 19% (10.2 mg, 21.2 µmol) over three steps. **^{1}H NMR** (300 MHz, DMSO-d_6) δ 12.47 (s, 1H), 10.04 (s, 1H), 8.40 (s, 1H), 7.71 (d, *J* = 8.0 Hz, 1H), 7.55 (s, 1H), 7.27 (dd, *J* = 7.9, 5.2 Hz, 3H), 7.12 (d, *J* = 8.1 Hz, 2H), 6.96 (d, *J* = 7.9 Hz, 1H), 6.71 (d, *J* = 15.4 Hz, 1H), 6.26 (d, *J* = 15.9 Hz, 1H), 3.87 (s, 3H), 2.27 (s, 3H), 1.63–1.32 ppm (m, 9H). **HRMS:** *m/z* calc. for $[C_{29}H_{31}O_2N_5+H]^+$ 482.25505, found 482.25507.

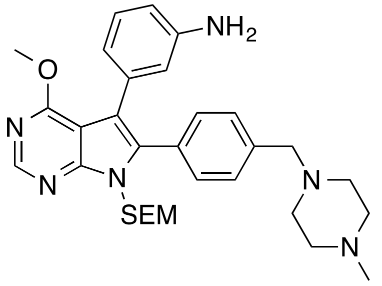

3-(4-Methoxy-6-{4-[(4-methylpiperazin-1-yl)methyl]phenyl}-7-{[2-(tri-methylsilyl)ethoxy]methyl}-*7H*-pyrrolo[2,3-*d*]pyrimidin-5-yl)aniline (18)

A solution of compound **15** (173.2 mg, 0.3 mmol, 1.0 eq.), ammonium formate (148.4 mg, 2.4 mmol, 8.0 eq.) and palladium, 5% on charcoal (62.6 mg, 29.4 µmol, 0.1 eq.) in EtOH (10 mL) was stirred at 90 °C until full conversion (1.5 h). The reaction mixture was filtered through Celite, poured into aqueous solution of $NaHCO_3$, NaCl added and extracted with 10% MeOH in CH_2Cl_2. The combined organic layers were dried over Na_2SO_4 and the solvent removed *in vacuo*. Purification by column chromatography yielded the desired product as a white solid.

Yield: 74% (122.3 mg, 0.2 mmol). **^{1}H NMR** (400 MHz, DMSO-d_6) δ 8.49 (s, 1H), 7.30 (q, *J* = 8.0 Hz, 4H), 6.83 (t, *J* = 7.7 Hz, 1H), 6.48 (s, 1H), 6.39 (d, *J* = 7.8 Hz, 1H), 6.29 (d, *J* = 7.3 Hz, 1H), 5.47 (s, 2H), 4.91 (s, 2H), 3.92 (s, 3H), 3.46 (d, *J* = 3.9 Hz, 2H), 3.17 (s, 2H), 2.37 (s, 8H), 2.17 (s, 3H), 0.76 (t, *J* = 8.0 Hz, 2H), -0.13 ppm (s, 9H). **^{13}C NMR** (101 MHz, DMSO-d_6) δ 162.59, 152.31,

150.79, 147.80, 135.38, 133.88, 130.81, 128.62, 127.79, 118.73, 116.73, 114.75, 112.36, 103.55, 70.62, 68.61, 65.82, 61.65, 54.62, 53.59, 52.44, 48.63, 45.58, 17.23, -1.43 ppm. **LC/MS**: *m/z* calc. for $[C_{31}H_{42}N_6O_2Si+H]^+$ 559.32, found 559.11.

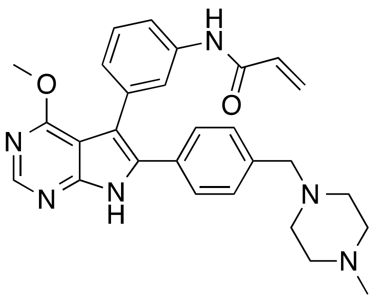

***N*-{3-[4-Methoxy-6-(4-{[4-methylpiperazin-1-yl)methyl]phenyl}-7*H*-pyrrolo[2,3-*d*]pyrimidin-5-yl]phenyl}acrylamide (17a)**

To a solution of compound **18** (50.0 mg, 84.9 µmol, 1.0 eq.) and DIPEA (147.8 µL, 0.8 mmol, 10.0 eq.) in THF (1 mL) was added at 0 °C drop-wise a solution of acryloyl chloride (7.5 µL, 93.4 µmol, 1.1 eq.) in THF (1 mL). The reaction was stirred until full conversion (3 h), quenched with an aqueous solution of $NaHCO_3$ and extracted with 10% MeOH in CH_2Cl_2. The combined organic layers were dried over Na_2SO_4 and the solvent removed *in vacuo*. The crude intermediate was taken up in CH_2Cl_2 (1.5 mL), at 0 °C and TFA (0.5 mL) was added after which the reaction was stirred until full conversion (1.5 h). The reaction was quenched with an aqueous solution of $NaHCO_3$ and extracted with 10% MeOH in CH_2Cl_2. The combined organic layers were dried over Na_2SO_4 and the solvent removed *in vacuo*. Purification by column chromatography yielded the desired product as a white solid.

Yield: quant. (44.4 mg, 92.0 µmol) over two steps. **^{1}H NMR** (500 MHz, DMSO-d_6) δ 12.49 (s, 1H), 10.09 (s, 1H), 8.42 (s, 1H), 7.70 (d, *J* = 8.2 Hz, 1H), 7.58 (s, 1H), 7.35 (d, *J* = 8.2 Hz, 2H), 7.32–7.22 (m, 3H), 7.00 (d, *J* = 7.7 Hz, 1H), 6.41 (dd, *J* = 17.0, 10.2 Hz, 1H), 6.22 (dd, *J* = 17.0, 1.9 Hz,

1H), 5.72 (dd, *J* = 10.2, 1.9 Hz, 1H), 3.88 (s, 3H), 3.49 (br s, 2H), 2.72 (br s, 4H), 2.45 (br s, 4H) ppm. **HRMS:** *m/z* calc. for $[C_{28}H_{30}O_2N_6Si+H]^+$ 483.25030, found 483.25029.

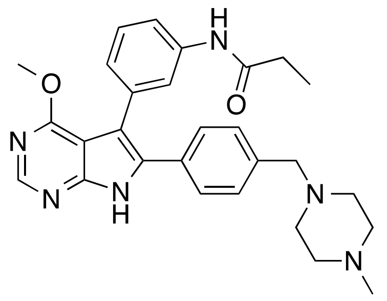

***N*-[3-(4-Methoxy-6-{4-[(4-methylpiperazin-1-yl)methyl]phenyl}-7*H*-pyrrolo[2,3-*d*]pyrimidin-5-yl)phenyl]propionamide (17b)**

To a solution of compound **18** (50.0 mg, 84.9 µmol, 1.0 eq.) and DIPEA (147.8 µL, 0.8 mmol, 10.0 eq.) in THF (1 mL) was added at 0 °C drop-wise a solution of propionyl chloride (7.7 µL, 93.4 µmol, 1.1 eq.) in THF (1 mL). The reaction was stirred until full conversion (2.5 h), quenched with an aqueous solution of $NaHCO_3$ and extracted with 10% MeOH in CH_2Cl_2. The combined organic layers were dried over Na_2SO_4 and the solvent removed *in vacuo*. The crude intermediate was taken up in CH_2Cl_2 (1.5 mL), at 0 °C after which TFA (0.5 mL) was added and the reaction stirred at rt until full conversion (1.5 h). The reaction was quenched with an aqueous solution of $NaHCO_3$ and extracted with 10% MeOH in CH_2Cl_2. The combined organic layers were dried over Na_2SO_4 and the solvent removed *in vacuo*. Purification by column chromatography yielded the desired product as a white solid.

Yield: 99% (47.3 mg, 84.6 µmol) over two steps. **^{1}H NMR** (500 MHz, DMSO-*d*$_6$) δ 12.45 (s, 1H), 9.85 (s, 1H), 8.40 (s, 1H), 7.63 (d, *J* = 8.1 Hz, 1H), 7.51 (s, 1H), 7.33 (d, *J* = 8.2 Hz, 2H), 7.25–7.19 (m, 3H), 6.93 (d, *J* = 7.6 Hz, 1H), 3.87 (s, 3H), 3.43 (s, 2H), 2.39 (br s, 8H), 2.28 (q, *J* = 7.6 Hz, 2H), 1.05 ppm (td, *J* = 7.3, 3.0 Hz, 3H). **^{13}C NMR** (126 MHz, DMSO-*d*$_6$) δ 171.89, 162.51, 158.00, 157.75, 152.20, 150.57, 139.00, 134.83, 129.85, 128.74, 128.07, 127.96, 125.45, 121.37,

118.41, 116.02, 111.77, 104.68, 61.39, 54.35, 53.21, 48.55, 29.47, 9.63 ppm. **HRMS:** *m/z* calc. for $[C_{28}H_{32}O_2N_6Si+H]^+$ 485.26595, found 485.26584.

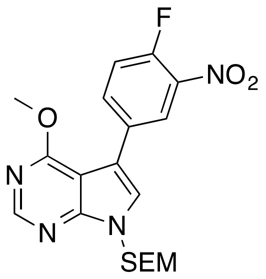

5-(4-Fluoro-3-nitrophenyl)-4-methoxy-7-{[2-(trimethylsilyl)ethoxy]methyl}-7*H*-pyrrolo[2,3-*d*]pyrimidine (21)

A Schlenk vessel was evacuated and filled with Ar three times. To this was added a mixture of dioxane:H_2O (3:1), *via* syringe, under positive Ar pressure. The solvent was degassed using 3-5 cycles of freeze-pump-thaw technique and subsequently degassed for 15 min by Ar purge. Another Schlenk vessel was charged with 5-iodo-4-methoxy-7-{[2-(trimethylsilyl)ethoxy]methyl}-7*H*-pyrrolo[2,3-*d*]pyrimidine (**13**) (1.0 g, 2.5 mmol, 1.0 eq.), (4-fluoro-3-nitrophenyl)boronic acid (547.5 mg, 3.0 mmol, 1.2 eq.), $Pd(PPh_3)_4$ (570.2 mg, 0.5 mmol, 0.2 eq.) and Na_2CO_3 (653.8 mg, 6.2 mmol, 2.5 eq.) and evacuated and filled with Ar three times. The degassed solvent (15 mL) was added to this Schlenk *via* syringe under positive Ar pressure. The sealed reaction mixture was then heated to 80 °C and stirred overnight (16 h), during which time the reaction mixture changed to a reddish brown color. The reaction mixture was cooled to rt, filtered through Celite, diluted with EtOAc and water and the organic layer was separated. The aqueous layer was extracted with EtOAc and the combined organic layers were then washed with a saturated solution of NaCl, dried over $MgSO_4$ and filtered. After removal of the solvent *in vacuo*, the crude product was adsorbed onto silica and purified by flash chromatography. Pure fractions were evaporated to dryness to afford the desired compound as a yellow semi-solid.

Yield: 86% (890.1 mg, 2.2 mmol). **^{1}H NMR** (700 MHz, DMSO-d_6) δ 8.50 (s, 1H), 8.49 (dd, J = 7.3, 2.2 Hz, 1H), 8.09 (ddd, J = 8.8, 3.9, 2.4 Hz, 1H), 8.03 (s, 1H), 7.62 (dd, J = 11.1, 8.8 Hz, 1H), 5.61 (s, 2H), 4.05 (s, 3H), 3.56 (t, J = 8.0 Hz, 2H), 0.84 (t, J = 8.0, 2H), -0.09 ppm (s, 9H). **^{13}C NMR** (700 MHz, DMSO-d_6) δ 162.5, 152.7, 151.3, 136.6, 135.5, 130.9, 126.7, 125.2, 118.4, 118.3, 112.9, 102.1, 72.8, 65.9, 53.8, 17.1, -1.45 ppm. **LC/MS:** *m/z* calc. for $[C_{19}H_{23}FN_4O_4Si+H]^+$ 419.15, found 419.15.

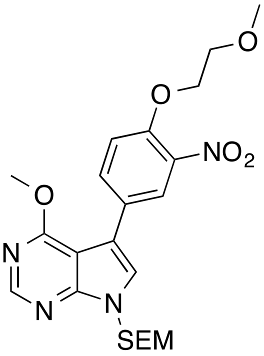

4-Methoxy-5-[4-(2-methoxyethoxy)-3-nitrophenyl]-7-{[2-(trimethylsilyl)ethoxy]methyl}-7*H*-pyrrolo[2,3-*d*]pyrimidine (22a)

To a suspension of sodium hydride, 60% dispersion in paraffin oil (100.9 mg, 2.5 mmol, 2.4 eq.) in THF was added a solution of 2-methoxyethan-1-ol (800.1 mg, 10.5 mmol, 10.0 eq.) in THF drop-wise over 5 min. After allowing the reaction mixture to stir for 30 min at rt, it was subsequently cooled to 0 °C and a solution of compound **21** (440.0 mg, 1.1 mmol, 1.0 eq.) in THF was added drop-wise over 10 min (100 mL). The reaction mixture was allowed to warm to rt and stirred overnight (16 h). The reaction mixture was then diluted with water and EtOAc and the organic layer was separated. The aqueous layer was extracted with aliquots of EtOAc and the combined organic layers were then washed with a saturated solution of $NaHCO_3$, a saturated solution of NaCl, dried over $MgSO_4$ and filtered. After removal of the solvent *in vacuo*, the crude product was

adsorbed onto silica and purified by flash chromatography. Pure fractions were evaporated to dryness to afford the desired compound as a yellow semi-solid.

Yield: 71% (354.0 mg, 0.7 mmol). **^{1}H NMR** (500 MHz, $CDCl_3$) δ 8.51 (s, 1H), 8.23 (d, *J* = 2.2 Hz, 1H), 7.78 (dd, *J* = 8.7, 2.2 Hz, 1H), 7.30 (s, 1H), 7.14 (d, *J* = 8.7 Hz, 1H), 5.65 (s, 2H), 4.29 (t, *J* = 5.0, 2H), 4.10 (s, 3H), 3.82 (t, *J* = 5.0, 2H), 3.56 (t, *J* = 8.1, 2H), 3.46 (s, 3H), 0.92 (t, *J* = 8.1, 2H), -0.06 ppm (s, 9H). **^{13}C NMR** (126 MHz, $CDCl_3$) δ 163.41, 152.95, 151.59, 151.18, 139.89, 133.99, 127.05, 125.97, 123.69, 115.49, 115.05, 103.21, 73.24, 70.79, 69.82, 66.76, 59.62, 54.05, 17.80, -1.34 ppm. **LC/MS:** *m/z* calc. for $[C_{22}H_{30}N_4O_6Si+H]^+$ 475.20, found 474.95.

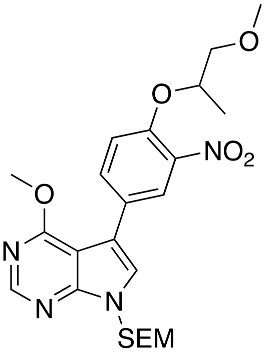

4-Methoxy-5-{4-[(1-methoxypropan-2-yl)oxy]-3-nitrophenyl}-7-{[2-(trimethylsilyl)ethoxy]methyl}-7*H*-pyrrolo[2,3-*d*]pyrimidine (22b)

To a suspension of sodium hydride, 60% dispersion in paraffin oil (114.7 mg, 2.9 mmol, 2.4 eq.) in THF was added a solution of 1-methoxypropan-2-ol (1.1 g, 11.9 mmol, 10.0 eq.) in THF drop-wise over 5 min. After allowing the reaction mixture to stir for 30 min at rt it was subsequently cooled 0 °C and a solution of compound **21** (500.0 mg, 1.2 mmol, 1.0 eq.) in THF was added drop-wise over 10 min (110 mL). The reaction mixture was allowed to warm to rt and stirred overnight (16 h). The reaction mixture was then diluted with water and EtOAc and the organic layer was separated. The aqueous layer was extracted with aliquots of EtOAc and the combined organic layers were then washed with a saturated solution of $NaHCO_3$, a saturated solution of NaCl, dried over $MgSO_4$

and filtered. After removal of the solvent *in vacuo*, the crude product was adsorbed onto silica and purified by flash chromatography. Pure fractions were evaporated to dryness to afford the desired compound as a yellow semi-solid.

Yield: 73% (426.0 mg, 0.9 mmol). **^{1}H NMR** (700 MHz, $CDCl_3$) δ 8.52 (s, 1H), 8.18 (d, *J* = 2.1 Hz, 1H), 7.76 (dd, *J* = 8.7, 2.1 Hz, 1H), 7.31 (s, 1H), 7.20 (d, *J* = 8.7 Hz, 1H), 5.66 (s, 2H), 4.71 – 4.68 (m, 1H), 4.12 (s, 3H), 3.68–3.64 (m, 1H), 3.57 (t, *J* = 8.4 Hz, 2H), 3.56–3.54 (m, 1H), 3.56–3.54 (m, 1H), 3.42 (s, 3H), 1.40 (d, *J* = 6.3 Hz, 3H), 0.93 (t, *J* = 8.4 Hz, 2H), -0.05 ppm (s, 9H). **^{13}C NMR** (176 MHz, $CDCl_3$) δ 163.48, 152.93, 151.54, 150.48, 141.04, 133.65, 127.00, 125.77, 123.73, 116.80, 115.70, 103.29, 76.52, 76.07, 73.34, 66.82, 59.60, 54.13, 17.87, 17.12, -1.31 ppm. **LC/MS:** *m/z* calc. for $[C_{23}H_{32}N_4O_6Si+H]^+$ 489.22, found 488.99.

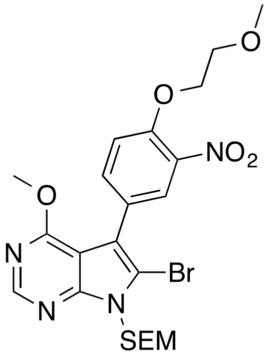

6-Bromo-4-methoxy-5-[4-(2-methoxyethoxy)-3-nitrophenyl]-7-{[2-(trimethylsilyl)ethoxy]methyl}-7*H*-pyrrolo[2,3-*d*]pyrimidine

Over a period of 5 min, a solution of *N*-bromosuccinimide (51.2 mg, 0.3 mmol, 1.05 eq.) in CH_2Cl_2 was added drop-wise to a solution of **22a** (130.0 mg, 0.3 mmol, 1.00 eq.) in CH_2Cl_2 (3 mL). The reaction flask was covered in foil and allowed to stir in darkness at rt overnight (16 h). The reaction mixture developed an orange-brown color, which reverted to colorless upon addition of a sufficient amount of aqueous $Na_2S_2O_3$ (10 mol%). The reaction mixture was then diluted with CH_2Cl_2 and water and the organic layer was separated. The aqueous layer was extracted with

aliquots of CH_2Cl_2 and the combined organic layers were then washed with a saturated solution of NaCl, dried over $MgSO_4$ and filtered. After removal of the solvent *in vacuo*, the crude product was adsorbed onto silica and purified by flash chromatography. Pure fractions were evaporated to dryness to afford the desired compound as a yellow solid.

Yield: 75% (114.0 mg, 0.2 mmol). **^{1}H NMR** (400 MHz, $CDCl_3$) δ 8.50 (s, 1H), 8.06 (d, *J* = 2.2 Hz, 1H), 7.71 (dd, *J* = 8.7, 2.3 Hz, 1H), 7.17 (d, *J* = 8.7 Hz, 1H), 5.76 (s, 2H), 4.32 (t, *J* = 4.5 Hz, 2H), 4.03 (s, 3H), 3.84 (t, *J* = 4.8 Hz, 2H), 3.64 (t, *J* = 8.0 Hz, 2H), 3.49 (s, 3H), 0.95 (t, *J* = 8.4 Hz, 2H), -0.04 (s, 9H). **^{13}C NMR** (101 MHz, $CDCl_3$) δ 162.24, 152.71, 151.62, 151.61, 139.64, 136.03, 128.06, 125.35, 114.39, 114.35, 111.88, 104.38, 72.21, 70.80, 69.80, 66.98, 59.69, 54.25, 17.89, -1.30. **LC/MS:** *m/z* calc. for $[C_{22}H_{29}BrN_4O_6Si+H]^+$ 553.11, found 552.89.

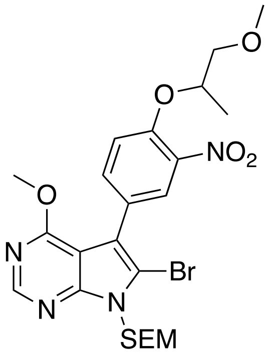

6-Bromo-4-methoxy-5-{4-[(1-methoxypropan-2-yl)oxy]-3-nitrophenyl}-7-{[2-(trimethylsilyl)ethoxy]methyl}-7*H*-pyrrolo[2,3-*d*]pyrimidine

Over a period of 5 min, a solution of *N*-bromosuccinimide (110.2 mg, 0.6 mmol, 1.05 eq.) in CH_2Cl_2 was added drop-wise to a solution of **22b** (288.0 mg, 0.6 mmol, 1.00 eq.) in CH_2Cl_2 (6 mL). The reaction flask was covered in foil and allowed to stir in darkness at rt overnight (16 h). The reaction mixture developed an orange-brown color, which reverted to colorless upon addition of a sufficient amount of aqueous $Na_2S_2O_3$ (10 mol%). The reaction mixture was then diluted with CH_2Cl_2 and water and the organic layer was separated. The aqueous layer was extracted with

aliquots of CH_2Cl_2 and the combined organic layers were then washed with a saturated solution of NaCl, dried over $MgSO_4$ and filtered. After removal of the solvent *in vacuo*, the crude product was adsorbed onto silica and purified by flash chromatography. Pure fractions were evaporated to dryness to afford the desired compound as a yellow solid.

Yield: 71% (237.2 mg, 0.4 mmol). **^{1}H NMR** (500 MHz, $CDCl_3$) δ 8.53 (s, 1H), 8.06 (d, *J* = 2.2 Hz, 1H), 7.74 (dd, *J* = 8.8, 2.2 Hz, 1H), 7.27 (d, *J* = 8.8 Hz, 1H), 5.79 (s, 2H), 4.79 – 4.74 (m, 1H), 4.07 (s, 3H), 3.75–3.70 (m, 1H), 3.68 (t, *J* = 8.3 Hz, 2H), 3.63–3.59 (m, 1H), 3.48 (s, 2H), 1.46 (d, *J* = 6.3 Hz, 3H), 1.00 (t, *J* = 8.3 Hz, 2H), 0.00 (s, 9H). **^{13}C NMR** (126 MHz, $CDCl_3$) δ 162.24, 152.79, 151.72, 150.85, 140.48, 135.73, 127.85, 125.17, 115.80, 114.32, 111.75, 104.32, 76.31, 76.02, 72.12, 66.94, 59.62, 54.13, 17.85, 17.08, -1.31. **LC/MS:** *m/z* calc. for $[C_{23}H_{31}BrN_4O_6Si+H]^+$ 567.13, found 567.26.

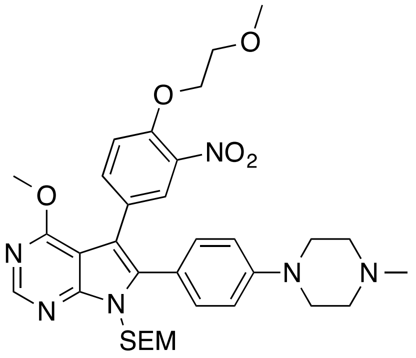

4-Methoxy-5-[4-(2-methoxyethoxy)-3-nitrophenyl]-6-[4-(4-methylpiperazin-1-yl)phenyl]-7-{[2-(trimethylsilyl)ethoxy]methyl}-7*H*-pyrrolo[2,3-*d*]pyrimidine

A Schlenk vessel was evacuated and filled with Ar three times. To this was added a mixture of dioxane:H_2O (3:1), *via* syringe, under positive pressure Ar. The solvent was degassed using 3-5 cycles of freeze-pump-thaw technique and subsequently degassed for 15 min by Ar purge. Another Schlenk vessel was charged with 6-bromo-4-methoxy-5-[4-(2-methoxyethoxy)-3-nitrophenyl]-7-{[2-(trimethylsilyl)ethoxy]methyl}-7*H*-pyrrolo[2,3-*d*]pyrimidine (314.0 mg, 0.6 mmol,

1.0 eq.), 4-(4-methylpiperazin-1-yl)phenylboronic acid pinacol ester (205.0 mg, 0.7 mmol, 1.2 eq.), $Pd(PPh_3)_4$ (138.7 mg, 0.1 mmol, 0.2 eq.) and Na_2CO_3 (159.0 mg, 1.5 mmol, 2.5 eq.) and evacuated and filled with Ar three times. The degassed solvent was added to this Schlenk *via* syringe under positive pressure Ar (3 mL). The sealed reaction mixture was then heated to 80 °C and stirred overnight (16 h), during which time the reaction mixture changed to a reddish brown colour. The reaction mixture was cooled to rt, filtered through Celite, diluted with EtOAc and water and the organic layer was separated. The aqueous layer was extracted with aliquots of EtOAc and the combined organic layers were then washed with a saturated solution of brine, dried over $MgSO_4$ and filtered. After removal of the solvent *in vacuo*, the crude product was adsorbed onto silica and purified by flash chromatography. Pure fractions were evaporated to dryness to afford the desired compound as a yellow foam solid.

Yield: 91% (335.0 mg, 0.5 mmol). **^{1}H NMR** (500 MHz, $CDCl_3$) δ 8.51 (s, 1H), 7.88 (d, *J* = 2.1 Hz, 1H), 7.32 (dd, *J* = 8.7, 2.1 Hz, 1H), 7.28 (d, *J* = 8.6 Hz, 2H), 6.92 (d, *J* = 8.7 Hz, 1H), 6.87 (d, *J* = 8.6 Hz, 2H), 5.51 (s, 2H), 4.23 (t, *J* = 4.8 Hz, 2H), 4.04 (s, 3H), 3.79 (t, *J* = 4.8 Hz, 2H), 3.66 (t, *J* = 8.5 Hz, 2H), 3.46 (s, 3H), 3.40 (s, 4H), 2.76 (s, 4H), 2.49 (s, 3H), 0.94 (t, *J* = 8.5 Hz, 2H), -0.04 (s, 9H). **^{13}C NMR** (126 MHz, $CDCl_3$) δ 163.03, 153.07, 151.25, 150.64, 139.43, 137.09, 136.46, 132.45, 129.37, 128.36, 127.17, 116.65, 115.56, 114.02, 111.46, 104.08, 70.95, 70.77, 69.52, 66.80, 59.67, 54.71, 54.52, 53.86, 47.62, 18.10, -1.27. **LC/MS:** *m/z* calc. for $[C_{33}H_{44}N_6O_6Si+H]^+$ 649.32, found 649.58.

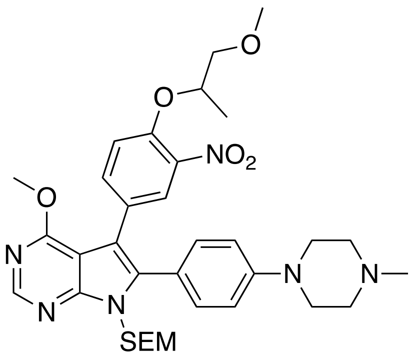

4-Methoxy-5-{4-[(1-methoxypropan-2-yl)oxy]-3-nitrophenyl}-6-[4-(4-methylpiperazin-1-yl)phenyl]-7-{[2-(trimethylsilyl)ethoxy]methyl}-7*H*-pyrrolo[2,3-*d*]pyrimidine

A Schlenk vessel was evacuated and filled with Ar three times. To this was added a mixture of dioxane:H_2O (3:1), *via* syringe, under positive pressure Ar. The solvent was degassed using 3-5 cycles of freeze-pump-thaw technique and subsequently degassed for 15 min by Ar purge. Another Schlenk vessel was charged with 6-Bromo-4-methoxy-5-{4-[(1-methoxypropan-2-yl)oxy]-3-nitrophenyl}-7-{[2-(trimethylsilyl)ethoxy]methyl}-7*H*-pyrrolo[2,3-*d*]pyrimidine (240.0 mg, 0.4 mmol, 1.0 eq.), 4-(4-methylpiperazin-1-yl)phenylboronic acid pinacol ester (153.4 mg, 0.5 mmol, 1.2 eq.), $Pd(PPh_3)_4$ (92.4 mg, 0.1 mmol, 0.2 eq.) and Na_2CO_3 (106.0 mg, 1.0 mmol, 2.5 eq.) and evacuated and filled with Ar three times. The degassed solvent was added to this Schlenk *via* syringe under positive pressure Ar (3 mL). The sealed reaction mixture was then heated to 80 °C and stirred overnight (16 h), during which time the reaction mixture changed to a reddish brown colour. The reaction mixture was cooled to rt, filtered through Celite, diluted with EtOAc and water and the organic layer was separated. The aqueous layer was extracted with aliquots of EtOAc and the combined organic layers were then washed with a saturated solution of brine, dried over $MgSO_4$ and filtered. After removal of the solvent *in vacuo*, the crude product was adsorbed onto silica and purified by flash chromatography. Pure fractions were evaporated to dryness to afford the desired compound as a yellow foamy solid.

Yield: 94% (264.0 mg, 0.4 mmol). **^{1}H NMR** (500 MHz, $CDCl_3$) δ 8.52 (s, 1H), 7.83 (d, *J* = 1.9 Hz, 1H), 7.31 – 7.29 (m, 2H), 6.97 (d, *J* = 8.8 Hz, 1H), 6.90 – 6.87 (m, 3H), 5.51 (s, 2H), 4.64 – 4.60 (m, 1H), 4.05 (s, 3H), 3.67 – 3.61 (m, 3H), 3.53–3.50 (m, 1H), 3.41 (s, 3H), 3.37 (s, 4H), 2.54 (br s, 4H), 2.50 (s, 3H), 1.36 (d, *J* = 6.3 Hz, 3H), 0.94 (t, *J* = 8.4 Hz, 2H), -0.04 (s, 9H). **^{13}C NMR** (126 MHz, $CDCl_3$) δ 163.03, 153.07, 151.24, 149.84, 140.44, 137.03, 136.12, 132.48, 129.39, 128.15, 127.00, 120.68, 116.70, 115.60, 111.54, 104.06, 76.05, 76.01, 70.94, 66.80, 59.60, 54.67, 53.87, 48.41, 47.60, 18.11, 17.02, -1.27. **LC/MS:** *m/z* calc. for $[C_{34}H_{46}N_6O_6Si+H]^+$ 663.33, found 663.58.

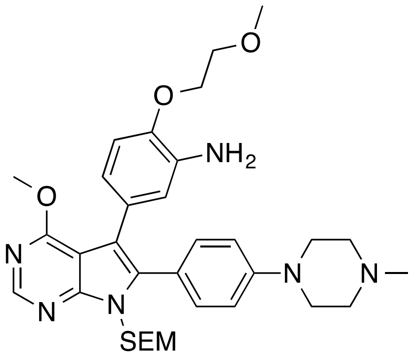

5-{4-Methoxy-6-[4-(4-methylpiperazin-1-yl)phenyl]-7-{[2-(trimethylsilyl)ethoxy]methyl}-7*H*-pyrrolo[2,3-*d*]pyrimidin-5-yl}-2-(2-methoxyethoxy)aniline

A suspension of 4-methoxy-5-[4-(2-methoxyethoxy)-3-nitrophenyl]-6-[4-(4-methylpiperazin-1-yl)phenyl]-7-{[2-(trimethylsilyl)ethoxy]methyl}-7*H*-pyrrolo[2,3-*d*]pyrimidine (350.0 mg, 0.5 mmol, 1.0 eq.), iron (150.6 mg, 2.7 mmol, 5.0 eq.) and NH_4Cl (288.5 mg, 5.4 mmol, 10.0 eq.) in a mixture of EtOH:H_2O (1:1) was sonicated at 60 °C for 3 h, during which time the reaction mixture became a chocolate brown colour. The reaction mixture was cooled to rt, filtered through Celite and washed with MeOH. After removal of the solvent *in vacuo,* the remaining residue was diluted with CH_2Cl_2 and water and the organic layer was separated. The aqueous layer was extracted with aliquots of CH_2Cl_2 and the combined organic layers were then washed with a saturated solution of NaCl, dried over $MgSO_4$ and filtered. After removal of the solvent *in vacuo*, the crude product was

adsorbed onto silica and purified by flash chromatography, which afforded the pure compound as a white foamy solid.

Yield: 84% (334.0 mg, 0.5 mmol). **^{1}H NMR** (400 MHz, $CDCl_3$) δ 8.48 (s, 1H), 7.29 (d, *J* = 8.8 Hz, 2H), 6.84 (d, *J* = 8.8 Hz, 2H), 6.69 (d, *J* = 2.0 Hz, 1H), 6.67 (d, *J* = 8.3 Hz, 1H), 6.58 (dd, *J* = 8.3, 2.0 Hz, 1H), 5.50 (s, 2H), 4.13 (t, *J* = 5.0 Hz, 2H), 4.00 (s, 3H), 3.75 (t, *J* = 4.8 Hz, 2H), 3.65 (t, *J* = 8.4 Hz, 2H), 3.44 (s, 3H), 3.33 (s, 4H), 2.70 (s, 4H), 2.44 (s, 3H), 0.93 (t, *J* = 8.4 Hz, 2H), -0.05 (s, 9H). **^{13}C NMR** (101 MHz, $CDCl_3$) δ 163.21, 152.98, 150.73, 150.37, 145.26, 136.15, 135.71, 132.39, 127.43, 121.59, 118.51, 115.39, 115.32, 114.13, 111.61, 104.60, 71.38, 70.95, 67.98, 66.61, 59.27, 54.87, 53.75, 47.98, 45.78, 18.13, -1.28. **LC/MS:** *m/z* calc. for $[C_{33}H_{46}N_6O_4Si+H]^+$ 619.34, found 619.44.

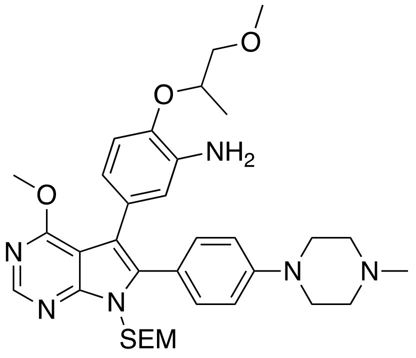

5-{4-Methoxy-6-[4-(4-methylpiperazin-1-yl)phenyl]-7-{[2-(trimethylsilyl)ethoxy]methyl}-7*H*-pyrrolo[2,3-*d*]pyrimidin-5-yl}-2-(1-methoxypropan-2-yl)aniline

A suspension of 4-Methoxy-5-{4-[(1-methoxypropan-2-yl)oxy]-3-nitrophenyl}-6-[4-(4-methylpiperazin-1-yl)phenyl]-7-{[2-(trimethylsilyl)ethoxy]methyl}-7*H*-pyrrolo[2,3-*d*]pyrimidine (280.0 mg, 0.4 mmol, 1.0 eq.), iron (118.0 mg, 2.1 mmol, 5.0 eq.) and NH_4Cl (226.0 mg, 4.2 mmol, 10.0 eq.) in a mixture of EtOH:H_2O (1:1) was sonicated at 60 °C for 3 h, during which time the reaction mixture became a chocolate brown color. The reaction mixture was cooled to rt, filtered through Celite and washed with MeOH. After removal of the solvent *in vacuo,* the residue was

diluted with CH_2Cl_2 and water and the organic layer was separated. The aqueous layer was extracted with aliquots of CH_2Cl_2 and the combined organic layers were then washed with a saturated solution of NaCl, dried over $MgSO_4$ and filtered. After removal of the solvent *in vacuo*, the crude product was adsorbed onto silica and purified by flash chromatography, which afforded the pure compound as a white foamy solid.

Yield: 84% (225.1 mg, 0.4 mmol). **^{1}H NMR** (600 MHz, $CDCl_3$) δ 8.46 (s, 1H), 7.28 (s, 1H), 7.27 (s, 1H), 6.83 (s, 1H), 6.81 (s, 1H), 6.70 (d, *J* = 8.2 Hz, 1H), 6.68 (d, *J* = 1.8 Hz, 1H), 6.55 (dd, *J* = 8.2, 1.8 Hz, 1H), 5.48 (s, 2H), 4.42–4.38 (m, 1H), 3.99 (s, 3H), 3.80 (br s, 2H), 3.62 (t, *J* = 8.4 Hz, 2H), 3.59–3.55 (m, 1H), 3.46–3.43 (m, 1H), 3.39 (s, 3H), 3.25 (br s, 4H), 2.59 (br s, 4H), 2.36 (s, 3H), 1.30 (d, *J* = 6.3 Hz, 3H), 0.91 (t, *J* = 8.4 Hz, 2H), -0.07 (s, 9H). **^{13}C NMR** (151 MHz, $CDCl_3$) δ 163.09, 152.89, 150.61, 150.53, 144.02, 136.91, 136.16, 132.29, 127.80, 121.41, 121.20, 118.69, 115.08, 114.65, 114.00, 104.50, 76.19, 74.39, 70.87, 66.51, 59.30, 54.97, 53.65, 48.15, 46.00, 18.04, 17.33, -1.35. **LC/MS:** *m/z* calc. for $[C_{34}H_{48}N_6O_4Si+H]^+$ 633.36, found 633.31.

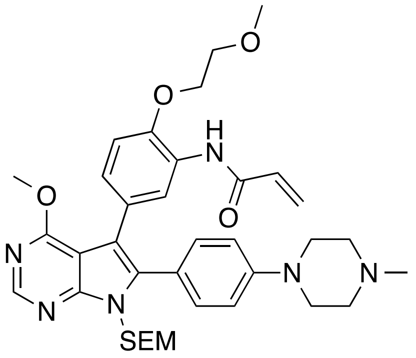

***N*-{5-[4-Methoxy-6-[4-(4-methylpiperazin-1-yl)phenyl]-7-{[2-(trimethylsilyl)ethoxy]methyl}-7*H*-pyrrolo[2,3-*d*]pyrimidin-5-yl]-2-(2-methoxyethoxy)phenyl}acrylamide**

To a suspension of 5-{4-methoxy-6-[4-(4-methylpiperazin-1-yl)phenyl]-7-{[2-(trimethylsilyl)ethoxy]methyl}-7*H*-pyrrolo[2,3-*d*]pyrimidin-5-yl)-2-(2-methoxyethoxy)aniline (200.0 mg, 0.3 mmol, 1.0 eq.) and K_2CO_3 (98.3 mg, 0.7 mmol, 2.2 eq.) in THF, cooled to 0 °C,

was added a solution of acryloyl chloride (31.6 µL, 0.4 mmol, 1.2 eq.) in THF drop-wise over 15 min (1 mL). The reaction was allowed to warm to rt and stirred overnight (12 h). The reaction mixture was then diluted with EtOAc and water and the organic layer was separated. The aqueous layer was extracted with aliquots of EtOAc and the combined organic layers were then washed with a 1 M solution of HCl, a saturated solution of $NaHCO_3$, a saturated solution of NaCl, dried over $MgSO_4$ and filtered. After removal of the solvent *in vacuo*, the crude product was adsorbed onto silica and purified by flash chromatography. Pure fractions were evaporated to dryness to afford the desired compound as a white solid.

Yield: 79% (172.0 mg, 0.3 mmol). **^{1}H NMR** (600 MHz, $CDCl_3$) δ 8.63 (s, 1H), 8.51 (s, 1H), 8.22 (s, 1H), 7.33 (d, *J* = 8.7 Hz, 2H), 6.87 (d, *J* = 8.8 Hz, 2H), 6.83 (d, *J* = 9.0 Hz, 1H), 6.76 (d, *J* = 8.3 Hz, 1H), 6.38 (dd, *J* = 16.8, 1.0 Hz, 1H), 6.29 (dd, *J* = 16.8, 10.2 Hz, 1H), 5.72 (d, *J* = 10.2 Hz, 1H), 5.53 (s, 2H), 4.16 (t, *J* = 4.7 Hz, 2H), 4.04 (s, 3H), 3.73 (t, *J* = 4.7 Hz, 2H), 3.66 (t, *J* = 8.1 Hz, 2H), 3.46 (s, 3H), 3.27 (s, 4H), 2.60 (s, 4H), 2.37 (s, 3H), 0.97 (t, *J* = 8.7 Hz, 2H), -0.01 (s, 9H). **^{13}C NMR** (151 MHz, $CDCl_3$) δ 163.17, 152.78, 150.70, 150.62, 145.71, 136.41, 132.33, 131.97, 129.10, 128.30, 126.66, 123.39, 120.91, 119.75, 116.07, 115.08, 113.76, 112.74, 104.52, 70.90, 69.38, 66.42, 59.05, 54.96, 53.56, 48.93, 48.20, 46.03, 17.98, -1.37. **LC/MS:** *m/z* calc. for $[C_{36}H_{48}N_6O_5Si+H]^+$ 673.35, found 673.47.

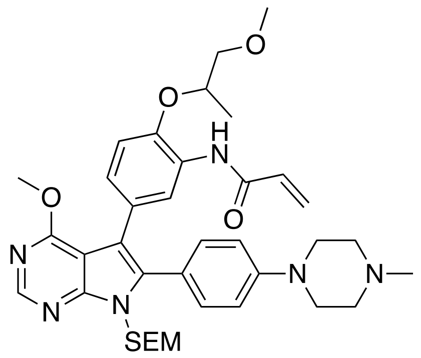

***N*-(5-{4-Methoxy-6-[4-(4-methylpiperazin-1-yl)phenyl]-7-{[2-(trimethylsilyl)ethoxy]methyl}-*7H*-pyrrolo[2,3-*d*]pyrimidin-5-yl}-2-(1-methoxypropan-2-yl)phenyl)acrylamide**

To a suspension of 5-{4-Methoxy-6-[4-(4-methylpiperazin-1-yl)phenyl]-7-{[2-(trimethylsilyl)ethoxy]methyl}-7*H*-pyrrolo[2,3-*d*]pyrimidin-5-yl}-2-(1-methoxypropan-2-yl)aniline (100 mg, 0.2 mmol, 1.0 eq.) and K_2CO_3 (48.0 mg, 0.3 mmol, 2.2 eq.) in THF, cooled to 0 °C, was added a solution of acryloyl chloride (15.5 µL, 0.2 mmol, 1.2 eq.) in THF drop-wise over 15 min (1 mL). The reaction was allowed to warm to rt and stirred overnight (12 h). The reaction mixture was then diluted with EtOAc and water and the organic layer was separated. The aqueous layer was extracted with aliquots of EtOAc and the combined organic layers were then washed with a 1 M solution of HCl, a saturated solution of $NaHCO_3$, a saturated solution of NaCl, dried over $MgSO_4$ and filtered. After removal of the solvent *in vacuo*, the crude product was adsorbed onto silica and purified by flash chromatography. Pure fractions were evaporated to dryness to afford the desired compound as a white solid.

Yield: 75% (81.2 mg, 0.1 mmol). **^{1}H NMR** (600 MHz, $CDCl_3$) δ 8.63 (s, 1H), 8.54 (s, 1H), 8.47 (s, 1H), 7.30 (s, 1H), 7.29 (s, 1H), 6.84 (s, 1H), 6.82 (s, 1H), 6.80–6.76 (m, 2H), 6.35 (dd, *J* = 16.9, 0.9 Hz, 1H), 6.25 (dd, *J* = 16.9, 10.2 Hz, 1H), 5.68 (d, *J* = 10.2 Hz, 1H), 5.49 (s, 2H), 4.30–4.23 (m, 1H), 3.62 (t, *J* = 8.4 Hz, 2H), 3.57–3.53 (m, 1H), 3.49–3.46 (m, 1H), 3.43 (s, 3H), 3.26 (br s, 4H), 2.60 (br s, 4H), 2.36 (s, 3H), 1.32 (d, *J* = 6.3 Hz, 3H), 0.93 (t, *J* = 8.4 Hz, 2H), -0.06 (s, 9H). **^{13}C NMR** (151 MHz, $CDCl_3$) δ 163.26, 163.16, 152.86, 150.80, 150.62, 145.01, 136.47, 132.45,

132.01, 129.82, 129.27, 126.71, 126.38, 123.62, 121.15, 116.82, 115.23, 113.91, 104.58, 77.04, 76.04, 70.85, 66.50, 59.31, 54.97, 53.65, 48.22, 46.01, 18.06, 17.14, -1.31. **LC/MS:** *m/z* calc. for $[C_{37}H_{50}N_6O_5Si+H]^+$ 687.37, found 687.41.

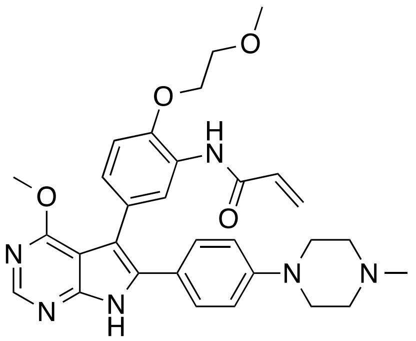

N-(5-{4-Methoxy-6-[4-(4-methylpiperazin-1-yl)phenyl]-7H-pyrrolo[2,3-d]pyrimidin-5-yl}-2-(2-methoxyethoxy)phenyl)acrylamide (20a)

To a solution of *N*-{5-(4-methoxy-6-[4-(4-methylpiperazin-1-yl)phenyl]-7-{[2-(trimethylsilyl) ethoxy]methyl}-7*H*-pyrrolo[2,3-*d*]pyrimidin-5-yl)-2-(2-methoxyethoxy)phenyl}acrylamide (171.2 mg, 0.3 mmol) in CH_2Cl_2 (15 mL) was added TFA (5 mL). The reaction mixture was allowed to stir for 3 h during which a brown color change was witnessed. After removal of the solvent *in vacuo*, the crude residue was re-dissolved in THF (10 mL) and an aqueous solution of 1 M KOH (10 mL) and allowed to stir for a further 2 h, resulting in a clear solution. The reaction mixture was then neutralized by the slow addition of an aqueous solution of 1 M HCl (10 mL), diluted with CH_2Cl_2 (30 mL) and water (20 mL) and the organic layer was separated. The aqueous layer was extracted with aliquots of CH_2Cl_2 and the combined organic layers were then washed with a saturated solution of $NaHCO_3$, a saturated solution of NaCl, dried over $MgSO_4$ and filtered. After removal of the solvent *in vacuo*, the crude product was adsorbed onto silica and purified by flash chromatography. Pure fractions were evaporated to dryness to afford the desired compound as a white solid.

Yield: 72% (99.0 mg, 0.2 mmol). **^{1}H NMR** (500 MHz, $CDCl_3$) δ 13.05 (s, 1H), 8.61 (s, 1H), 8.31 (s, 1H), 8.20 (s, 1H), 7.34 (d, *J* = 8.5 Hz, 2H), 6.92 (d, *J* = 8.0 Hz, 1H), 6.80 (d, *J* = 8.9 Hz, 2H), 6.78 (d, *J* = 8.7 Hz, 1H), 6.31 (d, *J* = 16.8 Hz, 1H), 6.23 (dd, *J* = 16.8, 9.9 Hz, 1H), 5.66 (d, *J* = 9.9 Hz, 1H), 4.14 (t, *J* = 4.8 Hz, 2H), 3.93 (s, 3H), 3.69 (t, *J* = 4.8 Hz, 2H), 3.41 (s, 3H), 3.21 (s, 4H), 2.55 (s, 4H), 2.32 (s, 3H). **^{13}C NMR** (126 MHz, $CDCl_3$) δ 163.40, 163.21, 152.23, 150.38, 149.88, 146.09, 133.94, 131.98, 129.51, 128.97, 128.50, 126.98, 126.69, 123.52, 122.96, 115.50, 113.08, 111.18, 106.22, 71.03, 69.51, 59.22, 54.96, 53.70, 48.17, 46.03. **HRMS:** *m/z* calc. for $[C_{30}H_{34}N_6O_4+H]^+$ 543.27143, found 543.27285, calc. for $[C_{30}H_{34}N_6O_4+Na]^+$ 565.25337, found 565.25505.

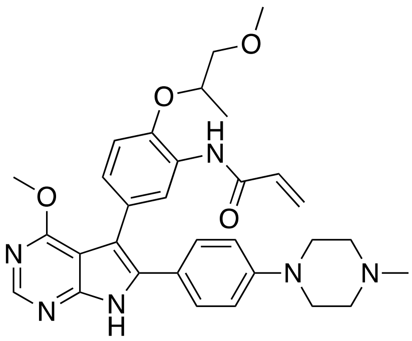

***N*-[5-{4-Methoxy-6-[4-(4-methylpiperazin-1-yl)phenyl]-7*H*-pyrrolo[2,3-*d*]pyrimidin-5-yl}-2-(1-methoxypropan-2-yl)phenyl]acrylamide (20b)**

To a solution of *N*-[5-{4-methoxy-6-[4-(4-methylpiperazin-1-yl)phenyl]-7-{[2-(trimethylsilyl)ethoxy]methyl}-7*H*-pyrrolo[2,3-*d*]pyrimidin-5-yl}-2-(1-methoxypropan-2-yl)phenyl]acrylamide (81.0 mg, 0.1 mmol) in CH_2Cl_2 (15 mL) was added TFA (5 mL). The reaction mixture was allowed to stir for 3 h during which a brown color change was witnessed. After removal of the solvent *in vacuo*, the crude residue was re-dissolved in THF (10 mL) and an aqueous solution of 1 M KOH (10 mL) and allowed to stir for a further 2 h, resulting in a clear solution. The reaction mixture was then neutralized by the slow addition of an aqueous solution of

1 M HCl (10 mL), diluted with CH_2Cl_2 (30 mL) and water (20 mL) and the organic layer was separated. The aqueous layer was extracted with aliquots of CH_2Cl_2 and the combined organic layers were then washed with a saturated solution of $NaHCO_3$, a saturated solution of NaCl, dried over $MgSO_4$ and filtered. After removal of the solvent *in vacuo*, the crude product was adsorbed onto silica and purified by flash chromatography. Pure fractions were evaporated to dryness to afford the desired compound as a white solid.

Yield: 79% (52.1 mg, 0.1 mmol). **^{1}H NMR** (500 MHz, $CDCl_3$) δ 13.28 (s, 1H), 8.75 (s, 1H), 8.66 (s, 1H), 8.40 (s, 1H), 7.45 (s, 1H), 7.43 (s, 1H), 6.99 (d, *J* = 8.0 Hz, 1H), 6.92 (d, *J* = 8.3 Hz, 1H), 6.88 (s, 1H), 6.41 (d, *J* = 16.9 Hz, 1H), 6.31 (dd, *J* = 16.9, 10.1 Hz, 1H), 5.73 (d, *J* = 10.1 Hz, 1H), 4.38–4.32 (m, 1H), 4.02 (s, 3H), 3.64–3.59 (m, 1H), 3.56–3.52 (m, 1H), 3.48 (s, 3H), 3.29 (br s, 4H), 2.63 (br s, 4H), 2.39 (s, 3H), 1.39 (d, *J* = 6.3 Hz, 3H). **^{13}C NMR** (126 MHz, $CDCl_3$) δ 163.36, 163.26, 152.23, 150.40, 149.83, 145.28, 134.01, 131.92, 130.17, 129.92, 129.54, 126.91, 126.56, 123.64, 122.92, 117.27, 115.44, 111.17, 106.17, 76.03, 59.33, 54.96, 53.66, 48.18, 46.04, 17.13. **HRMS:** *m/z* calc. for $[C_{31}H_{36}N_6O_4+H]^+$ 557.28708, found 557.28870, calc. for $[C_{31}H_{36}N_6O_4+Na]^+$ 579.26902, found 579.27084.

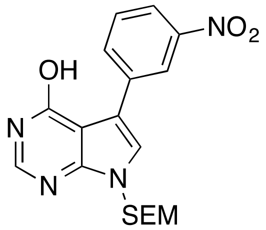

5-(3-Nitrophenyl)-7-{[2-(trimethylsilyl)ethoxy]methyl}-7*H*-pyrrolo[2,3-*d*]pyrimidin-4-ol (23)

A solution of compound **12** (2.9 g, 7.1 mmol, 1.0 eq.), K_2CO_3 (2.0 g, 14.2 mmol, 2.0 eq.) and *m*-nitrobenzeneboronic acid (1.4 g, 8.5 mmol, 1.2 eg) in MeCN:H_2O (30:15 mL) was degassed with Ar for 15 min. $Pd(PPh_3)_4$ (321.0 mg, 0.7 mmol, 0.1 eq.) was then added and the sealed vial heated to 150 °C for 90 min in a microwave reactor. The reaction mixture was filtered through

Celite, concentrated *in vacuo*, taken in EtOAc, washed with a saturated solution of NaCl and dried over Na_2SO_4. After removal of the solvent *in vacuo*, the crude product was adsorbed onto silica and purified by column chromatography. Pure fractions were evaporated to dryness to afford the desired compound as a yellow solid.

Yield: 69% (1.9 g, 4.9 mmol). **^{1}H NMR** (500 MHz, $CDCl_3$) δ 12.54 (s, 1H), 9.03 (s, 1H), 8.21 (d, *J* = 7.7 Hz, 1H), 8.14 (dd, *J* = 8.0, 1.5 Hz, 1H), 8.04 (s, 1H), 7.57 (t, *J* = 7.7 Hz, 1H), 7.37 (s, 1H), 5.62 (s, 2H), 3.61 (t, *J* = 8.4 Hz, 2H), 0.97 (t, *J* = 8.4 Hz, 2H), -0.01 ppm (s, 9H). **^{13}C NMR** (126 MHz, DMSO-d_6) δ 158.5, 149.2, 144.7, 135.3, 133.8, 133.1, 132.0, 129.5, 123.4, 122.4, 120.7, 104.8, 73.0, 65.7, 17.1, -1.4 ppm. **HRMS**: *m/z* calc. for $[C_{18}H_{22}O_4N_4Si+H]^+$ 387.14831, found 387.14953.

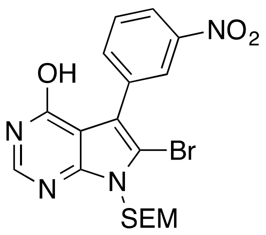

6-Bromo-5-(3-nitrophenyl)-7-{[2-(trimethylsilyl)ethoxy]methyl}-7*H*-pyrrolo[2,3-*d*]pyrimidin-4-ol (24)

Compound **23** (1.2 g, 3.1 mmol, 1.0 eq.) was dissolved in MeCN (70 mL), *N*-bromosuccinimide (1.1 g, 6.1 mmol, 2.0 eq.) added and stirred at rt for 90 min. The reaction mixture was poured into water, saturated solution of NaCl added and extracted with EtOAc. The combined organic layers were washed with aqueous solution of $Na_2S_2O_3$, NaOH (1 M), saturated solution of NaCl, dried over Na_2SO_4 and the solvent removed *in vacuo*. The desired product was isolated without further purification as a brown solid.

Yield: quant. (1.5 g, 3.1 mmol). **^{1}H NMR** (500 MHz, DMSO-d_6) δ 12.28 (s, 1H), 8.40–8.35 (m, 1H), 8.21 (ddd, *J* = 8.3, 2.3, 1.0 Hz, 1H), 8.06 (d, *J* = 3.8 Hz, 1H), 8.02–7.98 (m, 1H), 7.74 (t, *J* = 8.0

Hz, 1H), 5.61 (s, 2H), 3.60 (t, J = 8.0 Hz, 2H), 0.86 (t, J = 7.9 Hz, 2H), -0.06 ppm (s, 9H). **^{13}C NMR** (126 MHz, DMSO-d_6) δ 179.32, 156.94, 147.22, 145.29, 136.79, 133.48, 133.12, 131.98, 129.18, 124.78, 121.87, 117.13, 107.84, 105.68, 71.95, 65.84, 17.07, -1.42 ppm. **HRMS**: *m/z* calc. for $[C_{18}H_{21}O_4N_4BrSi+H]^+$ 465.05882, found 465.05882, calc. for $[C_{18}H_{21}O_4N_4{}^{81}BrSi+H]^+$ 467.05677, found 467.05671.

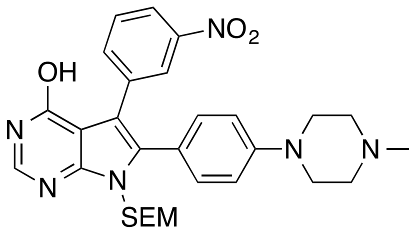

6-[4-(4-Methylpiperazin-1-yl)phenyl]-5-(3-nitrophenyl)-7-{[2-(trimethylsilyl)ethoxy]methyl}-7*H*-pyrrolo[2,3-*d*]pyrimidin-4-ol (25)

A solution of compound **24** (3.3 g, 7.0 mmol, 1.0 eq.), K_2CO_3 (1.9 g, 14.0 mmol, 2.0 eq.) and 4-(4-methylpiperazin-1-yl)phenylboronic acid pinacol ester (2.5 g, 8.4 mmol, 1.2 eq.) in DME:H_2O (4:1, 60 mL) was degassed with Ar for 15 min. Then, $Pd(PPh_3)_4$ (0.8 g, 0.7 mmol, 0.1 eq.) was added and the sealed vial heated to 150 °C for 90 min in a microwave reactor. The reaction mixture was filtered through Celite, concentrated *in vacuo*, taken in CH_2Cl_2:MeOH (20:1, 30 mL), washed with a saturated solution of NaCl and dried over Na_2SO_4. After removal of the solvent *in vacuo*, the crude product was adsorbed onto silica and purified by column chromatography. Pure fractions were evaporated to dryness to afford the desired compound as a yellow solid.

Yield: 56% (2.2 g, 3.9 mmol). **^{1}H NMR** (500 MHz, DMSO-d_6) δ 12.13 (s, 1H), 8.23–8.11 (m, 1H), 8.04 (s, 1H), 8.03–7.98 (m, 1H), 7.61 (d, J = 7.8 Hz, 1H), 7.47 (t, J = 8.0 Hz, 1H), 7.18 (d, J = 8.8 Hz, 2H), 6.93 (d, J = 8.8 Hz, 2H), 5.38 (s, 2H), 3.48 (t, J = 8.0 Hz, 2H), 3.20–3.16 (m, 4H), 2.45–2.40 (m, 4H), 2.21 (s, 3H), 0.79 (t, J = 8.0 Hz, 2H), -0.09 ppm (s, 9H). **^{13}C NMR** (126 MHz, DMSO-d_6) δ 157.90, 150.68, 148.42, 147.04, 144.37, 136.91, 135.25, 134.22, 131.79, 128.60, 125.24,

120.62, 118.44, 114.71, 114.49, 105.13, 70.53, 65.70, 54.41, 47.13, 45.69, 24.91, 24.64, 17.28, -1.46 ppm. **HRMS**: *m/z* calc. for $[C_{29}H_{36}O_4N_6Si+H]^+$ 561.26401, found: 561.26498.

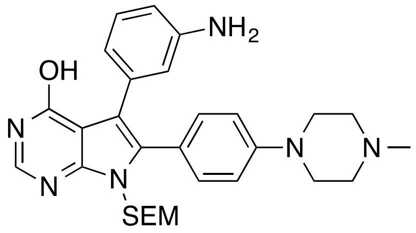

5-(3-Aminophenyl)-6-[4-(4-methylpiperazin-1-yl)phenyl]-7-{[2-(trimethylsilyl)ethoxy]methyl}-7*H*-pyrrolo[2,3-*d*]pyrimidin-4-ol (26)

A suspension of compound **25** (1.3 g, 2.3 mmol, 1.0 eq.), NH_4Cl (1.1 g, 20.9 mmol, 9.0 eq.) and iron (388 mg, 7.0 mmol, 3.0 eq.) in a mixture of EtOH:H_2O (4:1, 21 mL) was stirred at 80 °C for 3 h. The reaction mixture was cooled to rt, filtered through Celite and concentrated *in vacuo*. The residue was diluted with CH_2Cl_2 and water and the organic layer was separated. The aqueous layer was extracted with aliquots of CH_2Cl_2, the combined organic layers dried over Na_2SO_4 and the solvent removed *in vacuo*. The desired product was isolated without further purification as a white solid.

Yield: 94% (1.2 g, 2.2 mmol). **^{1}H NMR** (500 MHz, DMSO-d_6) δ 11.91 (br s, 1H). 7.94 (s, 1H), 7.15 (d, *J* = 8.8 Hz, 2H), 6.87 (d, *J* = 8.8 Hz, 2H), 6.80 (t, *J* = 7.7 Hz, 1H), 6.53 (s, 1H), 6.36 (dd, *J* = 8.0, 1.2 Hz, 1H), 6.30 (d, *J* = 7.7 Hz, 1H), 5.75 (s, 2H), 4.81 (br s, 2H), 3.47 (t, *J* = 8.0 Hz, 2H), 3.16 (t, *J* = 5.0, 4 H), 2.43 (t, *J* = 5.0 Hz, 4H), 2.21 (s, 3H), 0.78 (t, *J* = 8.0 Hz, 2H), -0.08 ppm (s, 9H). **HRMS**: *m/z* calc. for $[C_{29}H_{38}O_2N_6Si+H]^+$ 531.28983, found: 531.29024, calc. for $[C_{29}H_{38}O_2N_6Si+Na]^+$ 553.27177, found 553.27080.

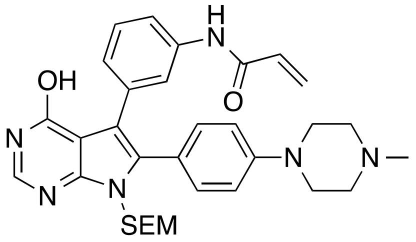

N-(3-{4-Hydroxy-6-[4-(4-methylpiperazin-1-yl)phenyl]-7-{[2-(trimethylsilyl)ethoxy]methyl)}-7*H*-pyrrolo[2,3-*d*]pyrimidin-5-yl}phenyl)acrylamide (27a)

To a solution of compound **26** (1.2 g, 2.3 mmol, 1.0 eq.) and DIPEA (4.0 mL, 23.2 mmol, 10.0 eq.) in THF (28 mL) was added at 0 °C drop-wise a solution of acryloyl chloride (374.7 µL, 4.6 mmol, 2.0 eq.) in THF (2 mL). The reaction was stirred at rt until full conversion, quenched with water and extracted with 5% MeOH in CH_2Cl_2. The combined organic layers were dried over Na_2SO_4 and the solvent removed *in vacuo*. Purification by column chromatography yielded the desired product as yellow solid.

Yield: 76% (1.0 g, 1.8 mmol). **^{1}H NMR** (500 MHz, DMSO-d_6) δ 11.98 (s, 1H), 10.03 (s, 1H), 7.98 (d, *J* = 3.0 Hz, 1H), 7.64 (d, *J* = 8.0 Hz, 1H), 7.52 (s, 1H), 7.15 (d, *J* = 8.6 Hz, 2H), 7.10 (t, *J* = 7.9 Hz, 1H), 6.88 (d, *J* = 8.7 Hz, 2H), 6.80 (d, *J* = 7.6 Hz, 1H), 6.43 (dd, *J* = 17.0, 10.2 Hz, 1H), 6.22 (dd, *J* = 17.0, 1.7 Hz, 1H), 5.71 (d, *J* = 10.2 Hz, 1H), 5.36 (s, 2H), 3.48 (t, *J* = 8.03 Hz, 2H), 3.16 (s, 4H), 2.43 (s, 4H), 2.21 (s, 3H), 0.79 (t, *J* = 8.0 Hz, 2H), -0.08 ppm (s, 9H). **^{13}C NMR** (126 MHz, DMSO-d_6) δ 162.92, 157.75, 150.24, 148.04, 143.93, 138.10, 134.20, 133.07, 131.97, 131.66, 130.40, 127.40, 126.52, 125.88, 122.12, 119.37, 116.90, 114.24, 105.58, 70.51, 65.59, 54.47, 47.12, 45.66, 17.30, -1.44 ppm. **HRMS**: *m/z* calc. for $[C_{32}H_{40}O_3N_6Si+H]^+$ 585.30039, found 585.29912.

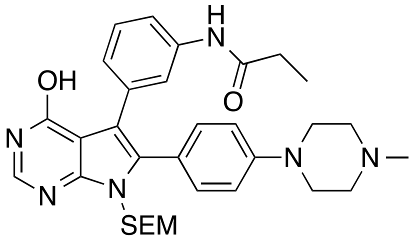

***N*-(3-{4-Hydroxy-6-[4-(4-methylpiperazin-1-yl)phenyl]-7-{[2-(trimethylsilyl)ethoxy]methyl}-7*H*-pyrrolo[2,3-*d*]pyrimidin-5-yl}phenyl)propionamide (27b)**

To a solution of compound **26** (200.0 mg, 0.4 mmol, 1.0 eq.) and DIPEA (0.6 mL, 3.8 mmol, 10 eq.) in THF (9 mL) was added at 0 °C drop-wise a solution of propionyl chloride (39 µL, 0.5 mmol, 1.2 eq.) in THF (1 mL). The reaction was stirred at rt until full conversion, quenched with water and extracted with 5% MeOH in CH_2Cl_2. The combined organic layers were dried over Na_2SO_4 and the solvent removed *in vacuo*. Purification by column chromatography yielded the desired product as white solid.

Yield: 82% (183.0 mg, 0.3 mmol). **^{1}H NMR** (500 MHz, DMSO-d_6) δ 11.97 (s, 1H), 9.74 (s, 1H), 7.98 (s, 1H), 7.55 (d, J = 7.9 Hz, 1H), 7.44 (s, 1H), 7.14 (d, J = 8.7 Hz, 2H), 7.06 (t, J = 7.9 Hz, 1H), 6.87 (d, J = 8.8 Hz, 2H), 6.75 (d, J = 7.6 Hz, 1H), 5.36 (s, 2H), 3.48 (t, J = 8.0 Hz, 2H), 3.16 (t, J = 4.6 Hz, 4H), 2.42 (t, J = 4.6 Hz, 4H), 2.28 (q, J = 7.5 Hz, 2H), 2.21 (s, 3H), 1.05 (t, J = 7.5 Hz, 3H), 0.79 (t, J = 8.0 Hz, 2H), -0.08 ppm (s, 9H). **^{13}C NMR** (126 MHz, DMSO-d_6) δ 171.71, 157.73, 150.22, 148.00, 143.89, 138.47, 134.10, 132.99, 131.65, 127.25, 121.79, 119.45, 117.05, 116.85, 114.75, 114.22, 105.63, 70.51, 65.58, 54.49, 47.14, 45.67, 29.44, 17.30, 9.65, -1.44 ppm. **HRMS**: *m/z* calc. for $[C_{32}H_{42}O_3N_6Si+H]^+$ 587.31604, found 587.31752, calc. for $[C_{32}H_{42}O_3N_6Si+Na]^+$ 609.29799, found 609.29827.

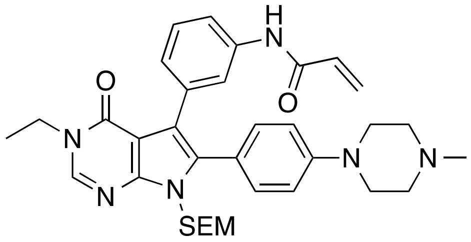

_N_-[3-(3-Ethyl-6-[4-(4-methylpiperazin-1-yl)phenyl]-4-oxo-7-{[2-(trimethylsilyl)ethoxy]methyl}-4,7-dihydro-3*H*-pyrrolo[2,3-*d*]pyrimidin-5-yl)phenyl]acrylamide (28a)

To a solution of compound **27a** (100.0 mg, 171.0 µmol, 1.0 eq.) in THF (3 mL) was added PPh_3 (448.5 mg, 1.7 mmol, 10.0 eq.) and EtOH (120.0 µL, 1.7 mmol, 12.0 eq.) and the reaction mixture sonicated for 5 min. Subsequently, DIAD (335.7 µL, 1.7 mmol, 10.0 eq.) was added and the resulting mixture sonicated until full conversion at 40 °C. Water was then added and the reaction mixture extracted with 5% MeOH in CH_2Cl_2. The combined organic layers were dried over Na_2SO_4 and the solvent removed *in vacuo*. Purification by column chromatography, followed by flash chromatography using a C18 column yielded the desired product as a light yellow solid.

Yield: 44% (47.1 mg, 76.9 µmol). **^{1}H NMR** (600 MHz, DMSO-d_6) δ 10.06 (s, 1H), 8.36 (s, 1H), 7.64 (dd, *J* = 8.2, 0.9 Hz, 1H), 7.51 (s, 1H), 7.15 (d, *J* = 8.9 Hz, 2H), 7.11 (t, *J* = 7.9 Hz, 1H), 6.89 (d, *J* = 8.9 Hz, 2H), 6.79 (d, *J* = 7.7 Hz, 1H), 6.44 (dd, *J* = 17.0, 10.2 Hz, 1H), 6.22 (dd, *J* = 17.0, 2.0 Hz, 1H), 5.72 (dd, *J* = 10.2, 2.0 Hz, 1H), 5.36 (s, 2H), 3.99 (q, *J* = 7.0 Hz, 2H), 3.50–3.46 (m, 2H), 3.17 (s, 4H), 2.47 (s, 4H), 2.24 (s, 3H), 1.24 (t, *J* = 7.0 Hz, 3H), 0.81–0.77 (m, 2H), -0.08 ppm (s, 9H). **^{13}C NMR** (151 MHz, DMSO-d_6) δ 163.00, 156.76, 150.23, 147.51, 146.48, 138.16, 134.23, 133.56, 132.01, 131.71, 127.50, 126.69, 126.12, 122.16, 117.27, 116.84, 114.35, 104.92, 70.46, 65.69, 54.95, 54.38, 47.00, 45.51, 40.70, 17.31, 15.22, -1.38 ppm. **LC/MS**: *m/z* calc. for $[C_{34}H_{44}O_3N_6Si+H]^+$ 613.33, found 613.64.

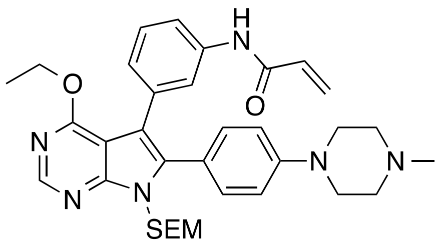

N-[3-(4-ethoxy-6-[4-(4-methylpiperazin-1-yl)phenyl]-7-{[2-(trimethylsilyl)ethoxy]methyl}-7*H*-pyrrolo[2,3-*d*]pyrimidin-5-yl)phenyl]acrylamide (28b)

To a solution of compound **27a** (100.0 mg, 171.0 µmol, 1.0 eq.) in THF (3 mL) was added PPh_3 (448.5 mg, 1.7 mmol, 10.0 eq.) and EtOH (120.0 µL, 1.7 mmol, 12.0 eq.) and the reaction mixture sonicated for 5 min. Subsequently, DIAD (335.7 µL, 1.7 mmol, 10.0 eq.) was added and the resulting mixture sonicated until full conversion at 40 °C. Water was then added and the reaction mixture extracted with 5% MeOH in CH_2Cl_2. The combined organic layers were dried over Na_2SO_4 and the solvent removed *in vacuo*. Purification by column chromatography, followed by flash chromatography using a C18 column yielded the desired product as a light yellow solid.

Yield: 42% (44.1 mg, 72.0 µmol). **^{1}H NMR** (600 MHz, DMSO-d_6) δ 10.06 (s, 1H), 8.46 (s, 1H), 7.70 (s, 1H), 7.57 (d, *J* = 8.1 Hz, 1H), 7.22 (d, *J* = 8.8 Hz, 2H), 7.14 (t, *J* = 7.9 Hz, 1H), 6.92 (d, *J* = 8.9 Hz, 2H), 6.81 (d, *J* = 7.7 Hz, 1H), 6.43 (dd, *J* = 17.0, 10.2 Hz, 1H), 6.23 (dd, *J* = 17.0, 1.9 Hz, 1H), 5.72 (dd, *J* = 10.2, 1.9 Hz, 1H), 5.47 (s, 2H), 4.40 (q, *J* = 7.0 Hz, 2H), 3.50 (t, *J* = 8.1 Hz, 3H), 3.21 (s, 4H), 2.28 (s, 3H), 1.23 (s, 4H), 1.19 (t, *J* = 7.0 Hz, 2H), 0.82–0.78 (m, 2H), -0.10 ppm (s, 9H). **^{13}C NMR** (151 MHz, DMSO-d_6) δ 163.04, 161.98, 152.41, 150.75, 150.38, 138.30, 136.20, 134.20, 131.97, 131.77, 127.59, 126.73, 126.02, 122.16, 119.18, 117.20, 114.36, 113.07, 103.42, 70.50, 69.81, 65.85, 61.80, 54.27, 46.82, 17.31, 13.97, -1.40 ppm. **LC/MS**: *m/z* calc. for $[C_{34}H_{44}O_3N_6Si+H]^+$ 613.33, found 613.44.

General method: Mitsunobu derivatization

To a solution of compound **27a** or **27b** (1.0 eq.) in THF (30 mM) was added PPh_3 (5.0 eq.) and the respective alcohol (5.0 eq.) and the reaction mixture sonicated for 5 min. Subsequently, DIAD (5.0 eq.) was added and the resulting mixture sonicated until full conversion at 40 °C. To this was added water and the mixture was extracted with 5% MeOH in CH_2Cl_2. The combined organic layers were dried over Na_2SO_4 and the solvent removed *in vacuo*. Purification by flash chromatography using a C18 column yielded the intermediate as a light yellow solid. This was subsequently taken up in CH_2Cl_2 (30 mM) and TFA (2 mL) was added. The reaction mixture was then stirred at rt until full conversion (3 h). After removal of the solvent *in vacuo*, the crude residue was re-dissolved in THF (30 mM) and an aqueous solution of NaOH (1 M, 2 mL) was added after which the reaction was allowed to stir for a further 1 h. To this was added CH_2Cl_2 and the mixture was extracted with aliquots of 5% MeOH in CH_2Cl_2. The combined organic layers were dried over Na_2SO_4 and the solvent removed *in vacuo*. Purification by column chromatography or preparative HPLC yielded the desired compound as a white solid.

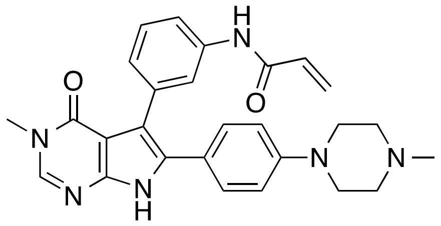

***N*-(3-{3-Methyl-6-[4-(4-methylpiperazin-1-yl)phenyl]-4-oxo-4,7-dihydro-3*H*-pyrrolo[2,3-*d*]pyrimidin-5-yl}phenyl)acrylamide (29a)**

According to the general method, Mitsunobu derivatization, starting from compound **27a** and with methanol as substrate.

Yield: 35% (8.0 mg, 17.1 µmol) over two steps. **^{1}H NMR** (500 MHz, DMSO-d_6) δ 12.11 (s, 1H), 10.08 (s, 1H), 8.18 (s, 1H), 7.73 (d, *J* = 8.2 Hz, 1H), 7.52 (s, 1H), 7.21 (t, *J* = 7.9 Hz, 1H), 7.17 (d, *J* = 9.0 Hz, 2H), 6.93 (d, *J* = 7.8 Hz, 1H), 6.83 (d, *J* = 9.0 Hz, 2H), 6.44 (dd, *J* = 17.0, 10.2 Hz, 1H), 6.23 (dd, *J* = 17.0, 2.0 Hz, 1H), 5.72 (dd, *J* = 10.2, 2.0 Hz, 1H), 3.41 (s, 3H), 3.14 (br s, 4H), 2.45 (br s, 4H), 2.23 ppm (s, 3H). **^{13}C NMR** (126 MHz, DMSO-d_6) δ 148.06, 146.82, 138.88, 135.68, 132.44, 130.75, 128.97, 128.28, 122.30, 117.80, 115.04, 114.83, 106.42, 66.70, 54.85, 47.70, 40.00, 33.55, 26.46, 21.98 ppm. **HRMS**: *m/z* calc. for $[C_{27}H_{28}O_2N_6+H]^+$ 469.23465, found: 469.23495, calc. for $[C_{27}H_{28}O_2N_6+Na]^+$ 491.21660, found: 491.21651.

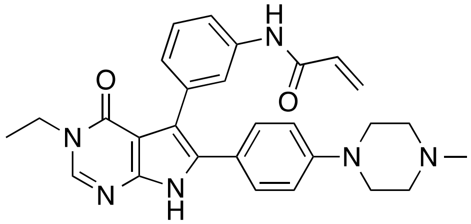

***N*-(3-{3-ethyl-6-[4-(4-methylpiperazin-1-yl)phenyl]-4-oxo-4,7-dihydro-3*H*-pyrrolo[2,3-*d*]pyrimidin-5-yl}phenyl)acrylamide (29b)**

Compound **28a** (20.0 mg, 32.6 µmol, 1.0 eq.) was taken in CH_2Cl_2 (30 mM) and to this was added TFA (2 mL). The reaction mixture was stirred at rt until full conversion (3 h). After removal of the solvent *in vacuo*, the crude residue was re-dissolved in THF (30 mM) and an aqueous solution of NaOH (1 M, 2 mL) and allowed to stir for a further 1 h. To this mixture was added CH_2Cl_2 followed by extraction with aliquots of 5% MeOH in CH_2Cl_2. The combined organic layers were dried over $MgSO_4$ and the solvent removed *in vacuo*. Purification by column chromatography yielded the desired compound as a white solid.

Yield: 75% (11.8 mg, 24.5 µmol). **^{1}H NMR** (500 MHz, DMSO-d_6) δ 12.28 (s, 1H), 10.07 (s, 1H), 8.34 (s, 1H), 7.74 (d, *J* = 8.0 Hz, 1H), 7.65 (s, 1H), 7.29–7.24 (m, 3H), 6.99 (d, *J* = 7.7 Hz, 1H), 6.86 (d, *J* = 8.8 Hz, 2H), 6.45 (dd, *J* = 16.8, 10.3 Hz, 1H), 6.23 (d, *J* = 16.8 Hz, 1H), 5.72 (d, *J* =

10.3 Hz, 1H), 3.94 (q, *J* = 7.3 Hz, 2H), 3.16 (t, *J* = 5.0 Hz, 4H), 2.42 (br s, 4H), 2.21 (s, 3H), 1.21 ppm (d, *J* = 7.3 Hz, 3H). **HRMS**: *m/z* calc. for $[C_{28}H_{30}O_2N_6+H]^+$ 483.25030, found 483.24924.

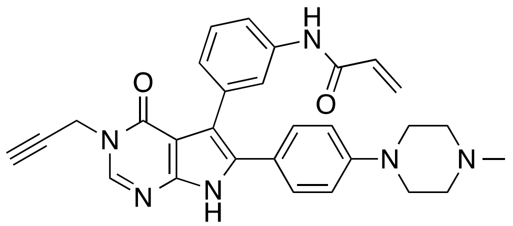

***N*-(3-{6-[4-(4-Methylpiperazin-1-yl)phenyl]-4-oxo-3-(prop-2-yn-1-yl)-4,7-dihydro-3*H*-pyrrolo[2,3-*d*]pyrimidin-5-yl}phenyl)acrylamide (29c)**

According to the general method, Mitsunobu derivatization, starting from compound **27a** and with propargyl alcohol as substrate.

Yield: 79% (33.3 mg, 67.6 µmol) over two steps. **^{1}H NMR** (500 MHz, DMSO-d_6) δ 12.25 (s, 1H), 10.12 (s, 1H), 8.26 (s, 1H), 7.75 (d, *J* = 8.0 Hz, 1H), 7.53 (s, 1H), 7.22 (t, *J* = 7.9 Hz, 1H), 7.17 (d, *J* = 8.8 Hz, 2H), 6.94 (d, *J* = 7.6 Hz, 1H), 6.84 (d, *J* = 8.9 Hz, 2H), 6.45 (dd, *J* = 16.9, 10.2 Hz, 1H), 6.23 (dd, *J* = 16.9, 1.7 Hz, 1H), 5.72 (dd, *J* = 10.2, 1.7 Hz, 1H), 4.74 (d, *J* = 2.0 Hz, 2H), 3.34 (t, *J* = 2.3 Hz, 1H), 3.14 (br s, 4H), 2.43 (br s, 4H), 2.22 ppm (s, 3H). **HRMS**: *m/z* calc. for $[C_{29}H_{28}O_2N_6+H]^+$ 493.23465, found 493.23496, calc. for $[C_{29}H_{28}O_2N_6+Na]^+$ 515.21660, found 515.21650.

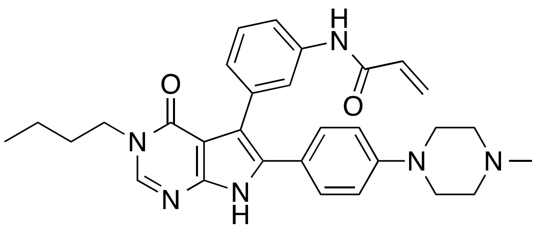

***N*-(3-{3-Butyl-6-[4-(4-methylpiperazin-1-yl)phenyl]-4-oxo-4,7-dihydro-3*H*-pyrrolo[2,3-*d*]pyrimidin-5-yl}phenyl)acrylamide (29d)**

According to the general method, Mitsunobu derivatization, starting from compound **27a** and with butan-1-ol as substrate.

Yield: 42% (19.5 mg, 38.2 µmol) over two steps. **^{1}H NMR** (500 MHz, DMSO-d_6) δ 12.12 (s, 1H), 10.08 (s, 1H), 8.18 (s, 1H), 7.74 (d, J = 8.1 Hz, 1H), 7.51 (s, 1H), 7.22 (t, J = 7.9 Hz, 1H), 7.17 (d, J = 8.9 Hz, 2H), 6.94 (d, J = 7.6 Hz, 1H), 6.83 (d, J = 8.9 Hz, 2H), 6.44 (dd, J = 17.0, 10.2 Hz, 1H), 6.23 (dd, J = 17.0, 1.9 Hz, 1H), 5.72 (dd, J = 10.2, 1.9 Hz, 1H), 3.90 (t, J = 7.2 Hz, 2H), 3.14 (br s, 4H), 2.44 (br s, 4H), 2.22 (s, 3H), 1.65–1.55 (m, 2H), 1.28 (dd, J = 15.0, 7.4 Hz, 2H), 0.89 ppm (t, J = 7.4 Hz, 3H). **^{13}C NMR** (126 MHz, DMSO-d_6) δ 162.98, 157.01, 149.75, 147.38, 146.06, 138.39, 135.20, 131.97, 130.34, 128.50, 127.80, 126.58, 126.24, 121.81, 121.36, 117.33, 114.56, 114.50, 106.07, 54.38, 47.23, 45.53, 45.01, 31.30, 19.28, 13.54 ppm. **HRMS**: *m/z* calc. for $[C_{30}H_{34}O_2N_6+H]^+$ 511.28160, found 511.28168, calc. for $[C_{30}H_{34}O_2N_6+Na]^+$ 533.26355, found 533.26324.

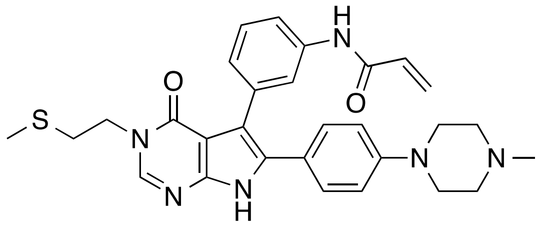

***N*-(3-{6-[4-(4-Methylpiperazin-1-yl)phenyl]-3-[2-(methylthio)ethyl]-4-oxo-4,7-dihydro-3*H*-pyrrolo[2,3-*d*]pyrimidin-5-yl}phenyl)acrylamide (29e)**

According to the general method, Mitsunobu derivatization, starting from compound **27a** and with 2-(methylthio)ethan-1-ol as substrate.

Yield: 14% (4.7 mg, 8.9 µmol) over two steps. **^{1}H NMR** (500 MHz, DMSO-d_6) δ 12.17 (s, 1H), 10.08 (s, 1H), 8.20 (s, 1H), 7.73 (d, J = 8.6 Hz, 1H), 7.51 (s, 1H), 7.22 (t, J = 7.9 Hz, 1H), 7.18 (d, J = 8.8 Hz, 2H), 6.94 (d, J = 7.6 Hz, 1H), 6.85 (d, J = 8.7 Hz, 2H), 6.44 (dd, J = 17.0, 10.2 Hz, 1H), 6.22 (dd, J = 17.0, 1.7 Hz, 1H), 5.72 (dd, J = 10.2, 1.7 Hz, 1H), 4.09 (t, J = 6.7 Hz, 2H), 3.51 (s, 3H), 3.17 (br s, 4H), 2.77 (t, J = 6.7 Hz, 2H), 2.36 (br s, 4H), 2.09 ppm (s, 3H). **HRMS**: *m/z* calc. for $[C_{29}H_{32}O_2N_6S+H]^+$ 529.23802, found 529.23941, calc. for $[C_{29}H_{32}O_2N_6S+Na]^+$ 551.21997, found 551.22131.

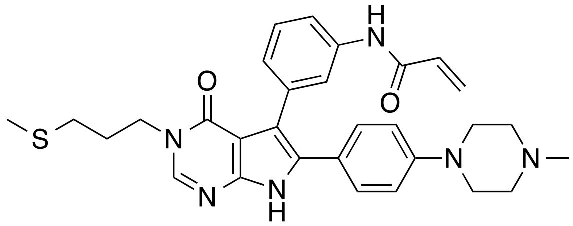

***N*-(3-{6-[4-(4-Methylpiperazin-1-yl)phenyl]-3-[3-(methylthio)propyl]-4-oxo-4,7-dihydro-3*H*-pyrrolo[2,3-*d*]pyrimidin-5-yl}phenyl)acrylamide (29f)**

According to the general method, Mitsunobu derivatization, starting from compound **27a** and with 3-(methylthio)propan-1-ol as substrate.

Yield: 4% (2.6 mg, 4.8 µmol) over two steps. **^{1}H NMR** (500 MHz, DMSO-d_6) δ 12.19 (s, 1H), 10.11 (s, 1H), 8.18 (s, 1H), 7.73 (d, J = 8.2 Hz, 1H), 7.51 (s, 1H), 7.22 (t, J = 7.9 Hz, 1H), 7.16 (d, J = 8.8

Hz, 2H), 6.93 (d, J = 7.7 Hz, 1H), 6.85 (d, J = 8.7 Hz, 2H), 6.44 (dd, J = 17.0, 10.2 Hz, 1H), 6.23 (dd, J = 17.0, 1.7 Hz, 1H), 5.73 (dd, J = 10.2, 1.7 Hz, 1H), 3.97 (t, J = 7.0 Hz, 2H), 3.50 (s, 3H), 3.16 (br s, 4H), 2.48–2.44 (m, 2H), 2.28 (br s, 4H), 2.04 (s, 3H), 1.93–1.85 (m, 2H), 0.85 ppm (t, J = 6.6 Hz, 2H). **HRMS**: *m/z* calc. for $[C_{30}H_{34}O_2N_6S+H]^+$ 543.25367, found 543.25320, calc. for $[C_{30}H_{34}O_2N_6S+Na]^+$ 565.23562, found 565.23510.

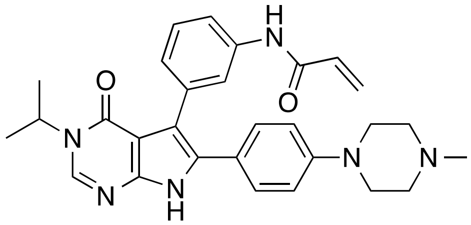

***N*-(3-{3-isopropyl-6-[4-(4-methylpiperazin-1-yl)phenyl]-4-oxo-4,7-dihydro-3*H*-pyrrolo[2,3-*d*]pyrimidin-5-yl}phenyl)acrylamide (29g)**

According to the general method, Mitsunobu derivatization, starting from compound **27a** and with propan-2-ol as substrate.

Yield: 16% (5.6 mg, 11.3 µmol) over two steps. **^{1}H NMR** (500 MHz, DMSO-d_6) δ 12.13 (s, 1H), 10.08 (s, 1H), 8.24 (s, 1H), 7.74 (dd, J = 8.1, 0.9 Hz, 1H), 7.51 (s, 1H), 7.21 (t, J = 7.9 Hz, 1H), 7.17 (d, J = 9.0 Hz, 2H), 6.93 (d, J = 7.8 Hz, 1H), 6.84 (d, J = 9.0 Hz, 2H), 6.44 (dd, J = 17.0, 10.2 Hz, 1H), 6.23 (dd, J = 17.0, 2.0 Hz, 1H), 5.72 (dd, J = 10.2, 2.0 Hz, 1H), 5.04–4.96 (m, 1H), 3.51 (s, 1H), 3.17 (br s, 4H), 2.54 (br s, 3H), 2.30 (br s, 3H), 1.38 (s, 3H), 1.36 ppm (s, 3H). **HRMS**: *m/z* calc. for $[C_{29}H_{32}O_2N_6+H]^+$ 497.26595, found 497.26676.

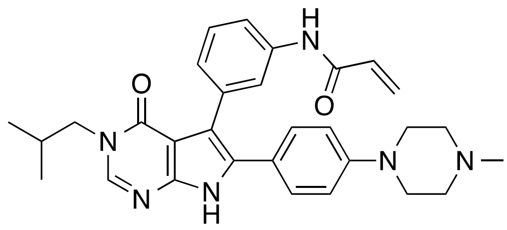

***N*-(3-{3-Isobutyl-6-[4-(4-methylpiperazin-1-yl)phenyl]-4-oxo-4,7-dihydro-3*H*-pyrrolo[2,3-*d*]pyrimidin-5-yl}phenyl)acrylamide (29h)**

According to the general method, Mitsunobu derivatization, starting from compound **27a** and with 2-methylpropan-1-ol as substrate.

Yield: 25% (12.7 mg, 24.8 µmol) over two steps. **^{1}H NMR** (500 MHz, DMSO-d_6) δ 12.13 (s, 1H), 10.07 (s, 1H), 8.13 (s, 1H), 7.73 (d, J = 8.0 Hz, 1H), 7.51 (s, 1H), 7.21 (t, J = 7.9 Hz, 1H), 7.17 (d, J = 8.8 Hz, 2H), 6.94 (d, J = 7.6 Hz, 1H), 6.83 (d, J = 8.8 Hz, 2H), 6.44 (dd, J = 17.0, 10.2 Hz, 1H), 6.23 (dd, J = 17.0, 1.6 Hz, 1H), 5.72 (dd, J = 10.2, 1.6 Hz, 1H), 3.72 (d, J = 7.2 Hz, 2H), 3.15 (br s, 4H), 2.47 (br s, 4H), 2.24 (s, 3H), 2.08–1.98 (m, 1H), 0.86 (s, 3H), 0.84 ppm (s, 3H). **^{13}C NMR** (126 MHz, DMSO-d_6) δ 162.98, 157.19, 149.71, 147.34, 146.38, 138.38, 135.17, 131.97, 130.34, 128.52, 127.79, 126.58, 126.24, 121.83, 121.52, 121.39, 117.33, 114.57, 105.99, 54.31, 52.24, 47.16, 45.42, 27.50, 19.51 ppm. **HRMS**: *m/z* calc. for $[C_{30}H_{34}O_2N_6+H]^+$ 511.28160, found 511.28278, calc. for $[C_{30}H_{34}O_2N_6+Na]^+$ 533.26355, found 533.26445.

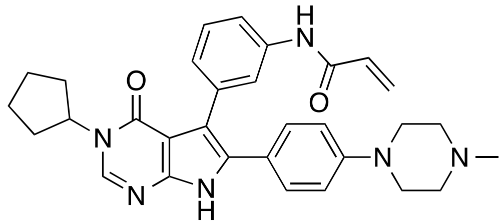

***N*-(3-{3-Cyclopentyl-6-[4-(4-methylpiperazin-1-yl)phenyl]-4-oxo-4,7-dihydro-3*H*-pyrrolo[2,3-*d*]pyrimidin-5-yl}phenyl)acrylamide (29i)**

According to the general method, Mitsunobu derivatization, starting from compound **27a** and with cyclopentanol as substrate.

Yield: 27% (9.2 mg, 17.6 µmol) over two steps. **^{1}H NMR** (500 MHz, DMSO-d_6) δ 12.13 (s, 1H), 10.08 (s, 1H), 8.19 (s, 1H), 7.74 (d, *J* = 8.0 Hz, 1H), 7.51 (s, 1H), 7.22 (t, *J* = 7.9 Hz, 1H), 7.17 (d, *J* = 8.8 Hz, 1H), 6.93 (d, *J* = 7.6 Hz, 1H), 6.84 (d, *J* = 8.9 Hz, 2H), 6.44 (dd, *J* = 17.0, 10.2 Hz, 1H), 6.23 (dd, *J* = 17.0, 1.9 Hz, 1H), 5.72 (dd, *J* = 10.2, 1.9 Hz, 1H), 5.02 (p, *J* = 8.1 Hz, 1H), 3.18 (br s, 4H), 2.58 (s, 3H), 2.33 (br s, 4H), 2.04–1.96 (m, 2H), 1.88–1.76 (m, 4H), 1.67–1.58 ppm (m, 2H). **HRMS**: *m/z* calc. for $[C_{31}H_{34}O_2N_6+H]^+$ 523.28160, found 523.28288, calc. for $[C_{31}H_{34}O_2N_6+Na]^+$ 545.26355, found 545.26461.

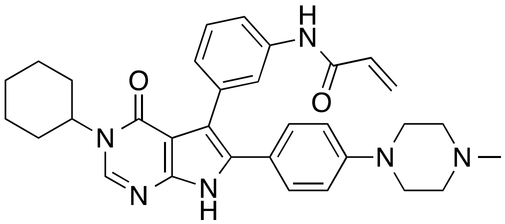

***N*-(3-{3-Cyclohexyl-6-[4-(4-methylpiperazin-1-yl)phenyl]-4-oxo-4,7-dihydro-3*H*-pyrrolo[2,3-*d*]pyrimidin-5-yl}phenyl)acrylamide (29j)**

According to the general method, Mitsunobu derivatization, starting from compound **27a** and with cyclohexanol as substrate.

Yield: 63% (29.1 mg, 54.2 µmol) over two steps. **^{1}H NMR** (500 MHz, DMSO-d_6) δ 12.25 (s, 1H), 10.08 (s, 1H), 8.31 (s, 1H), 7.70 (s, 1H), 7.65 (d, *J* = 8.1 Hz, 1H), 7.31–7.22 (m, 3H), 6.98 (d, *J* = 7.6 Hz, 1H), 6.86 (d, *J* = 8.9 Hz, 2H), 6.43 (dd, *J* = 17.0, 10.2 Hz, 1H), 6.23 (dd, *J* = 17.0, 1.9 Hz, 1H), 5.72 (dd, *J* = 10.2, 1.9 Hz, 1H), 5.23 (dd, *J* = 6.8, 3.4 Hz, 1H), 3.17 (br s, 4H), 2.48 (s, 4H), 2.25 (s, 3H), 1.70 (s, 2H), 1.46–1.35 (m, 4H), 1.26–1.19 ppm (m, 4H). **^{13}C NMR** (126 MHz, DMSO-D6) δ 162.97, 161.39, 152.27, 150.18, 150.01, 138.54, 135.46, 132.74, 131.94, 128.78, 127.89, 126.05, 121.94, 121.06, 118.50, 117.27, 114.52, 112.43, 110.33, 105.29, 102.31, 71.85, 54.25, 47.05, 30.49, 25.06, 22.01 ppm. **HRMS**: *m/z* calc. for $[C_{32}H_{36}O_2N_6+H]^+$ 537.29725, found: 537.29802.

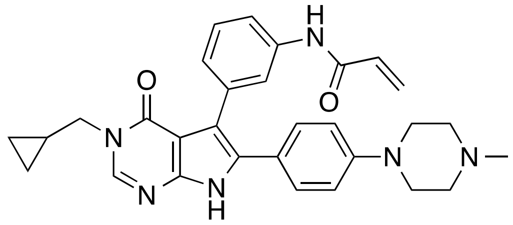

N-(3-{3-(Cyclopropylmethyl)-6-[4-(4-methylpiperazin-1-yl)phenyl]-4-oxo-4,7-dihydro-3H-pyrrolo[2,3-d]pyrimidin-5-yl}phenyl)acrylamide (29k)

According to the general method, Mitsunobu derivatization, starting from compound **27a** and with cyclopropylmethanol as substrate.

Yield: 34% (11.8 mg, 23.3 µmol) over two steps. **^{1}H NMR** (500 MHz, DMSO-d_6) δ 12.13 (s, 1H), 10.07 (s, 1H), 8.20 (s, 1H), 7.73 (d, J = 7.7 Hz, 1H), 7.51 (s, 1H), 7.22 (t, J = 7.9 Hz, 1H), 7.16 (d, J = 8.8 Hz, 2H), 6.95 (d, J = 7.6 Hz, 1H), 6.83 (d, J = 8.9 Hz, 2H), 6.44 (dd, J = 17.0, 10.2 Hz, 1H), 6.23 (dd, J = 17.0, 1.8 Hz, 1H), 5.72 (dd, J = 10.2, 1.8 Hz, 1H), 3.76 (d, J = 7.1 Hz, 2H), 3.13 (t, J = 1.0 Hz, 3H), 2.41 (t, J = 1.0 Hz, 4H), 2.20 (s, 3H), 1.26–1.19 (m, 1H), 0.45 (d, J = 7.9 Hz, 2H), 0.37 ppm (d, J = 4.8 Hz, 2H). **^{13}C NMR** (126 MHz, DMSO-d_6) δ 162.97, 157.14, 152.03, 151.54, 151.17, 149.81, 147.40, 145.97, 138.38, 135.22, 131.95, 130.38, 128.49, 127.81, 126.58, 126.23, 121.80, 121.30, 117.34, 114.52, 106.08, 54.47, 49.29, 47.34, 45.69, 11.22, 3.42 ppm. **HRMS**: m/z calc. for $[C_{30}H_{32}O_2N_6+H]^+$ 509.26595, found 509.26544, calc. for $[C_{30}H_{32}O_2N_6+Na]^+$ 531.24790, found 531.24714.

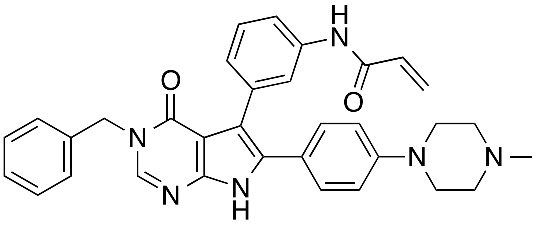

N-(3-{3-Benzyl-6-[4-(4-methylpiperazin-1-yl)phenyl]-4-oxo-4,7-dihydro-3H-pyrrolo[2,3-d]pyrimidin-5-yl}phenyl)acrylamide (29l)

According to the general method, Mitsunobu derivatization, starting from compound **27a** and with benzyl alcohol as substrate.

Yield: 62% (23.1 mg, 41.7 µmol) over two steps. **^{1}H NMR** (500 MHz, DMSO-d_6) δ 12.21 (s, 1H), 10.07 (s, 1H), 8.37 (s, 1H), 7.74 (d, *J* = 8.1 Hz, 1H), 7.50 (s, 1H), 7.35–7.23 (m, 5H), 7.21 (t, *J* = 7.9 Hz, 1H), 7.16 (d, *J* = 8.9 Hz, 2H), 6.93 (d, *J* = 7.6 Hz, 1H), 6.83 (d, *J* = 8.9 Hz, 2H), 6.43 (dd, *J* = 17.0, 10.2 Hz, 1H), 6.22 (dd, *J* = 17.0, 1.8 Hz, 1H), 5.71 (dd, *J* = 10.2, 1.8 Hz, 1H), 5.14 (s, 2H), 3.14 (br s, 4H), 2.43 (s, 4H), 2.21 ppm (s, 3H). **^{13}C NMR** (126 MHz, DMSO-d_6) δ 162.97, 156.96, 149.80, 147.31, 146.18, 138.40, 137.71, 135.08, 131.95, 130.57, 128.51, 128.48, 127.83, 127.37, 127.33, 126.57, 126.21, 121.73, 121.23, 117.37, 114.62, 114.54, 106.15, 54.39, 47.96, 47.24, 45.57, 39.52 ppm. **HRMS**: *m/z* calc. for $[C_{33}H_{32}O_2N_6+H]^+$ 545.26595, found 545.26681, calc. for $[C_{33}H_{32}O_2N_6+Na]^+$ 567.24790, found 567.24809.

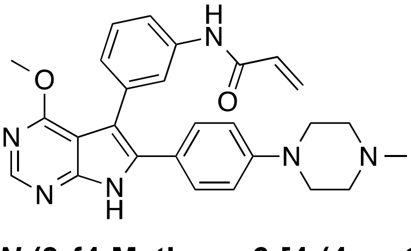

N-(3-{4-Methoxy-6-[4-(4-methylpiperazin-1-yl)phenyl]-7H-pyrrolo[2,3-d]pyrimidin-5-yl}phenyl)acrylamide (19a)

According to the general method, Mitsunobu derivatization, starting from compound **27a** and with methanol as substrate.

Yield: 31% (4.0 mg, 8.5 µmol) over two steps. **^{1}H NMR** (500 MHz, DMSO-*d_6*) δ 12.33 (s, 1H), 10.09 (s, 1H), 8.37 (s, 1H), 7.74 (d, *J* = 8.3 Hz, 1H), 7.56 (s, 1H), 7.31–7.21 (m, 4H), 6.99 (d, *J* = 7.7 Hz, 1H), 6.87 (d, *J* = 9.0 Hz, 2H), 6.43 (dd, *J* = 17.0, 10.2 Hz, 1H), 6.23 (dd, *J* = 17.0, 2.0 Hz, 1H), 5.73 (dd, *J* = 10.1, 2.0 Hz, 1H), 3.86 (s, 4H), 3.51 (s, 2H), 3.19 (br s, 4H), 2.31 (br s, 3H). **HRMS**: *m/z* calc. for $[C_{27}H_{28}O_2N_6+H]^+$ 469.23465, found 469.23490, calc. for $[C_{27}H_{28}O_2N_6+Na]^+$ 491.21660, found 491.21668.

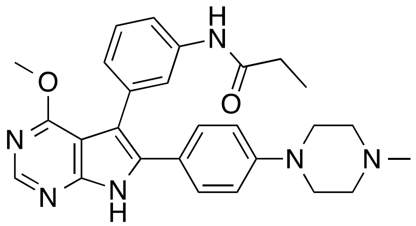

***N*-(3-{3-Methyl-6-[4-(4-methylpiperazin-1-yl)phenyl]-4-oxo-4,7-dihydro-3*H*-pyrrolo[2,3-*d*]pyrimidin-5-yl}phenyl)propionamide (19b)**

According to the general method, Mitsunobu derivatization, starting from compound **27b** and with methanol as substrate.

Yield: 69% (22.2 mg, 47.2 µmol) over two steps. **^{1}H NMR** (500 MHz, DMSO-*d_6*) δ 12.09 (s, 1H), 9.78 (s, 1H), 8.18 (s, 1H), 7.64 (d, *J* = 7.9 Hz, 1H), 7.45 (s, 1H), 7.20–7.13 (m, 3H), 6.88 (d, *J* = 7.6 Hz, 1H), 6.83 (d, *J* = 8.9 Hz, 2H), 3.41 (s, 3H), 3.17 (br s, 4H), 2.29 (br s, 4H), 1.06 ppm (t, *J* = 7.5 Hz, 3H). **^{13}C NMR** (126 MHz, DMSO-*d_6*) δ 171.78, 157.53, 149.56, 147.52, 146.32, 138.78, 135.13, 130.13, 128.42, 127.67, 125.52, 121.57, 121.48, 117.03, 114.62, 114.56, 106.01, 54.16, 47.02, 33.04, 29.45, 9.64 ppm. **HRMS**: *m/z* calc. for $[C_{27}H_{30}O_2N_6+H]^+$ 471.25030, found 471.25032.

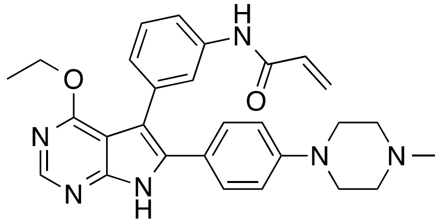

N-(3-{4-Ethoxy-6-[4-(4-methylpiperazin-1-yl)phenyl]-7H-pyrrolo[2,3-d]pyrimidin-5-yl}phenyl)acrylamide (19c)

Compound **28b** (20.0 mg, 32.6 µmol, 1.0 eq.) was taken up in CH_2Cl_2 (30 mM) and to this was added TFA (2 mL). The reaction mixture was stirred at rt until full conversion (3 h). After removal of the solvent *in vacuo*, the crude residue was re-dissolved in THF (30 mM) followed by the addition of an aqueous solution of NaOH (1 M, 2 mL) and the reaction mixture then allowed to stir for a further 1 h. To this mixture CH_2Cl_2 was added and the mixture extracted with aliquots of 5% MeOH in CH_2Cl_2. The combined organic layers were dried over $MgSO_4$ and the solvent removed *in vacuo*. Purification by column chromatography yielded the desired compound as a white solid.

Yield: 75% (9.1 mg, 18.9 µmol). **^{1}H NMR** (DMSO-d_6, 500 MHz) δ 12.12 (s, 1H), 10.07 (br s, 1H), 8.20 (s, 1H), 7.69 (d, *J* = 8.4 Hz, 1H), 7.51 (s, 1H), 7.22 (t, *J* = 8.0 Hz, 1H), 7.16 (d, *J* = 9.2 Hz, 2H), 6.95 (d, *J* = 7.7 Hz, 1H), 6.83 (d, *J* = 9.2 Hz, 2H), 6.42 (dd, *J* = 16.8, 10.3 Hz, 1H), 6.23 (d, *J* = 16.8 Hz, 1H), 5.72 (d, *J* = 10.3 Hz, 1H), 4.35 (q, *J* = 6.9 Hz, 2H), 3.13 (t, *J* = 4.6 Hz, 4H), 2.42 (br s, 4H), 2.21 (s, 3H), 1.16 ppm (t, *J* = 6.9 Hz, 3H). **HRMS**: *m/z* calc. for $[C_{28}H_{30}O_2N_6+H]^+$ 483.25030, found 483.24926.

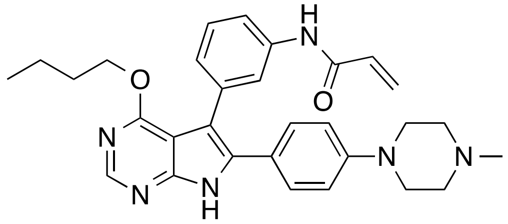

***N*-(3-{4-Butoxy-6-[4-(4-methylpiperazin-1-yl)phenyl]-7*H*-pyrrolo[2,3-*d*]pyrimidin-5-yl}phenyl)acrylamide (19d)**

According to the general method, Mitsunobu derivatization, starting from compound **27a** and with butan-1-ol as substrate.

Yield: 39% (16.8 mg, 32.9 µmol) over two steps. **^{1}H NMR** (500 MHz, DMSO-d_6) δ 12.12 (s, 1H), 10.08 (s, 1H), 8.18 (s, 1H), 7.74 (d, J = 8.1 Hz, 1H), 7.51 (s, 1H), 7.22 (t, J = 7.9 Hz, 1H), 7.17 (d, J = 8.9 Hz, 2H), 6.94 (d, J = 7.6 Hz, 1H), 6.83 (d, J = 8.9 Hz, 2H), 6.44 (dd, J = 17.0, 10.2 Hz, 1H), 6.23 (dd, J = 17.0, 1.9 Hz, 1H), 5.72 (dd, J = 10.2, 1.8 Hz, 1H), 3.90 (t, J = 7.2 Hz, 2H), 3.14 (br s, 4H), 2.44 (br s, 4H), 2.22 (s, 3H), 1.65–1.55 (m, 2H), 1.33–1.25 (m, 2H), 0.89 (t, J = 7.4 Hz, 3H). **^{13}C NMR** (126 MHz, DMSO-d_6) δ 162.98, 161.95, 152.13, 150.18, 149.98, 138.62, 135.40, 132.83, 131.96, 128.82, 127.90, 126.56, 126.02, 121.77, 121.04, 117.22, 114.54, 110.25, 104.94, 65.09, 54.18, 46.94, 45.25, 30.33, 18.59, 13.54 ppm. **HRMS**: *m/z* calc. for $[C_{30}H_{34}O_2N_6+H]^+$ 511.28160, found 511.28136, calc. for $[C_{30}H_{34}O_2N_6+Na]^+$ 533.26355, found 533.26286.

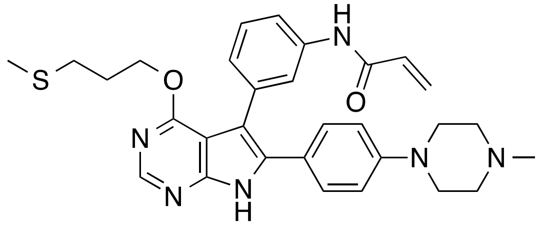

***N*-(3-{6-[4-(4-methylpiperazin-1-yl)phenyl]-4-[3-(methylthio)propoxy]-7*H*-pyrrolo[2,3-*d*]pyrimidin-5-yl}phenyl)acrylamide (19e)**

According to the general method, Mitsunobu derivatization, starting from compound **27a** and with 3-(methylthio)propan-1-ol as substrate.

Yield: 13% (7.1 mg, 13.1 µmol) over two steps. **^{1}H NMR** (500 MHz, DMSO-d_6) δ 12.34 (s, 1H), 10.13 (s, 1H), 8.34 (s, 1H), 7.70 (d, *J* = 8.1 Hz, 1H), 7.66 (s, 1H), 7.29–7.24 (m, 3H), 6.98 (d, *J* = 7.5 Hz, 1H), 6.87 (d, *J* = 8.9 Hz, 2H), 6.42 (dd, *J* = 17.0, 10.2 Hz, 1H), 6.22 (dd, *J* = 17.0, 1.8 Hz, 1H), 5.72 (dd, *J* = 10.2, 1.8 Hz, 1H), 4.34 (t, *J* = 5.8 Hz, 2H), 3.50 (s, 2H), 3.17 (br s, 4H), 2.44 (br s, 4H), 2.26–2.21 (m, 2H), 1.91 (s, 3H), 1.80–1.73 ppm (m, 3H). **HRMS**: *m/z* calc. for $[C_{30}H_{34}O_2N_6S+H]^+$ 543.25367, found 543.25335.

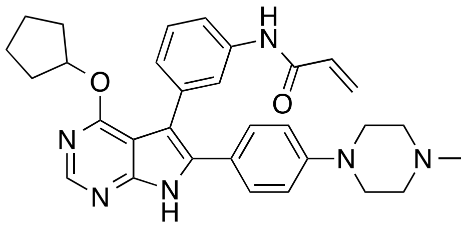

***N*-(3-{4-(Cyclopentyloxy)-6-[4-(4-methylpiperazin-1-yl)phenyl]-7*H*-pyrrolo[2,3-*d*]pyrimidin-5-yl}phenyl)acrylamide (19f)**

According to the general method, Mitsunobu derivatization, starting from compound **27a** and with cyclopentanol as substrate.

Yield: 23% (7.6 mg, 14.6 µmol) over two steps. **^{1}H NMR** (500 MHz, DMSO-d_6) δ 12.24 (s, 1H), 10.07 (s, 1H), 8.32 (s, 1H), 7.66 (d, *J* = 8.6 Hz, 2H), 7.28 (d, *J* = 9.0 Hz, 2H), 7.24 (t, *J* = 7.9 Hz, 1H), 6.96 (d, *J* = 7.7 Hz, 1H), 6.87 (d, *J* = 9.0 Hz, 2H), 6.43 (dd, *J* = 17.0, 10.2 Hz, 1H), 6.23 (dd, *J* = 17.0, 2.0 Hz, 1H), 5.72 (dd, *J* = 10.2, 2.0 Hz, 1H), 5.54–5.50 (m, 1H), 3.17 (br s, 4H), 2.49–2.43 (m, 4H), 2.26 (br s, 3H), 1.78–1.70 (m, 2H), 1.60–1.52 (m, 2H), 1.44–1.38 ppm (m, 4H). **HRMS**: *m/z* calc. for $[C_{31}H_{34}O_2N_6+H]^+$ 523.28160, found 523.28244, calc. for $[C_{31}H_{34}O_2N_6+Na]^+$ 545.26355, found 545.26432.

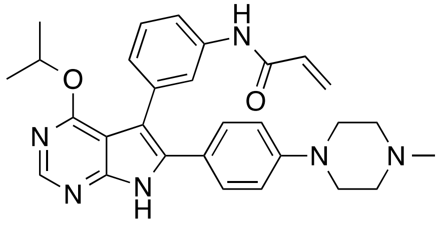

***N*-(3-{4-Isopropoxy-6-[4-(4-methylpiperazin-1-yl)phenyl]-7*H*-pyrrolo[2,3-*d*]pyrimidin-5-yl}phenyl)acrylamide (19g)**

According to the general method, Mitsunobu derivatization, starting from compound **27a** and with propan-2-ol as substrate.

Yield: 43% (14.5 mg, 29.2 µmol) over two steps. **^{1}H NMR** (500 MHz, DMSO-d_6) δ 12.24 (s, 1H), 10.06 (s, 1H), 8.33 (s, 1H), 7.68 (s, 1H), 7.65 (d, *J* = 8.1 Hz, 1H), 7.27 (d, *J* = 8.9 Hz, 2H), 7.24 (t, *J* = 7.9 Hz, 1H), 6.97 (d, *J* = 7.6 Hz, 1H), 6.87 (d, *J* = 8.9 Hz, 2H), 6.44 (dd, *J* = 17.0, 10.2 Hz, 1H), 6.23 (dd, *J* = 17.0, 1.9 Hz, 1H), 5.72 (dd, *J* = 10.2, 1.9 Hz, 1H), 5.33 (spt, *J* = 6.1 Hz, 1 H), 3.18 (br s, 4H), 2.49–2.46 (m, 4H), 2.25 (s, 3H), 1.17 (s, 3H), 1.16 ppm (s, 3H). **^{13}C NMR** (126 MHz, DMSO-d_6) δ 163.00, 161.47, 152.32, 150.18, 150.05, 138.37, 135.14, 132.85, 131.95, 128.93, 127.71, 126.58, 126.25, 122.04, 121.05, 117.22, 114.53, 110.28, 105.04, 68.18, 54.26, 47.04, 45.39, 21.58 ppm. **HRMS**: *m/z* calc. for $[C_{29}H_{32}O_2N_6+H]^+$ 497.26595, found 497.26679.

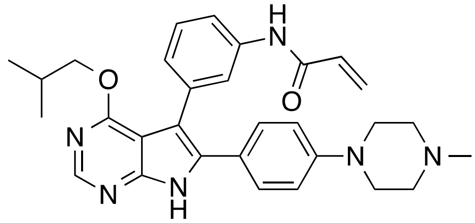

***N*-(3-{4-Isobutoxy-6-[4-(4-methylpiperazin-1-yl)phenyl]-7*H*-pyrrolo[2,3-*d*]pyrimidin-5-yl}phenyl)acrylamide (19h)**

According to the general method, Mitsunobu derivatization, starting from compound **27a** and with 2-methylpropan-1-ol as substrate.

Yield: 39% (17.3 mg, 33.9 µmol) over two steps. **^{1}H NMR** (500 MHz, DMSO-d_6) δ 12.28 (s, 1H), 10.08 (s, 1H), 8.32 (s, 1H), 7.70 (d, J = 8.4 Hz, 1H), 7.63 (s, 1H), 7.31–7.24 (m, 3H), 7.00 (d, J = 7.6 Hz, 1H), 6.86 (d, J = 8.9 Hz, 2H), 6.42 (dd, J = 17.0, 10.1 Hz, 1H), 6.22 (dd, J = 17.0, 1.8 Hz, 1H), 5.72 (dd, J = 10.2, 1.8 Hz, 1H), 4.07 (d, J = 6.1 Hz, 2H), 3.17 (br s, 4H), 2.46 (br s, 4H), 2.24 (s, 3H), 1.79 (dp, J = 13.0, 6.5 Hz, 1H), 0.72 (s, 3H), 0.71 ppm (s, 3H). **^{13}C NMR** (126 MHz, DMSO-d_6) δ 163.00, 152.10, 150.14, 150.03, 138.64, 135.60, 132.80, 131.93, 128.72, 128.05, 126.58, 126.09, 121.71, 120.97, 117.41, 117.38, 114.51, 110.23, 105.07, 71.55, 54.28, 47.05, 45.44, 27.33, 18.70 ppm. **HRMS**: *m/z* calc. for $[C_{30}H_{34}O_2N_6+H]^+$ 511.28160, found 511.28213, calc. for $[C_{30}H_{34}O_2N_6+Na]^+$ 533.26355, found 533.26369.

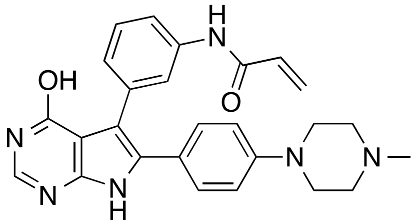

***N*-(3-{6-[4-(4-Methylpiperazin-1-yl)phenyl]-4-oxo-4,7-dihydro-3*H*-pyrrolo[2,3-*d*]pyrimidin-5-yl}phenyl)acrylamide (19i)**

Compound **27a** (30.2 mg, 51.5 µmol, 1.0 eq.) was taken in CH_2Cl_2 (30 mM) and was added TFA (2 mL). The reaction mixture was stirred at rt until full conversion (3 h). After removal of the solvent *in vacuo*, the crude residue was re-dissolved in THF (30 mM), followed by addition of an aqueous solution of NaOH (1 M, 2 mL) and the reaction mixture allowed to stir for a further 1 h. To this was added CH_2Cl_2 and the mixture was then extracted with aliquots of 5% MeOH in CH_2Cl_2. The combined organic layers were dried over $MgSO_4$ and the solvent removed *in vacuo*. Purification by column chromatography yielded the desired compound as a white solid.

Yield: 38% (8.9 mg, 19.6 µmol) over two steps. **^{1}H NMR** (500 MHz, CD_3OD) δ 7.92 (s, 1H), 7.73 (d, J = 8.2 Hz, 1H), 7.59 (s, 1H), 7.32 (d, J = 8.8 Hz, 2H), 7.27 (t, J = 7.9 Hz, 1H), 7.12 (d, J = 7.5

Hz, 1H), 6.98 (d, *J* = 8.8 Hz, 2H), 6.44 (dd, *J* = 17.0, 10.0 Hz, 1H), 6.35 (dd, *J* = 17.0, 1.6 Hz, 1H), 5.77 (dd, *J* = 10.1, 1.6 Hz, 1H), 3.42 (br s, 4H), 2.96 (s, 3H), 1.32 ppm (s, 4H). **^{1}H NMR** (500 MHz, DMSO-d_6) δ 12.18 (s, 1H), 11.77 (s, 1H), 10.17 (s, 1H), 7.85 (d, *J* = 3.7 Hz, 1H), 7.74 (d, *J* = 7.9 Hz, 1H), 7.54 (s, 1H), 7.36 (s, 1H), 7.26 (s, 1H), 7.16 (s, 1H), 6.95 (d, *J* = 7.7 Hz, 1H), 6.91 (d, *J* = 8.8 Hz, 1H), 6.47 (dd, *J* = 17.0, 10.2 Hz, 1H), 6.22 (dd, *J* = 17.0, 1.7 Hz, 1H), 5.72 (dd, *J* = 10.3, 1.4 Hz, 1H), 3.17 (br s, 4H), 3.09 (br s, 4H), 2.77 ppm (s, 3H). **HRMS**: *m/z* calc. for $[C_{26}H_{26}O_2N_6+H]^+$ 455.21900, found 455.21852, calc. for $[C_{26}H_{26}O_2N_6+Na]^+$ 477.20095, found 477.20035.
